# Prediction of homoprotein and heteroprotein complexes by protein docking and template‐based modeling: A CASP‐CAPRI experiment

**DOI:** 10.1002/prot.25007

**Published:** 2016-06-01

**Authors:** Marc F. Lensink, Sameer Velankar, Andriy Kryshtafovych, Shen‐You Huang, Dina Schneidman‐Duhovny, Andrej Sali, Joan Segura, Narcis Fernandez‐Fuentes, Shruthi Viswanath, Ron Elber, Sergei Grudinin, Petr Popov, Emilie Neveu, Hasup Lee, Minkyung Baek, Sangwoo Park, Lim Heo, Gyu Rie Lee, Chaok Seok, Sanbo Qin, Huan‐Xiang Zhou, David W. Ritchie, Bernard Maigret, Marie‐Dominique Devignes, Anisah Ghoorah, Mieczyslaw Torchala, Raphaël A.G. Chaleil, Paul A. Bates, Efrat Ben‐Zeev, Miriam Eisenstein, Surendra S. Negi, Zhiping Weng, Thom Vreven, Brian G. Pierce, Tyler M. Borrman, Jinchao Yu, Françoise Ochsenbein, Raphaël Guerois, Anna Vangone, João P.G.L.M. Rodrigues, Gydo van Zundert, Mehdi Nellen, Li Xue, Ezgi Karaca, Adrien S.J. Melquiond, Koen Visscher, Panagiotis L. Kastritis, Alexandre M.J.J. Bonvin, Xianjin Xu, Liming Qiu, Chengfei Yan, Jilong Li, Zhiwei Ma, Jianlin Cheng, Xiaoqin Zou, Yang Shen, Lenna X. Peterson, Hyung‐Rae Kim, Amit Roy, Xusi Han, Juan Esquivel‐Rodriguez, Daisuke Kihara, Xiaofeng Yu, Neil J. Bruce, Jonathan C. Fuller, Rebecca C. Wade, Ivan Anishchenko, Petras J. Kundrotas, Ilya A. Vakser, Kenichiro Imai, Kazunori Yamada, Toshiyuki Oda, Tsukasa Nakamura, Kentaro Tomii, Chiara Pallara, Miguel Romero‐Durana, Brian Jiménez‐García, Iain H. Moal, Juan Férnandez‐Recio, Jong Young Joung, Jong Yun Kim, Keehyoung Joo, Jooyoung Lee, Dima Kozakov, Sandor Vajda, Scott Mottarella, David R. Hall, Dmitri Beglov, Artem Mamonov, Bing Xia, Tanggis Bohnuud, Carlos A. Del Carpio, Eichiro Ichiishi, Nicholas Marze, Daisuke Kuroda, Shourya S. Roy Burman, Jeffrey J. Gray, Edrisse Chermak, Luigi Cavallo, Romina Oliva, Andrey Tovchigrechko, Shoshana J. Wodak

**Affiliations:** ^1^ University LilleCNRS UMR8576 UGSFLilleF‐59000France; ^2^ European Molecular Biology LaboratoryEuropean Bioinformatics Institute (EMBL‐EBI)Wellcome Trust Genome CampusHinxtonCambridgeCB10 1SDUnited Kingdom; ^3^ Genome CenterUniversity of CaliforniaDavisCalifornia95616; ^4^ Research Support Computing, University of Missouri Bioinformatics Consortium, and Department of Computer Science, University of MissouriColumbiaMissouri65211; ^5^ Department of Bioengineering and Therapeutic SciencesUniversity of California San FranciscoSan FranciscoCalifornia94158; ^6^ Department of Pharmaceutical ChemistryUniversity of California San FranciscoSan FranciscoCalifornia94158; ^7^ California Institute for Quantitative Biosciences (QB3)University of California San FranciscoSan FranciscoCalifornia94158; ^8^ GN7 of the National Institute for Bioinformatics (INB) and Biocomputing Unit, National Center of Biotechnology (CSIC)Madrid28049Spain; ^9^ Institute of Biological, Environmental and Rural Sciences (IBERS), Aberystwyth UniversityAberystwythSY233FGUnited Kingdom; ^10^ Department of Computer ScienceUniversity of Texas at AustinAustinTexas78712; ^11^ Institute for Computational Engineering and Sciences, University of Texas at AustinAustinTexas78712; ^12^ Department of ChemistryUniversity of Texas at AustinAustinTexas78712; ^13^ LJK, University Grenoble Alpes, CNRSGrenoble38000France; ^14^ INRIAGrenoble38000France; ^15^ Moscow Institute of Physics and TechnologyDolgoprudniyRussia; ^16^ Department of ChemistrySeoul National UniversitySeoul151‐747Republic of Korea; ^17^ Department of Physics and Institute of Molecular BiophysicsFlorida State UniversityTallahasseeFlorida32306USA; ^18^ INRIA Nancy—Grand EstVillers‐lès‐Nancy54600France; ^19^ CNRS, LORIACampus Scientifique, BP 239Vandœuvre‐lès‐Nancy54506France; ^20^ Department of Computer Science and EngineeringUniversity of MauritiusReduitMauritius; ^21^ Biomolecular Modelling Laboratory, the Francis Crick Institute, Lincoln's Inn Fields LaboratoryLondonWC2A 3LYUnited Kingdom; ^22^ G‐INCPM, Weizmann Institute of ScienceRehovot7610001Israel; ^23^ Department of Chemical Research SupportWeizmann Institute of ScienceRehovot7610001Israel; ^24^ Sealy Center for Structural Biology and Molecular Biophysics, University of Texas Medical Branch301 University BoulevardGalvestonTexas77555‐0857; ^25^ Program in Bioinformatics and Integrative Biology, University of Massachusetts Medical SchoolWorcesterMassachusetts01605; ^26^ Institute for Integrative Biology of the Cell (I2BC), CEA, CNRS, University Paris‐Saclay, CEA‐SaclayGif‐sur‐Yvette91191France; ^27^ Bijvoet Center for Biomolecular Research, Faculty of Science – Chemistry, Utrecht UniversityPadualaan 8Utrecht3584 CHThe Netherlands; ^28^ Dalton Cardiovascular Research Center, University of MissouriColumbiaMissouri65211; ^29^ Department of Physics and AstronomyUniversity of MissouriColumbiaMissouri65211; ^30^ Department of Computer ScienceUniversity of MissouriColumbiaMissouri65211; ^31^ Informatics Institute, University of MissouriColumbiaMissouri65211; ^32^ Department of BiochemistryUniversity of MissouriColumbiaMissouri65211; ^33^ Toyota Technological Institute at Chicago6045 S Kenwood AvenueChicagoIllinois60637; ^34^ Department of Biological SciencesPurdue UniversityWest LafayetteIndiana47907; ^35^ Bioinformatics and Computational Biosciences Branch, Rocky Mountain Laboratories, National Institutes of HealthHamiltonMontano 59840; ^36^ Department of Computer SciencePurdue UniversityWest LafayetteINUSA47907; ^37^ Molecular and Cellular Modeling GroupHeidelberg Institute for Theoretical Studies (HITS)HeidelbergGermany; ^38^ Center for Molecular Biology (ZMBH), DKFZ‐ZMBH Alliance, Heidelberg UniversityHeidelbergGermany; ^39^ Interdisciplinary Center for Scientific Computing (IWR), Heidelberg UniversityHeidelbergGermany; ^40^ Center for Computational Biology, The University of KansasLawrenceKansas66047; ^41^ Department of Molecular BiosciencesThe University of KansasLawrenceKansas66047; ^42^ Computational Biology Research Center (CBRC), National Institute of Advanced Industrial Science and Technology (AIST)Koto‐KuJapan; ^43^ Graduate School of Frontier Sciencesthe University of TokyoKashiwaJapan; ^44^ Joint BSC‐CRG‐IRB Research Program in Computational Biology, Barcelona Supercomputing CenterC/Jordi Girona 29Barcelona08034Spain; ^45^ Center for in‐Silico Protein Science, Korea Institute for Advanced StudySeoul130‐722Korea; ^46^ Center for Advanced Computation, Korea Institute for Advanced StudySeoul130‐722Korea; ^47^ School of Computational ScienceKorea Institute for Advanced StudySeoul130‐722Korea; ^48^ Department of Biomedical EngineeringBoston UniversityBostonMassachusetts; ^49^ Department of ChemistryBoston UniversityBostonMassachusetts; ^50^ Institute of Biological Diversity, International Pacific Institute of IndianaBloomingtonIndiana47401; ^51^ Drosophila Genetic Resource Center, Kyoto Institute of TechnologyUkyo‐Ku616‐8354Japan; ^52^ International University of Health and Welfare Hospital (IUHW Hospital)Asushiobara‐City, Tochigi Prefecture329‐2763Japan; ^53^ Department of Chemical and Biomolecular EngineeringJohns Hopkins UniversityBaltimoreMaryland21218; ^54^ Program in Molecular Biophysics, Johns Hopkins UniversityBaltimoreMaryland21218; ^55^ King Abdullah University of Science and TechnologySaudi Arabia; ^56^ University of Naples “Parthenope”NapoliItaly; ^57^ J. Craig Venter Institute9704 Medical Center DriveRockvilleMaryland20850; ^58^ Departments of Biochemistry and Molecular GeneticsUniversity of TorontoTorontoOntarioCanada; ^59^ VIB Structural Biology Research CenterVUB Pleinlaan 2Brussels1050Belgium; ^60^ Present address: Tyler M. Borrman current address is Institute for Bioscience and Biotechnology Research, University of MarylandRockvilleMD20850; ^61^ Present address: Yang Shen current address is Center for Bioinformatics and Genomic Systems Engineering, Department of Electrical and Computer Engineering, Texas a&M UniversityCollege StationTX77843; ^62^ Present address: Kenichiro Imai, Toshiyuki Oda, and Kentaro Tomii current address is Biotechnology Research Institute for Drug Discovery, National Institute of Advanced Industrial Science and Technology (AIST)Koto‐KuJapan; ^63^ Present address: Kazunori Yamada current address is Group of Electrical Engineering, Communication Engineering, Electronic Engineering, and Information Engineering, Tohoku UniversitySendaiJapan; ^64^ Present address: Iain H. Moal current address is European Molecular Biology Laboratory, European Bioinformatics Institute (EMBL‐EBI), Wellcome Trust Genome CampusHinxtonCambridgeCB10 1SDUnited Kingdom; ^65^ Present address: Daisuke Kuroda current address is School of Pharmacy, Showa UniversityShinagawa‐KuTokyo142‐8555Japan

**Keywords:** CAPRI, CASP, oligomer state, blind prediction, protein interaction, protein docking

## Abstract

We present the results for CAPRI Round 30, the first joint CASP‐CAPRI experiment, which brought together experts from the protein structure prediction and protein–protein docking communities. The Round comprised 25 targets from amongst those submitted for the CASP11 prediction experiment of 2014. The targets included mostly homodimers, a few homotetramers, and two heterodimers, and comprised protein chains that could readily be modeled using templates from the Protein Data Bank. On average 24 CAPRI groups and 7 CASP groups submitted docking predictions for each target, and 12 CAPRI groups per target participated in the CAPRI scoring experiment. In total more than 9500 models were assessed against the 3D structures of the corresponding target complexes. Results show that the prediction of homodimer assemblies by homology modeling techniques and docking calculations is quite successful for targets featuring large enough subunit interfaces to represent stable associations. Targets with ambiguous or inaccurate oligomeric state assignments, often featuring crystal contact‐sized interfaces, represented a confounding factor. For those, a much poorer prediction performance was achieved, while nonetheless often providing helpful clues on the correct oligomeric state of the protein. The prediction performance was very poor for genuine tetrameric targets, where the inaccuracy of the homology‐built subunit models and the smaller pair‐wise interfaces severely limited the ability to derive the correct assembly mode. Our analysis also shows that docking procedures tend to perform better than standard homology modeling techniques and that highly accurate models of the protein components are not always required to identify their association modes with acceptable accuracy. Proteins 2016; 84(Suppl 1):323–348. © 2016 The Authors Proteins: Structure, Function, and Bioinformatics Published by Wiley Periodicals, Inc.

## INTRODUCTION

Most cellular processes are carried out by physically interacting proteins.^1^ Characterizing protein interactions and higher order assemblies is therefore a crucial step in gaining an understanding of how cells function.

Regrettably, protein assemblies are still poorly represented in the Protein Databank (PDB).^2^ Determining the structures of such assemblies has so far been hampered by the difficulty in obtaining suitable crystals and diffraction data. But this limitation is being circumvented with the advent of new powerful electron microscopy techniques, which now enable the structure determinations of very large macromolecular assemblies at atomic resolutions.^3^

On the other hand, the repertoire of individual protein 3D structures has been increasingly filled, thanks to large‐scale structural genomics projects such as the PSI (http://sbkb.org/) and others (http://www.thesgc.org/). Given a newly sequenced protein, the odds are high that its 3D structure can be readily extrapolated from structures of related proteins deposited in the PDB.^4^, ^5^ Moreover, thanks to the recent explosion of the number of available protein sequences, it is now becoming possible to model the structures of individual proteins with increasing accuracy from sequence information alone^6^, ^7^ as will be highlighted in the CASP11 results in this issue. Structures from this increasingly rich repertoire may be used as templates or scaffolds in protein design projects that have useful medical applications.^8^, ^9^ Larger protein assemblies can be modeled by integrating information on individual structures with various other types of data with the help of hybrid modeling techniques.^10^

Computational approaches play a major role in all these endeavors. Of particular importance are methods for deriving accurate structural models of multiprotein assemblies, starting from the atomic coordinates of the individual components, the so‐called “docking” algorithms, and the associated energetic criteria for singling out stable binding modes.^11^, ^12^, ^13^

Taking its inspiration from CASP, the community‐wide initiative on the Critical Assessment of Predicted Interactions (CAPRI), established in 2001, has been designed to test the performance of docking algorithms (http://www.ebi.ac.uk/msd-srv/capri/). Just as CASP has fostered the development of methods for the prediction of protein structures, CAPRI has played an important role in advancing the field of modeling protein assemblies. Initially focusing on protein–protein docking and scoring procedures, CAPRI has expanded its horizon by including targets representing protein‐peptide and protein nucleic acids complexes. It has moreover conducted experiments aimed at evaluating the ability of computational methods to estimate binding affinity of protein–protein complexes^14^, ^15^, ^16^ and to predict the positions of water molecules at the interfaces of protein complexes.^17^

Considering the importance of macromolecular assemblies, and the new opportunities offered by the recent progress in both experimental and computational techniques to probe and model these assemblies, a better integration of the different computational approaches for modeling macromolecular assemblies and their building blocks was called for. Establishing closer ties between the CASP and CAPRI communities appeared as an important step in this direction, inaugurated by running a joint CASP‐CAPRI prediction experiment in the summer of 2014. The results of this experiment were summarized at the CASP11 meeting held in Dec 2014 in Cancun Mexico, and are presented in detail in this report.

The CASP11‐CAPRI experiment, representing CAPRI Round 30, comprised 25 targets for which predictions of protein complexes were assessed. These targets represented a subset of the 100 regular CASP11 targets. This subset comprised only “easy” CASP targets, those whose 3D structure could be readily modeled using standard homology modeling techniques. Targets that required more sophisticated approaches (*ab‐initio* modeling, or homology modeling using very distantly related templates) were not considered, as the CAPRI community had little experience with these approaches. The vast majority of the targets were homo‐oligomers. CAPRI groups were given the choice of modeling the subunit structures of these complexes themselves, or using models made available by CASP participant, in time of the docking calculations.

On average, about 25 CAPRI groups, and about 7 CASP groups submitted docking predictions for each target. About 12 CAPRI scorer groups per target participated in the CAPRI scoring experiment, where participants are invited to single out correct models from an ensemble of anonymized predicted complexes generated during the docking experiment.

In total, these groups submitted >9500 models that were assessed against the 3D structures of the corresponding targets. The assessment was performed by the CAPRI assessment team, using the standard CAPRI model quality measures.^18^, ^19^ A major issue for the assessment, and for the Round as a whole, was the uncertainties in the oligomeric state assignments for a significant number of the targets. For many of these the assigned state at the time of the experiment was inferred solely from the crystal contacts by computational methods, which can be unreliable.

In presenting the CAPRI Round 30 assessment results here, we highlight this issue and the more general challenge of correctly predicting the association modes of weaker complexes of identical subunits, and those of higher order homo‐oligomers. In addition, we examine the influence of the accuracy of the modeled subunits on the performance of the docking and scoring predictions, and evaluate the extent to which docking procedures confer an advantage over standard homology modeling methods in predicting homo‐oligomer complexes.

## THE TARGETS

The 25 targets of the joint CASP‐CAPRI experiment are listed in Table 1. Of these 23 are homo‐oligomers, with 18 declared to be dimers and five to be tetramers, and two heterocomplexes. Hence for the majority of the targets (23) the goal was to model the interface (or interfaces in the case of tetramers) between identical subunits, whose size varied between 44 and 669 residues but was of ∼250 residues on average. The majority of the targets were obtained from structural genomics consortia. They represented mainly microbial proteins, whose function was often annotated as putative.

**Table 1:** The CAPRI‐CASP11 Targets of CAPRI Round 30

Target ID		Contributor	Quaternary state		Residues	Buried area (Å^2^)	Protein
CAPRI	CASP		Author	PISA			
T68	T0759	NSGC	**1 or 2**	**1**	109	860	Plectin 1 and 2 repeats (HR9083A) of the Human Periplakin
T69	T0764	JCSG	2	**2**	341	2415	Putative esterase (BDI_1566) from Parabacteroides distasonis
T70	T0765	JCSG	**2**	**4**	128	2030	Modulator protein MzrA (KPN_03524) from Klebsiella pneumoniae subsp.
T71	T0768	JCSG	**4**	**4**	170	2380	Leucine rich repeat protein (BACCAP_00569) from Bacteroides capillosus ATCC 29799
T72	T0770	JCSG	2	**2**	488	1120	SusD homolog (BT2259) from Bacteroides thetaiotaomicron
T73	T0772	JCSG	**4**	**4**	265	5900	Putative glycosyl hydrolase (BDI_3914) from Parabacteroides distasonis
T74	T0774	JCSG	**1**	**4**	379	2040	Hypothetical protein (BVU_2522) from Bacteroides vulgatus
T75	T0776	JCSG	2	**2**	256	1040	Putative GDSL‐like lipase (PARMER_00689) from Parabacteroides merdae (ATCC 43184)
T77	T0780	JCSG	2	**2**	259	1600	Conserved hypothetical protein (SP_1560) from Streptococcus pneumoniae TIGR4
T78	T0786	Non‐SGI	**4**	**4**	264	4160	Hypothetical protein (BCE0241) from Bacillus cereus
T79	T0792	Non‐SGI		**2**	80	680	OSKAR‐N
T80	T0801	NPPB	**2**	2	376	1960	Sugar aminotransferase WecE from Escherichia coli K‐12
T81	T0797	Non‐SGI	2	2	44	1070	cGMP‐dependent protein kinase II leucine zipper
	T0798		2	2	198		Rab11b protein
T82	T0805	Non‐SGI	**2**	2	214	3250	Nitro‐reductase rv3368
T84	T0811	NYSGRC		**2**	255	1740	Triose phosphate isomerase
T85	T0813	NYSGRC	**2**	**2**	307	4620	Cyclohexadienyl dehydrogenase from Sinorhizobium meliloti in complex with NADP
T86	T0815	NYSGRC	**2**	**2**	106	470	Putative polyketide cyclase (protein SMa1630) from Sinorhizobium meliloti
T87	T0819	NYSGRC	**2**	**2**	373	3430	Histidinol‐phosphate aminotransferase from Sinorhizobium meliloti in complex with pyridoxal‐5'‐phosphate
T88	T0825	Non‐SGI	**2**	**2**	205	1350	WRAP‐5
T89	T0840	Non‐SGI	1		669	870	RON receptor tyrosine kinase subunit
	T0841		1		253		Macrophage stimulating protein subunit (MSP)
T90	T0843	MCSG	2	**2**	369	2360	Ats13
T91	T0847	SGC	**1**	**2**	176	1320	Human Bj‐Tsa‐9
T92	T0849	MCSG	**2**	**2**	240	1900	Glutathione S‐transferase domain from Haliangium ochraceum DSM 14365
T93	T0851	MCSG	**2**	**2**	456	2680	Cals8 from Micromonospora echinospora (P294S mutant)
T94	T0852	MCSG	**2**	**2**	414	1190	APC103154

Bold numbers under Quaternary State indicate the oligomeric state assignments available at the time of the prediction experiment; 1 (monomer), 2 (dimer), 4 (tetramer); numbers in regular fonts indicate subsequent assignments collected from the PDB entries for the target structures.

NSGC, Northeast Structural Genomics Consortium; JCSG, Joint Center for Structural Genomics; Non‐SGI, Non‐SGI research Centers and others; NNPB, NatPro PSI:Biology; NYSGRC, New York Structural Genomics Research Center; MCSG, Midwest Center for Structural Genomics; SGC, Structural Genomics Consortium.

Since it is not uncommon for docking approaches to use information on the symmetry of the complex to restrain or filter docking poses, predictors needed to be given reliable information on the biologically/functionally relevant oligomeric state of the target complex to be predicted. While self association between proteins is common, with between 50 and 75% of proteins forming dimers in the cell,^20^, ^21^ this association depends on the binding affinity between the subunits and on their concentration. Information on the oligomeric state is in principle derived using experimental methods such as gel filtration or small‐angle X‐ray scattering (SAXS),^22^ and is usually communicated by the authors upon submission of the atomic coordinates to the PDB. With a majority of the targets being offered by structural genomics consortia before their coordinates were deposited in the PDB, author‐assigned oligomeric states were available to predictors only for a subset (∼15) of the targets, and those were often tentative. For the remaining targets, the oligomeric state was inferred from the crystal contacts using the PISA software,^23^ which although being a widely used standard in the field, may still yield erroneous assignments in a non‐negligible fraction of the cases, as will be shown in this analysis. Such incorrect assignments represented a confounding factor in this CAPRI round, but also allowed to show that docking calculations may help to correct them.

## GLOBAL OVERVIEW OF THE PREDICTION EXPERIMENT

As in typical CAPRI Rounds, CAPRI predictor groups were provided with the amino‐acid sequence of the target protein (for homo‐oligomers), or proteins (for heterocomplexes), and with some relevant details about the protein, communicated by the structural biologists. Using the sequence information, the groups were then invited to model the 3D structure of the protein or proteins, and to derive the atomic structure of the complex. To help with the homology‐modeling task, with which CASP participants are usually more experienced than their CAPRI colleagues, 3D models of individual target proteins predicted by CASP participants were made available to CAPRI groups for use in their docking calculations. A good number of CAPRI groups, but not all, took up this offer.

In addition to submitting 10 models for each target complex, predictors were invited to upload a set of 100 models. Once all the submissions were completed, the uploaded models were shuffled and made available to all groups as part of the CAPRI scoring experiment. The “scorer” groups were in turn invited to evaluate the ensemble of uploaded models using the scoring function of their choice, and submit their own 10 best ranking ones. The typical timelines per target were about 3 weeks for the homology modeling and docking predictions, and 3 days for the scoring experiment.

Table 2 lists for each target the number of groups submitting predictions and the number of models assessed. On average ∼25 CAPRI groups submitted a total of ∼230 models per target, and an average of 12 scorer groups submitted a total of ∼120 models per target. With the exception of three targets, an average of seven groups registered with CASP submitted a total of anywhere between 1 and 33 models for individual targets. CASP predictors participated in larger numbers in the prediction of T88 (T0825) and of the heterocomplexes (T89 – T0840/T0841 and T81 – T0797/T0798), where the CASP targets were defined as the oligomeric structures.

**Table 2:** CAPRI Round 30 Experiment Statistics

				Number of groups				Number of models			
Target ID				CAPRI			CASP	CAPRI			CASP
CAPRI	CASP	PDB	a	Predictors	Uploaders	Scorers	Predictors	Predictors	Uploaders	Scorers	Predictors
T68	T0759	4q28	2	23	10	12	3	221	1000	120	7
T69	T0764	4q34	2	28	10	14	7	266	1000	132	17
T70	T0765	4pwu	2	23	8	13	5	221	710	130	18
T71	T0768	4oju	3	22	9	14	1	214	810	131	1
T72	T0770	4q69	3	25	11	13	4	244	914	130	11
T73	T0772	4qhz	2	23	11	11	7	221	1195	110	16
T74	T0774	4qb7	2	22	11	10	7	202	911	96	11
T75	T0776	4q9a	1	26	12	12	8	253	840	120	21
T76	T0779			*Cancelled – no structure*							
T77	T0780	4qdy	4	24	12	12	6	229	971	120	12
T78	T0786	4qvu	2	24	10	11	5	229	818	110	15
T79	T0792	5a49	3	25	11	12	9	242	900	120	23
T80	T0801	4piw	1	27	10	12	8	264	911	120	27
T81	T0797 T0798	4ojk	1	23	9	11	20	218	641	110	64
T82	T0805	b	1	25	10	12	9	242	911	120	27
T83	T0809			*Cancelled – article from different group online*							
T84	T0811	b	1	25	10	12	10	241	910	120	28
T85	T0813	4wji	1	25	11	12	8	241	920	120	21
T86	T0815	4u13	2	26	11	12	9	251	1010	119	25
T87	T0819	4wbt	1	24	10	12	9	231	894	120	25
T88	T0825	b	1	27	10	13	18	261	910	130	62
T89	T0840 T0841	b	1	22	9	11	55	211	790	110	243
T90	T0843	4xau	1	23	9	11	9	221	811	110	28
T91	T0847	4urj	1	25	9	11	9	242	798	110	24
T92	T0849	4w66	1	23	9	11	9	225	789	110	33
T93	T0851	4wb1	1	22	9	11	8	213	697	110	27
T94	T0852	4w9r	1	22	9	12	8	215	783	120	21

The number of groups corresponds to registered groups that effectively submitted models for the respective target. The number of models represents submitted models, regardless of quality and includes disqualified models. CAPRI groups are allowed to submit no more than their 10 best models, whereas CASP groups are allowed to submit no more than their 5 best models.

a Number of interfaces assessed.

b Not yet released.

Table 2 also lists the uploader groups and the models that they make available for the scoring experiment (100 models per target per uploader group). As detailed above, the uploaded models are complexes output by the docking calculations carried out by individual participants for a given target. Models, uploaded by the different groups, are anonymized, shuffled, and made available to groups solely interested in testing their scoring functions.

## SYNOPSIS OF THE PREDICTION METHODS

Round 30 participants used a wide range of modeling methods and software tools to generate the submitted models. In addition, the approaches used by a given group often differed across targets. Here, we provide only a short synopsis of the main methodological approaches. For a more detailed description of the methods and modeling strategies, readers are referred to the extended Methods Abstracts provided by individual participants (see Supporting Information Table S6).

Templates, representing known structures of homologs to a given target, stored in the PDB, were used in a number of ways. Most commonly, they were employed to model the 3D structures of individual subunits. Some CAPRI participants selected their own templates and used a variety of custom built or well‐established algorithms such as Modeller,^24^ Swiss‐Model,^25^ or ROSETTA,^26^ to model the subunit structures. Others used the models produced by various servers participating in the CASP11 experiment and made available to CAPRI groups, or servers of other groups (HADDOCK^27^). The quality of the CASP server models was usually first assessed using various criteria and the best quality models were selected for the docking calculations. Some groups selected a single best model for a given target, whereas others used several models (sometimes up to five models). Several groups additionally used loop modeling to adjust the conformation of loops regions, and subjected the subunit models to energy refinement.

The majority of CAPRI participants used protein docking and scoring methods to generate and rank candidate complexes. Many employed their own docking methods, some of which were designed to handle symmetric assemblies, whereas others relied on well‐established docking algorithms such as HEX,^28^ ZDock,^29^ RosettaDock,^30^ as well as on docking programs such as MZDock^31^ which apply symmetry constraints.

When templates were available for a given target (mostly for homodimers), some participants used the information from these templates (consensus interface residues, contacts, or relative arrangement of subunits) to guide the docking calculations or to select docking solutions. Others used the dimeric templates directly to model the target dimer (template‐based “docking”^32^, ^33^, ^34^). Less than a hand‐full of groups employed template‐based modeling alone for all or most of the targets.

To model tetrameric targets, most groups proceeded in two steps. They used either known dimeric homologs, or docking methods to build the dimer portion of the tetramer, and then run their docking procedures to generate a dimer‐of‐dimers, representing the predicted tetramer.

## ASSESSMENT PROCEDURES AND CRITERIA

### The standard CAPRI assessment protocol

The predicted homo and heterocomplexes were assessed by the CAPRI assessment team, using the standard CAPRI assessment protocol, which evaluates the correspondence between predicted complex and the target structure.^18^, ^19^

**Figure 1:**
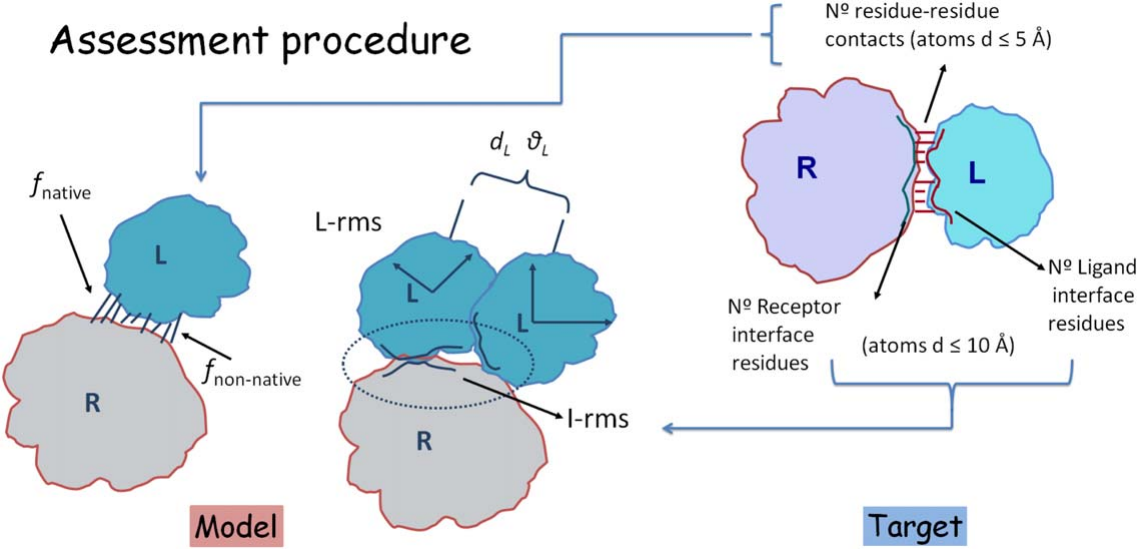
Schematic illustration of the CAPRI assessment criteria. The following quantities were computed for each target: (1) all the residue‐residue contacts between the Receptor (R) and the Ligand (L), and (2) the residues contributing to the interface of each of the components of the complex. Interface residues were defined on the basis of their contribution to the interface area, as described in references.^18^, ^19^ For each submitted model the following quantities were computed: the fractions *f(nat)* of native and *f(non‐nat)* of non‐native contacts in the predicted interface; the root mean square displacement (rmsd) of the backbone atoms of the ligand (*L‐rms*), the mis‐orientation angle *θ*_L_ and the residual displacement *d*_L_ of the ligand center of mass, after the receptor in the model and experimental structures were optimally superimposed. In addition we computed *I‐rms*, the rmsd of the backbone atoms of all interface residues after they have been optimally superimposed. Here the interface residues were defined less stringently on the basis of residue‐residue contacts (see Refs. 18, 19).

This protocol (summarized in Fig. 1) first defines the set of residues common to all the submitted models and the target, so as to enable the comparison of residue‐dependent quantities, such as the root mean square deviation (rmsd) of the models versus the target structure. Models where the sequence identity to the target is too low are not assessed. The threshold is determined on a per‐target basis, but is typically set to 70%.

The set of common residues is used to evaluate the two main rmsd‐based quantities used in the assessment: the ligand rmsd (*L‐rms*) and the interface rmsd (*I‐rms*). *L‐rms* is the backbone rmsd over the common set of ligand residues after a structural superposition of the receptor. *I‐rms* is the backbone rmsd calculated over the common set of interface residues after a structural superposition of these residues. An interface residue is defined as such when any of its atoms (hydrogens excluded) are found within 10 Å of any of the atoms of the binding partner.

An important third quantity whereby models are assessed is *f(nat),* representing the fraction of native contacts in the target, that is, reproduced in the model. This quantity takes all the protein residues into account. A ligand‐receptor contact is defined as any pair of ligand‐receptor atoms within 5 Å distance. Atomic contacts below 3 Å are considered as clashes; predictions with too many clashes are disqualified. The clash threshold varies with the target and is defined as the average number of clashes in the set of predictions plus two standard deviations. The quantities *f(nat), L‐rms* and *I‐rms* together determine the quality of a predicted model, and based on those three parameters models are ranked into four categories: High quality, medium quality, acceptable quality and incorrect, as summarized in Table 3.

**Table 3:** Summary of CAPRI Criteria for Ranking Predicted Complexes

	Score	*f*(nat)	L‐rms		I‐rms
***	High	≥ 0.5	≤ 1.0	OR	≤ 1.0
**	Medium	≥ 0.3	< 1.0–5.0]	OR	< 1.0–2.0]
*	Acceptable	≥ 0.1	< 5.0–10.0]	OR	< 2.0–4.0]
	Incorrect	< 0.1	> 10.0	AND	> 4.0

### Applying the CAPRI assessment protocol to homo‐oligomers

Evaluating models of homo and heteroprotein complexes against the corresponding target structure is a well‐defined problem when the target complex is unambiguously defined, for example, if the target association mode and corresponding interface represents the biologically relevant unit. This is usually, although not always, the case for binary heterocomplexes, but was not the situation encountered in this experiment for the homo‐oligomer targets. All except two of the 25 targets for which predictions were evaluated here represent homo‐oligomers. For about half of these targets the oligomeric state was deemed unreliable, as it was either only inferred computationally from the crystal structure using the PISA software^23^ or because the authors' assignment and inferred oligomeric states, although available, were inconsistent (Table 1). Only about 15 targets had an oligomeric state assigned by the authors at the time of the experiment.

To address this problem in the assessment, the PISA software was used to generate all the crystal contacts for each target and to compute the corresponding interface areas. The interfaces were then ranked according to size of the interface. In candidate dimer targets, submitted models were usually evaluated against 1 or 2 of the largest interfaces of the target, and acceptable or better models for any or all of these interfaces were tallied. For candidate tetramer targets, the relevant largest interfaces for each target were identified in the crystal structure, and predicted models were evaluated by comparing in turn each pair of interacting subunits in the model to each of the relevant pairs of interacting subunits in the target (Supporting Information Fig. S1), and again the best predicted interfaces were retained for the tally. One of the two bonafide heterocomplexes was also evaluated against multiple interfaces.

### Evaluating the accuracy of the 3D models of individual subunits

Since this experiment was a close collaboration between CAPRI and CASP, the quality of the 3D models of individual subunits in the predicted complexes was assessed by the CASP team using the LGA program,^35^ which is the basic tool for model/target comparison in CASP.^36^, ^37^ The tool can be run in two evaluation modes. In the sequence‐dependent mode, the algorithm assumes that each residue in the model corresponds to a residue with the same number in the target, while in the sequence‐independent mode this restriction is not applied. The program searches for optimal superimpositions between two structures at different distance cutoffs and returns two main accuracy scores; GDT_TS and LGA_S. The GDT_TS score is calculated in the sequence‐dependent mode and represents the average percentage of residues that are in close proximity in two structures optimally superimposed using four selected distance cutoffs (see Ref. 38 for details). The LGA_S score is calculated in both evaluation modes and represents a weighted sum of the auxiliary LCS and GDT scores from the superimpositions built for the full set of distance cutoffs (see Ref. 35 for details). We have run the evaluation in both modes, but since the CAPRI submission format permits different residue numbering, we used the LGA_S score from the sequence‐independent analysis as the main measure of the subunit accuracy assessment. This score is expressed on a scale from 0 to 100, with 100 representing a model that perfectly fits the target. The rmsd values for subunit models cited throughout the text are those computed by LGA software. We verified that for about 80% of the assessed models the GDT‐TS and LGA‐S scores differed by <15 units, indicating that these models correspond to near identical structural alignments with the corresponding targets, in line with the fact that the majority of the targets of this Round represent proteins that could be readily modeled by homology. Of the remaining 20% with larger differences between the 2 scores, 18% correspond to disqualified models or incorrect complexes and 2% correspond to acceptable (or higher quality) predicted complexes. Their impact on the analysis is therefore negligible.

### Building target models based on the best available templates

In order to better estimate the added value of protein docking procedures and template‐based modeling techniques it seemed of interest to build a baseline against which the different approaches could be benchmarked. To this end, the best oligomeric structure template for each target available at the time of the predictions was identified. Based on this template, the target model was built using a standard modeling procedure, and the quality of this model was assessed using the CAPRI evaluation criteria described above.

To identify the templates, the protein structure database “PDB70” containing proteins of mutual sequence identity ≤70% was downloaded from HHsuite.^39^ The database was updated twice during the experiment (See Supporting Information Table S5 for the release date of the database used for each target). Only homo‐complexes were considered for this analysis.

The best available templates were detected in three different ways and target models were generated from the templates as follows: (1) *Detection based on sequence information alone*: For each target sequence, proteins related to the target were searched for in the protein structure database by HHsearch^40^ in the local alignment mode with the Viterbi algorithm.^41^ Among the top 100 entries, up to 10 proteins that are in the desired oligomer state were selected as templates. When more than two assembly structures with different interfaces were identified, the best ranking one was selected as template. The target and template sequences were aligned using HHalign^40^ in the global alignment mode with the maximum accuracy algorithm. Based on the sequence alignments, oligomer models were built using MODELLER.^42^ The model with the lowest MODELLER energy out of 10 models was selected for further analysis. (2) *Detection based on the experimental monomer structure*: Proteins with highest structural similarity to the experimental monomer structure were searched for using TM‐align.^43^ Among the top 100 entries, up to 10 proteins that are in the desired oligomer state were selected as templates as described above. Based on the target‐template alignments output by TM‐align, models were built using MODELLER, and the lowest energy model was selected as described above. (3) *Detection based on the experimental oligomer structure*: A similar procedure to those described above was applied. Although this time, the best templates were identified by searching for proteins with the highest structural similarity to the target oligomer structure. The search was performed using the multimeric structure alignment tool MM‐align.^44^ For computational efficiency, MM‐align was applied only to the 100 proteins with the highest monomer structure similarity to the target. Models were built using MODELLER based on the alignment output by MM‐align.

## RESULTS

This section is divided into three parts. The first part presents the prediction results for the 25 individual targets for which the docking and scoring experiments were conducted. In the second part, we present an overview of the results across targets and across predictor and scorer groups, respectively. In the third part, we review the accuracy of the models of individual subunits in the predicted oligomers, and how this accuracy influences the performance of docking procedures.

### Prediction results for individual targets

#### Easy homodimer targets: T69, T75, T80, T82, T84, T85, T87, T90, T91, T92, T93, T94

The 12 targets in this category comprised some of the largest subunits of the entire evaluated target set, with sizes ranging between 176 and 456 residues. Four of the targets were multi‐domain proteins (T85, T87, T90, and T93), and one (T82) was an intertwined dimer.

In the following, we present examples of the performance achieved for this category of targets. Detailed results for all the targets of Round 30 can be found in the Supporting Information Table S2, and on the CAPRI website (URL: http://www.ebi.ac.uk/msd-srv/capri/).

**Figure 2:**
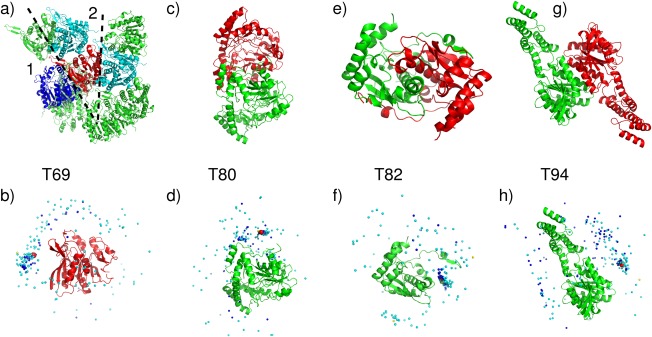
Target structures and prediction results for easy dimer targets. **T69 (T0764),** a Putative esterase (BDI_1566) from *Parabacteroides distasonis*, PDB code 4Q34. (**a**) Target structure, with highlighted interfaces (1,2). (**b**) Global docking prediction results displaying one subunit in cartoon representation, with the center of mass of the second subunit in the target (red sphere), and in docking solutions submitted by CAPRI predictors (light blue spheres), CAPRI scorers (dark blue spheres), and CASP predictors (yellow spheres). **T80 (T0801)**, a sugar aminotransferase WecE from Escherichia coli K‐12, PDB code 4PIW. (**c**) Target structure. (**d**) Global docking prediction results by different predictor groups (see legend (b) for detail). **T82 (T0805)** Nitroreductase (structures unreleased). (**e**) Target structure. (**f**) Global docking prediction results by different predictor groups. **T94 (T0852),** uncharacterized protein Coch_1243 from *Capnocytophaga ochracea* DSM 7271, PDB code 4W9R. (**g**) Target structure. (**h**) Global docking prediction results by different predictor groups.

An illustrative example of the average performance obtained for this category of targets is that obtained for target **T69 (T0764)**: a 341‐residue putative esterase (BDI_1566) from *Parabacteroides distasonis*. The submitted models for this target were evaluated against two interfaces in the crystal structure of this protein, generated by applying the crystallographic symmetry operations listed in the Supporting Information Table S1, and depicted in Figure 2(a): one large interface (2415 Å^2^) and a smaller interface (622 Å^2^). Good prediction results were obtained only for interface 1. Twenty‐eight CAPRI predictor groups submitted a total of 266 models for this homodimer. Of these, 30 were of acceptable quality and 57 were of medium quality. Twelve predictor groups and three docking servers submitted at least one model of acceptable quality or better. Among those, nine groups and one server (CLUSPRO) submitted at least 1 medium quality model. The best performance (10 medium quality models) was obtained by the groups of Seok, Lee and Guerois, followed closely by the groups of Zou, Shen, and Eisenstein (see Supporting Information Table S2 for the complete ranking)

The best model for this target, obtained by Guerois, had an *f(nat)* value of 49%, and *L‐rms* and *I‐rms* values of 2.88 and 2.12 Å, respectively (Supporting Information Table S4).

Six groups, registered with CASP, submitted in total 12 models for this target, comprising one acceptable model by the group of Umeyama and one medium quality model by the Baker group. The global landscape of all the predicted models by the different groups is outlined in Figure 2(b).

An even better performance was achieved by the CAPRI scoring experiment (Supporting Information Table S2). Of the 14 groups participating in this experiment, 12 submitted at least two models of medium quality. The best performance was achieved by Kihara (10 medium quality models), closely followed by Zou and Grudinin, with eight and five medium quality models, respectively. As already observed in previous CAPRI evaluations the best performers in the docking calculations were not necessarily performing as well in the scoring experiment, and thus not singling out even their own best models from the uploaded anonymized set of predicted complexes, highlighting yet again the distinct nature of the docking and scoring procedures.

An important factor in the successful predictions was the overall good accuracy of the 3D models used by predictors in the docking calculations (see Fig. 6 and CAPRI website for detailed values). The best models had an LGA_S score of ∼85 (backbone rmsd of ∼3.9 Å), and only a few models had LGA_S scores lower than 40 (backbone rmsd > 10 Å) (values for all models are available on the CAPRI website**)**. The accuracy of the 3D models across targets and its influence on the predictions will be discussed in a dedicated section below.

Very good predictions were obtained for **T82 (T0805)**, the nitro‐reductase rv3368, a significantly intertwined dimer with unstructured arms reaching out to the neighboring subunit and a subunit interface area of 3250 Å^2^ [Fig. 2(e,f)]. The majority of the models of the individual subunits were quite accurate with LGA_S values of 60–85 (backbone rmsd <5 Å) (see CAPRI website). As many as 54 medium quality models and 17 acceptable models were submitted by CAPRI participants, 99 models of acceptable quality or better were submitted by CAPRI scorer groups, and 11 acceptable models or better were submitted by three CASP groups (Supporting Information Table S2). The high success rate for both complex predictions and subunit modeling stems from the fact that most predictors made good use of known structures of related homodimers in the PDB in which the intertwining mode was well conserved. These known dimer structures were mainly used in templates for modeling the target dimer (template‐based docking).

Very similar participation, number of submitted models and performance, was featured in docking predictions for the other targets in this category (see Supporting Information Tables S2 and S3). The models of individual subunits were also of similar accuracy or higher.

Excellent performance was obtained for targets **T80 (T0819)** and **T93 (T0851)** with >100 correct models of which ∼70 were of medium quality, followed by targets **T90 (T0843)** and **T91 (T0847)**, for which >100 correct models, comprising ∼40 medium quality ones‐ were submitted. These targets featured subunits sizes of 176–456 residues.

**T80 (T0801)** was the sugar aminotransferase WecE from E.coli K‐12, with 376 residues per subunit. Submitted models were evaluated against one interface (1960 Å^2^) between the two subunits of the crystal asymmetric unit [Fig. 2(c)]. A total of 27 CAPRI predictor groups submitted 105 models of acceptable quality or better. The majority of these (71 models) were of medium quality. 12 CAPRI groups participated in the scoring experiment and submitted 120 models, of which about half (51) were of medium quality and 14 were acceptable models. Six CASP participants submitted 11 medium quality models, and two models of acceptable quality. The top ranking CAPRI predictor groups for this target were those of Sali, Guerois, and Eisenstein who submitted 10 medium quality models each. These three groups were closely followed by the groups of Seok, Zou, Shen and Lee, each of whom predicted at least five medium quality models. Each of the three participating servers, HADDOCK, GRAMM‐X, and CLUSPRO, submitted at least one acceptable model. The best performers from among the scorer groups were those of Zou and Huang with 10 medium quality models each, followed by Gray, Kihara and Weng with at least 5 medium quality models, and by Fernandez‐Recio and Bates with four medium quality models. The global landscape of the predictions for this target is shown in Figure 2(d).

The subunit models for this target were of very high quality, with the best models featuring a LGA_S score of ∼95 and a backbone rmsd of 1.3 Å. The quality of the best models for targets T90 and T91 for which a similarly high performance was achieved was only somewhat lower, with LGA_S values of 70–88 and backbone rmsd of 2.0–5.0 Å.

Interestingly, **T91 (T0847),** the human Bj‐Tsa‐9, was predicted to be a dimer by PISA, but assigned as a monomer by the authors. The good docking performance for this target and the fact that the dimer interface (1320 Å^2^) is within the range expected for proteins of this size (176 residues),^45^ suggests that this protein forms a dimer.

A somewhat lower performance was achieved for **T92 (T0849**) the glutathione S‐transferase domain from *Haliangium ochraceum*), and for **T94 (T0852)**, an uncharacterized 2‐domain protein (putative esterase according to Pfam) Coch_1243 from *Capnocytophaga ochracea*. A total of 98 acceptable models were submitted for T92, of which only 12 were of medium quality, but the models were contributed by a large fraction of the participating groups (17 out of 23). On the other hand, the scorer performance was very good with 68 acceptable models of which almost half (33) were of medium quality. These models were contributed across most scorer groups (10 out of 11). CASP participants achieved a particularly good performance. Of the 23 models submitted by CASP groups, 17 were of acceptable quality or better, and those were contributed by six of the seven participating groups. The accuracy of the subunit models was in general lower, with LGA_S ∼70 and rmsd ∼7 Å for the best models, and LGA_S values of 50 – 60 for most other models.

In T94, predicted complexes were assessed only against the largest interface (1190 Å^2^), formed between large domains of the adjacent subunits, as the second largest interface was much smaller (620 Å^2^). In total, 97 acceptable homodimer models only, were contributed for this target: 58 models by CAPRI predictors, 37 by CAPRI scorers, and 2 by CASP groups [see Supplementary Table S2, and Fig. 2(g,h) for a pictorial summary]. The lower accuracy of the subunit models for this target (LGA_S score ∼58 and rmsd >6 Å, for the best model) may have limited the accuracy of the modeled complexes, without however compromising the task of achieving correct solutions.

#### Difficult or problematic homodimer targets: T68, T72, T77, T79, T86, T88

This category comprises 6 targets, representing particular challenges to docking calculations for reason inherent to the proteins involved, or targets for which the oligomeric state was probably assigned incorrectly at the time of the experiment.

With the exception of T72, targets in this category are much smaller proteins, than those of the easy dimer targets (Table 1). In three of the targets (T68, T79, T86) the largest interface area between subunits in the crystal is small (470–860 Å^2^) and their oligomeric state assignments were often ambiguous. In the following, we comment on the insights gained from the results obtained for several of these targets.

**Figure 3:**
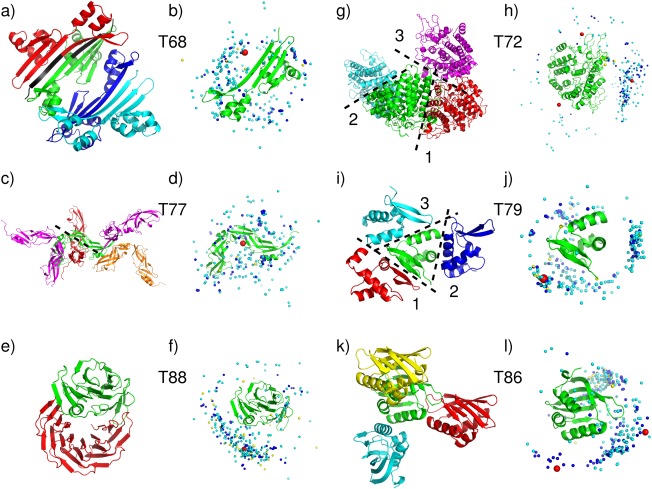
Target structures and prediction results for difficult or problematic dimer targets. **T68 (T0759)**, Plectin 1 and 2 Repeats of the Human Periplakin, PDB code 4Q28. (**a**) Target structure in cartoon representation, displaying 4 subunits in the crystal. The His‐Tag sequence, highlighted in black, mediates contacts at the largest interface. (**b**) Global docking prediction results displaying one subunit in cartoon representation, with the center of mass of the second subunit in the target (red sphere), and in docking solutions submitted by CAPRI predictors (light blue spheres), CAPRI scorers (dark blue spheres), and CASP predictors (yellow spheres). **T77 (T0780),** conserved hypothetical protein (SP_1560), *Streptococcus pneumoniae TIGR4* PDB code 4QDY. (**c**) Target structure, highlighting the assessed interface (dashed line). (**d**) Global docking prediction results by different predictor groups (see legend (b) for detail). **T88 (T0825)**, synthetic wrap five protein (structure unreleased). (**e**) Target structure. (**f**) Global docking prediction results by different predictor groups. **T72 (T0772)**, SusD homolog (BT2259) from *Bacteroides thetaiotaomicron* VPI‐5482, PDB code 4Q69. (**g**) Target structure, highlighting the three assessed interfaces. (**h**) Global docking prediction results for the three interfaces, by different predictor groups. **T79 (T0792)**, OSKAR‐N, PDB code 5a49. (**i**) Target structure, highlighting the three assessed interfaces. (**j**) Global docking prediction results for the three interfaces by different predictor groups. **T86 (T0815)** Putative polyketide cyclase (protein SMa1630) from *Sinorhizobium meliloti*, PDB code 4U13. (**k**) Target structure, showing three interfaces. (**l**) Global docking prediction results for the two interfaces by different predictor groups (the interface with the yellow monomer was not assessed).

No acceptable homodimer models were contributed by CAPRI or CASP groups for targets T68, T77 and T88. The main problem with **T68 (T0759)**, the plectin 1 and 2 repeats of the Human Periplakin, was that the crystal structure contains an artificial N‐terminal peptide representing the His‐tag (MGHHHHHHS…) that was used for protein purification. The N‐terminal segments of neighboring subunits, which contain the artificial peptide, associate to form the largest interface between the subunits in the crystal (1150 Å^2^) [Fig. 3(a)]. Submitted model were assessed against this interface and the second largest interface (860Å^2^), but not against the 2 much smaller interfaces (240 and 160 Å^2^).

Most predictor groups (from both CASP and CAPRI) carried out docking calculations without the His‐tag, which they assumed was irrelevant to dimer formation *in‐vivo*. They were therefore unable to obtain docking solutions that were sufficiently close to the largest interface of the target [Fig. 3(b)]. As well, no acceptable solutions were obtained for second largest interfaces, indicating that it too was unlikely to represent a stable homodimer.

The quality of the subunit models was also lower than for many other targets (the best model had an LGA_S score of ∼57), as most groups ignored the His‐Tag in building the models as well (see Fig. 6 and CAPRI website for details). Considering that the His‐Tag containing peptide contributes significantly to the largest subunit interface, the protein is likely a monomer in absence of the artificial peptide. This is in fact the authors' assignment in the corresponding PDB entry (4Q28), and in retrospect this target should not have been considered for the CAPRI docking experiments.

Different factors contributed to the failure of producing acceptable docking solution for **T77 (T0780)**, the conserved hypothetical protein (SP‐1560), from *Streptococcus pneumonia* TGR4 [Fig. 3(c,d)]. The protein consists of two YbbR‐like structural domains (according to Pfam) arranged in a crescent‐like shape. The domains adopt rather twisted β‐sheet conformations with extensive stretches of coil, and are connected by a single polypeptide segment, suggesting that the protein displays an appreciable degree of flexibility both within and between the domains. Probably as a consequence of this flexibility, the structures of most templates identified by predictor groups (which approximated only one domain), were not close enough to that of the target (Supporting Information Table S5). As a result, the subunit models were generally quite poor, with the best model featuring an LGS‐A score of only ∼40 (rmsd ∼7 Å). Although the largest interface of the target is of a respectable size (1600 Å^2^) and involves intermolecular contacts between one of the domains only, the docking calculations were unable to identify it. The best docking model was incorrect as it displayed an *L‐rms* ∼19 Å, and an *I‐rms* ∼10 Å (see Supporting Information Table S4).

A very different issue plagued the docking prediction of **T88 (T0825)**, the wrap5 protein. The information given to predictors was that the protein is a synthetic construct built from 5 sequence repeats, and is similar to 2YMU (a highly repetitive propeller structure). It was furthermore stated that the polypeptide has been mildly proteolyzed, yielding two slightly different subunits, in which the N‐terminus of the first repeat was truncated to different extent, and that therefore the dimer forms in a non‐trivial way. Predictors were given the amino acid sequence of the two alternatively truncated polypeptides.

It turned out that the longer of the two chains, with the nearly intact first repeat forms the expected 5‐blade β‐propeller fold, whereas the chain with the severely truncated first repeat forms only four of the blades, with the remainder of the first repeat forming an α‐helical segment that contacts the first repeat [Fig. 3(e)].

Both CAPRI and CASP predictor groups were quite successful in building very accurate models for the less truncated subunit (rmsd < 0.5 Å, LGA_S ∼90). But subunit models for the more truncated subunit were much poorer (rmsd 6.5–10 Å), and since the helical region of the shorter subunit contributes significantly to the dimer interface, whose total area is not very large (∼1300 Å^2^), no acceptable docking solutions were obtained [Fig. 3(e,f)].

For the other three targets in this category, T72, T79, and T86, the homodimer prediction performance remained rather poor, with only very few acceptable models submitted. The main issue with **T79 (T0792)**, the OSKAR‐N protein, and **T86 (T0815)**, the polyketide Cyclase from *Sinorhizobium meliloti*, was likely their very small subunit interface (Table 1). T79 was predicted by PISA to be a dimer, but the area of its largest subunit interface is only 680 Å^2^. T86, predicted to be dimeric by both PISA and the authors (as stated in the PDB entry, 4U13), has even smaller size subunit interfaces with the largest one burying no >470 Å^2^. In both cases these interfaces are much smaller than the average size required in order to stabilize weak homodimers.^46^ It is therefore likely that these two proteins are in fact monomeric at physiological concentrations. Furthermore, T79 and T86 are quite small proteins (80 residues for T79, and 100 residues for T86), and it is not uncommon that proteins of this size cannot form large enough interfaces unless they are intertwined.^47^

This notwithstanding, a few acceptable homodimer models were contributed for all three assessed interfaces (interfaces 1,2,3) of T79 (Supporting Information Table S2).

Among predictor groups, 17 acceptable docking solutions (of which five were medium quality models) were obtained for the largest interface (interface 1). Twelve acceptable solutions, of which one medium quality one, were obtained for the second smaller interface (440 Å^2^), and no acceptable quality solutions were obtained for the third assessed interface (400 Å^2^) [see Fig. 3(i,j) for an overview of the prediction results]. Seven CAPRI predictor groups, 1 CASP group and one server (GRAMM‐X) contributed the correct models for interface 1, and seven CAPRI groups submitted acceptable models for interface 2.

Interestingly scorers did less well than predictors for interface 1, but better for interface 2, and two scorer groups submitted two acceptable models for interface 3, whereas none were submitted by predictor groups.

Overall, the models for the T79 subunit were quite accurate, with the best model having and LGA_S score of ∼89 and rmsd ∼1.9 Å.

Not too surprisingly, the dimer prediction performance for T86 was significantly poorer, with only three acceptable models submitted by CAPRI predictors (Ritchie and Negi) for the largest interface (470 Å^2^). Scorers identified five acceptable models for interface 1 (Fernandez‐Recio and Gray), and two acceptable (or better) models for interface 2 (Seok and Kihara). None of the 19 models submitted by the seven CASP groups were correct [Fig. 3(k,l) for a pictorial summary].

Different problems likely led to the weak prediction performance for Target **T72 (T0770)**, the SusD homolog (BT2259) from *Bacteroides Thetaiotaomicron*. While the largest subunit interface is of near average size (1120 Å^2^), the interface itself is poorly packed and patchy, an indication that it may not represent a specific association. Not too surprisingly, therefore, this led to a poor prediction performance. Overall only three models of acceptable quality were submitted by CAPRI dockers, namely by the HADDOCK and SWARMDOCK servers, and the Guerois group, each contributing 1 such model. The best of these models (contributed by Guerois) had *f(nat*) ∼29% and *L‐rms* and *I‐rms* values of 8.85 and 3.57 Å, respectively. Seven acceptable models were submitted by scorers. Bonvin contributed two models, and the groups of Huang, Grudinin, Gray, Weng and Fernandez‐Recio, respectively, submitted one model. The best quality models had *f(nat)* ∼18%, and *L‐rms* and *I‐rms* values of ∼7.29 and 4.28 Å, respectively. No acceptable models were submitted by CASP participants. The target structure and the distribution of the all the docking solutions are depicted in Figure 3(g,h).

The accuracy of the subunit models for T72 was reasonable, with the best models having a LGA_S score of ∼70 (backbone rmsd ∼3.8 Å). The three successful CAPRI predictor groups (HADDOCK, SWARMDOCK and Guerois) all had somewhat lower quality subunit models with LGA_S scores in the range of 55 – 67.

#### Targets assigned as tetramers: T70, T71, T73, T74, T78

Five targets were assigned as tetramers at the time of the prediction experiment. As described in Assessment Procedure and Criteria, models for tetramer targets were assessed by systematically comparing all the interfaces in each model to all the relevant interfaces in the target, and selecting the best‐predicted interfaces. Most predictor groups used a two‐step approach to build their models. First they derived the model of the most likely dimer, and then docked the dimers to one another. Some groups imposed symmetry restraints as part of the docking procedures, or combined this approach with the two‐step procedure.

In three of the targets (T70, T71, T74) predictors faced the problem that all the pair‐wise subunit interfaces were quite small (440–720 Å^2^), making it difficult to identify stable dimers to initiate the assembly procedure.

**Figure 4:**
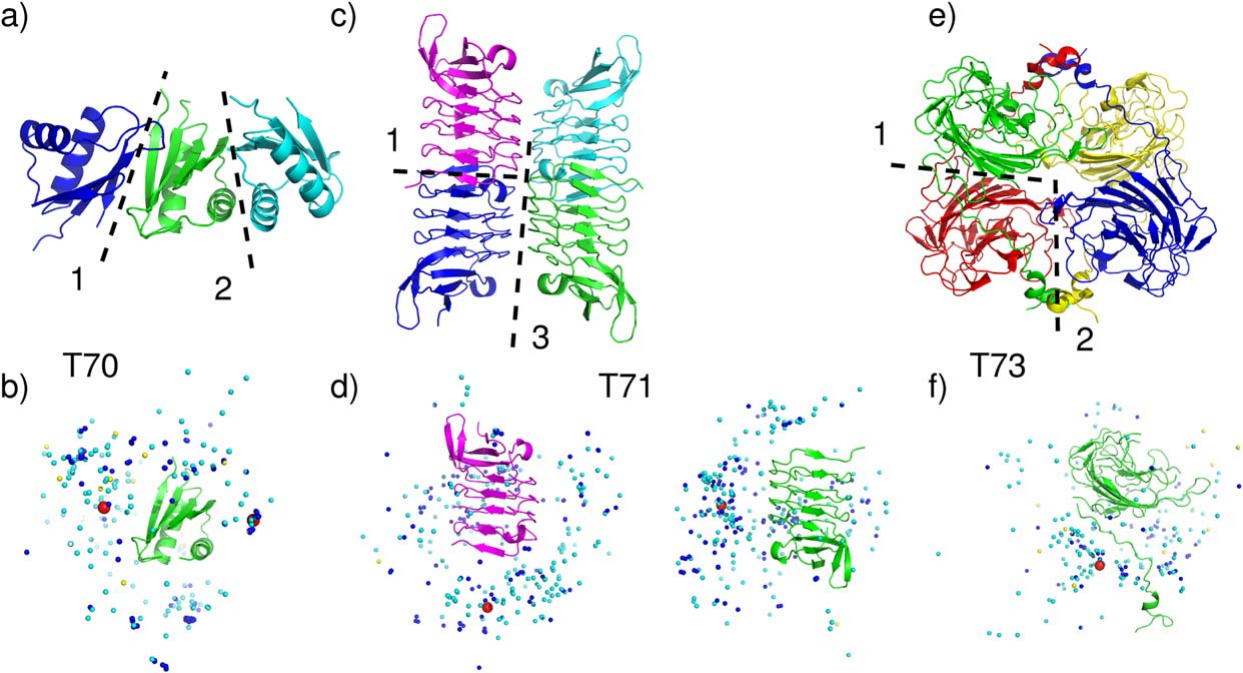
Target structures and prediction results for tetrameric targets. **T70 (T0765)**, Modulator protein MzrA (KPN_03524) from *Klebsiella pneumoniae* subspecies. (**a**) Target structure in cartoon representation, highlighting the two assessed interfaces (dashed lines). (**b**) Global docking prediction results displaying one subunit in cartoon representation, with the center of mass of the second subunit in the target (red spheres), and in docking solutions submitted by CAPRI predictors (light blue spheres), CAPRI scorers (dark blue spheres), and CASP predictors (yellow spheres). **T71 (T0768)** Leucine‐rich repeat protein (BACCAP_00569) from *Bacteroides capillosu*, PDB code 4QJU. (**c**) Target structure in cartoon representation, highlighting the two relevant interfaces (interfaces 1 and 3) (dashed lines). (**d**) Global docking prediction results for the assessed interfaces by different predictor groups (monomer color corresponding to (c), that is, the red spheres represent the same, blue, monomer). **T73 (T0772),** Putative glycosyl hydrolase, PDB code 4QHZ. (**e**) Target structure in cartoon representation, highlighting the two assessed interfaces (interface 1 and 2) (dashed lines). (**f**) Global docking prediction results for the assessed interfaces by different predictor groups.

**T70 (T0765)**, the modulator protein MzrA from *Klebsiella Pneumoniae* Sub Species, was assigned as a tetramer at the time of the predictions, but is listed as a dimer (predicted by PISA and assigned by the authors) in the PDB entry (4PWU). Only two of its interfaces in the crystal bury an area exceeding 400 Å^2^ [Fig. 4(a)]. The assembly built by propagating these two interfaces appears to form an extensive layered arrangement across unit cells in the crystal, rather than a closed tetramer.

Interestingly, acceptable or better models were submitted only for the smaller interface (475 Å^2^) (Supporting Information Table S2). CAPRI predictors submitted 37 acceptable models, of which 27 were of medium quality, and scorers submitted 27 acceptable models (including 21 medium quality ones) [Fig. 4(b)]. Indeed no acceptable models were submitted for the largest interface (560 Å^2^), which is assigned as the dimer interface in the PDB entry for this protein.

The failure to model a higher order oligomer for this target was not due to the quality of the subunit models as the latter was quite high (see Fig. 6 and CAPRI website**)**, and is probably rooted in the pattern of contacts made by the protein in the crystal, which suggest that this target is likely a weak dimer. Considering that all the acceptable docking models involve a different interface than that assigned in the corresponding PDB entry, it is furthermore possible that the interface identified in these solutions is in fact the correct one. But given the very small size of either interface, the protein could also be monomeric.

A similar situation was encountered with **T74 (T0774)**, a hypothetical protein from *Bacteroides vulgatus*. Here too the target was assigned as a tetramer by PISA at the time of the predictions, but is listed as a monomer by the authors in the PDB entry (4QB7). Associating the subunits according to the two largest interfaces (520 and 490 Å^2^), also produced an open‐ended assembly rather than a closed tetramer, and this time no acceptable solutions were produced for either interface, strongly suggesting that the protein is monomeric as specified by the authors. It is noteworthy that the subunit models for this target were particularly poor (LGA_S values ∼40, and rmsd ∼7 Å), which could also have hampered identifying some of the binding interfaces.

**T71 (T0768)**, the leucine‐rich repeat protein from *bacteroides capillosus*, was a difficult case for other reasons. Subunit contacts in the crystal are mediated through three different interfaces, ranging in size from 470 Å^2^ to 720 Å^2^. A closed tetrameric assembly can be built by combining interfaces 1 and 3, associating the dimer formed by subunits A and B with the equivalent dimer of subunits C and D, as shown in Figure 4(c). Interfaces 1 and 3 were also those for which some acceptable predictions were submitted. One acceptable model was contributed for the largest interface, by the GRAMM‐X, an automatic server. Eleven acceptable models were submitted for the third interface (470 Å^2^) by 4 CAPRI predictor groups, and six acceptable models were submitted by four CAPRI scorer groups. All the models submitted by a single CASP group were wrong. No group succeeded in building the tetramer that comprises the correct models for interfaces 1 and 3 at the same time. Some models looked promising, but when superimposing equivalent subunits (in the model vs. the target) the neighboring subunit of the model (the one across the incorrectly predicted interface) had its position significantly shifted relative to that in the target, resulting in an incorrect structure of the tetrameric assembly.

The remaining two targets, **T73 (T0772)**, a putative glycosyl hydrolase from *Parabacteroides distaspnos,* and **T78 (T0786),** a hypothetical protein from *Bacillus cereus*, were genuine tetramers assigned as such by both PISA and the authors. Both targets are proteins of similar size (∼260 residues) adopting an assembly with classical D_2_ symmetry, which comprises two interfaces, a sizable one (>1000 Å^2^) and a smaller one. But the main bottleneck for both targets was that their larger interface was intertwined. Available templates did not seem to capture the intertwined associations, as witnessed from the overall poorer models derived for the individual subunits. For both targets, the best models had an LGA_S score ∼50 and a backbone rmsd of ∼5–10 Å. For T73, a total of only nine acceptable models were submitted by the CAPRI predictor groups of LZERD, Zou and Kihara for the largest interface, and two acceptable models were submitted by the Lee group for the second interface. None of the predicted tetramer models simultaneously captured both interfaces, as illustrated in Figure 4(e,f). For T78, no acceptable solutions were submitted by any of the participating groups, but the subunit models were only marginally more accurate than those of T73.

The conclusions to be reached from the analysis of these five targets are twofold. One is that the oligomeric state assignment for higher order assemblies such as tetramers is more error prone than that of dimer versus monomers. Tetramers often involve smaller interfaces between subunits, especially those formed between individual proteins when two dimers associate, and therefore predictions on the basis of pair‐wise crystal contacts such as those by PISA become unreliable. Independent experimental evidence is therefore required to validate the existence of a higher order assembly. The second conclusion to be drawn is that the prediction of higher order assembly by docking procedures remains a challenge. Acceptable models derived for the largest dimer interface are probably not accurate enough to enable the identification of stable association modes between two modeled dimers. This indicates in turn that the propagation of errors is the problem that currently hampers the modeling of higher order assemblies from the structures of its components in absence of additional experimental information.

#### Heterocomplex targets: T81, T89

**T81 (T0797/T0798)** and **T89 (T0840/T0841)** were the only two *bona‐fide* heterocomplex targets in Round 30. T81 is the complex between the cGMP‐dependent protein Kinase II leucine zipper (44 residues) and the Rab11b protein (198 residues) (PDB code 4OJK). T89 is the complex between the much larger RON receptor tyrosine kinase subunit (669 residues) and the macrophage stimulating protein subunit (MSP) (253 residues).

**Figure 5:**
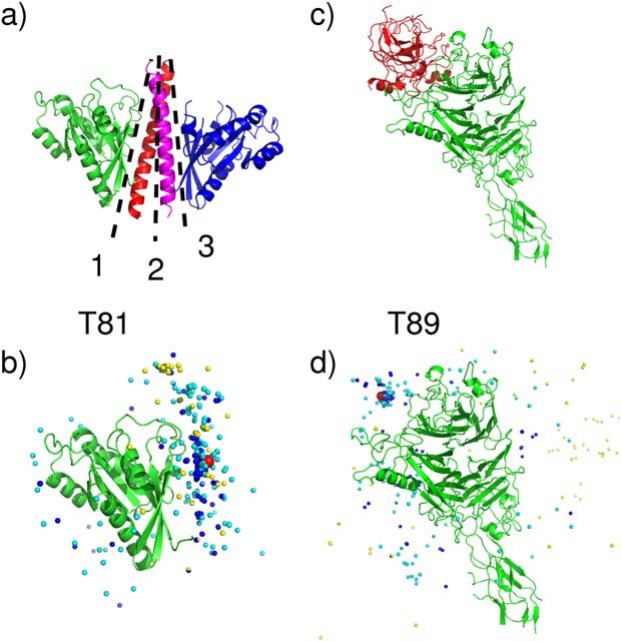
Target structures and prediction results for heterocomplex targets. **T81 (T0797/T0798)**, cGMP‐dependent Protein Kinase II Leucin Zipper and Rab11b Protein Complex, PDB code 4OJK. (**a**) Target structure in cartoon representation, highlighting the interface of the leucine zipper dimer (2), and the two equivalent interfaces (1,3), between the zipper dimer and the two Rab11b proteins (dashed lines). (**b**) Global docking prediction results displaying one of the Rab11b subunits in cartoon representation, with the center of mass of the leucine zipper dimer in the target (red sphere), and in docking solutions submitted by CAPRI predictors (light blue spheres), CAPRI scorers (dark blue spheres), and CASP predictors (yellow spheres). **T89 (T0840/T0841)**, complex of the RON receptor tyrosine kinase subunit and the macrophage stimulating protein subunit (MSP) (structure not released). (**c**) Target structure in cartoon representation. (**d**) Global docking prediction results displaying the RON receptor kinas subunit, in cartoon representations, and the center of mass of the MCP proteins in the target and in docking solutions submitted by different predictor groups.

The crystal structure of T81 features two Rab11b proteins binding on opposite sides of the centrally located leucine zipper, in a quasi‐symmetric arrangement, which likely represents the stoichiometry of the biological unit [Fig. 5(a)]. A total of 3 interfaces were evaluated for this targets: Interface 1 (chains C:A, leucine zipper helix 1/one copy of the Rab11b protein), Interface 2 (C:D, leucine zipper helix 1/helix 2), interface 3 (equivalent to interface 1). The two Rab11b/zipper helix interfaces were not exactly identical (780 Å^2^ for interface 1 and 630 Å^2^ for interface 2). The interface between the helices of the leucine zipper was somewhat larger (780 Å^2^). Overall, the interface area of a single copy of the Rab11b protein binding to the leucine zipper dimer measures 1070 Å^2^.

Consolidating correct predictions for the equivalent interfaces (Interfaces 1 and 3), the prediction performance for this complex as a whole was disappointing. Only 12 correct models were submitted by the 7 CAPRI predictor groups of Guerois, Seok, Huang, Vajda/Kozakov, SWARMDOCK, CLUSPRO (a server) and Bates. Five of those (submitted by Guerois, Seok and Huang) were of medium quality. The performance of CAPRI scorers was better, with 54 correct models of which 16 of medium quality. All 11 scorer groups contributed these models, and the best scorer performance was achieved by the groups of Bates, followed by those of LZERD, Oliva, Huang, Fernandez‐Recio and Seok. The prediction landscape for this target is shown in Figure 5(b).

T89, the RON receptor kinase subunit complex with MSP, was a simpler target, given the clear, binary character of this heterocomplex. But the large size of the receptor subunit, and the relatively small interface it formed with MSP, represented a challenge for the docking calculations. The prediction performance for this complex was quite good overall, with a total of 87 correct models submitted by predictors, representing 41% of all submitted predictor models. Unlike for many other targets of this round, scorers did only marginally better, with 42% of correct models. CASP groups were specifically invited to submit models for this target, and 55 groups did, nearly ten times more than for other targets in this round. But their performance was much poorer than that of CAPRI groups. Only 23 models out of the 223 submitted by CASP groups (10%) were correct, and 6 of these were medium accuracy models.

The best performance among CAPRI predictor groups was by the HADDOCK server, followed by the groups of Vakser, Seok, Guerois, Grudinin, Lee, Huang and Tomii (see Supporting Information Table S2). A pictorial summary of the prediction performance for this target is provided in Figure 5(c,d).

### Results across targets and groups

#### Across target performance of CAPRI docking predictions

**Figure 6:**
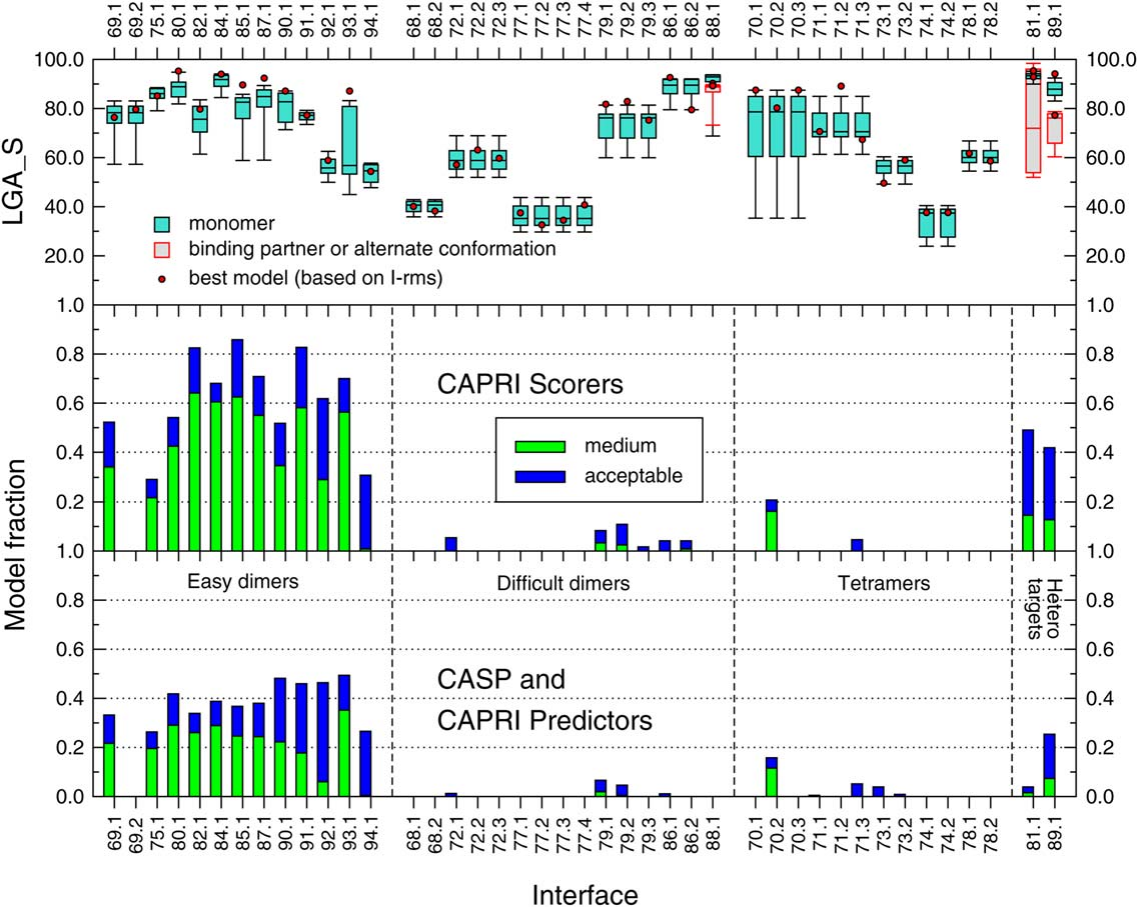
Pictorial summary of the prediction results per assessed interface of the targets in CAPRI Round 30. The lower panel depicts the fraction of models of acceptable and medium quality respectively, submitted by CAPRI and CASP predictor groups, for the 42 assessed interfaces in all 25 targets (listed along the horizontal axis). The digit following the CAPRI target number represents the assessed interface. The symmetry transformation corresponding to the assessed interfaces in each target are listed in the Supporting Information Table S1. The fraction of correct models is shown separately for the four main target categories: Easy dimer targets, difficult (or problematic) dimer targets, tetrameric targets, and heterocomplex targets. The middle panel displays the same data for models submitted for the same interfaces by CAPRI scorer groups. The top panel shows box plots of the LGA_S score values of the subunits in submitted models for the targets listed along the horizontal axis. The LGA_S score is one of the CASP measures of the accuracy of the predicted 3D structure of a protein.^35^ The red dots represent the LGA_S score of the subunit structure of the best quality homo or heterocomplex model submitted for each target. The best quality model is defined as the one with the lowest *I‐rms* (see **Fig**. 1 for details).

Results of the docking and scoring predictions for the 25 assessed targets of Round 30, obtained by all groups that submitted models for at least one target, are summarized in Figure 6 and in the Supporting Information Table S3. For a full account of the results for this Round the reader is referred to the CAPRI web site (http://www.ebi.ac.uk/msd-srv/capri/).

The results summarized in Figure 6 show clearly that the prediction performance varies significantly for targets in the four different categories. As expected, the performance is significantly better for the 12 dimer targets in the “easy” category, than for those in the other categories. For 10 of the 12 “easy” targets, at least 30% of the submitted models per target are of acceptable quality or better, and for most of these (eight out of 10), at least 20% of the models are of medium quality. The accuracy of the subunit models (top panel, Fig. 6) is rather good for most of these targets. With the exception of T93, for which the quality of the subunits models spans a wide range (LGA_S ∼40–80), the models of the remaining 11 targets achieve high LGA_S scores with averages of 80 or above.

The two less well‐predicted targets in this category are T92 and T94, probably due to the lower quality of the subunit models (average LGA_S < 60) (top panel, Fig. 6).

The docking prediction performance is quite poor for the six “difficult or problematic” dimer targets, where a few acceptable models were submitted for only three of the targets (T72, T79, T86), and no acceptable models were submitted for the remaining three targets. This very poor performance was not rooted in the docking or modeling procedures but rather in the targets themselves. In 4 of the targets in this category (T68, T72, T79, T86) the oligomeric state (dimer in this case), often predicted only by PISA, but sometimes also provided by the authors, was likely incorrectly assigned. In T68, the His‐tag used for protein purification and included in the crystallization forms the observed dimer interface, which is therefore most certainly non‐native. In T72 the main problem was its very poorly packed and patchy interface, suggesting that the dimer might be a crystal artifact, whereas in T79 and T86, all the pair‐wise interfaces in the crystal structure were too small for any of them to represent a stable dimer.

The only genuinely difficult dimer targets were T77 and T88. For T77, the subunits of this flexible 2‐domain protein were rather poorly modeled (average LGA_S 30–40), making it difficult to model the “handshake” arrangement of the subunits in the dimer [Fig. 3(c,d)]. In T88, the synthetic wrap5 protein, most predictor groups failed to meet the challenge of correctly modeling the shorter of the two subunits, in turn leading to incorrect solutions for the heterodimer.

As already mentioned, a very poor performance was achieved for the five targets assigned as tetramers at the time of the predictions. This is illustrated at the level of the individual interfaces in these targets (Fig. 6). However, here too the problem was not necessarily rooted in limitations of the docking or modeling procedures. Two of the targets, T70 and T74, seem to have been erroneously assigned as tetramers at the time of the prediction by PISA, as described above. T70 was assigned as a dimer, and T74 as a monomer, by the respective authors in the PDB entry. In agreement with the authors' assignment, no acceptable solutions were identified for any of the interfaces in T74. Somewhat surprisingly, the quality of the subunits models for this target was particularly poor as well (average LGA_S ∼30).

In T70, the docking calculations were able to identify only the smaller of the two interfaces as forming the dimer interface (Fig. 6), but this interface differs from the one assigned by the authors. This result leaves open the possibility that this protein may indeed be a weak dimer, in agreement with the author's assignment, albeit a different dimer than the one that they propose. Thus for both of these seemingly erroneously assigned tetrameters, the docking calculations actually gave the correct answer, which supports the author's subsequent assignments, which were not made available at the time of the prediction experiment.

For the other three tetrameric targets, T71, T73 and T78, the poor interface prediction performance reflects the genuine challenges of modeling higher order oligomers. In T71 the small size of the individual interfaces was likely the reason for the paucity of acceptable dimer models, and those were moreover not accurate enough to enable the correct modeling of the higher order assembly (dimer of dimers). In T73 and T78, the very few acceptable models for interfaces in the former, and the complete failure to model any of the interfaces in the latter (Fig. 6), likely stem from the lower accuracy of the corresponding subunit models (average LGA_S ∼50–60).

The docking prediction performance was better, but not particularly impressive for the two heterocomplex targets T81 and T89, which represent the type of targets that the CAPRI community commonly deals with. For T81 only ∼5% of the submitted models were of acceptable quality or better, whereas for T89 the corresponding model fraction was 40%, similar to that achieved for the easy dimer targets. The poorer performance for T81 can be readily explained by the fact that this target was in fact a hetero tetramer, two copies of the Rab11b protein binding to opposite sides of a leucine zipper, which had to be modeled first.

These results taken together indicate that homology modeling techniques and docking calculations are able to predict rather well the structures of biologically relevant homodimers. In addition we see that the prediction performance for such targets is on average superior than that obtained for heterocomplexes in previous CAPRI rounds, where on average only about 10–15% of the submitted models are correct for any given target (http://onlinelibrary.wiley.com/doi/10.1002/9781118889886.ch4/summary), compared to 25% obtained for the majority of the genuine dimer targets in this Round, including both easy and difficult homodimers. This result is not surprising, as interfaces of homodimers are in general larger and more hydrophobic than those of heterocomplexes,^45^ properties which should make them easier to predict.

Another noteworthy observation is that docking calculations can often help to more reliably assign the protein oligomeric state, especially in cases where available assignments were ambiguous. Such cases were encountered for several of the difficult or problematic targets, and for targets assigned as tetramers. On the other hand, the main challenge in correctly modeling tetramers is to minimize the propagation of errors caused by even small inaccuracies in modeling individual interfaces, which can in turn be exacerbated by inaccurate 3D models of the protein components.

#### Across target performance of CAPRI scoring predictions

As shown in the middle panel of Figure 6, CAPRI scorer groups achieved overall a better prediction performance than predictor groups. The scoring experiment involves no docking calculations, and only requires singling out correct solutions from among the ensemble of models uploaded by groups participating in the docking predictions. Clearly, such solutions cannot be identified if the ensemble of uploaded models contains only incorrect solutions. Therefore no correct scoring solutions were submitted by scorers for targets where no acceptable docking solutions were present within the 100 models uploaded by predictor groups for given target.

However, for targets where at least a few correct docking models were obtained by predictors, scorers were often able to identify a good fraction of these models, as well as other models that were not identified amongst the 10 best models by the groups that submitted them (Fig. 6). This was particularly apparent for the easy dimer targets, where scorers often submitted a significantly higher fraction of acceptable‐or‐better models (>50%) than in the docking experiment, where this fraction rarely exceeded 40%. A similar result was achieved for the heterocomplexes, and was particularly impressive for T81, where nearly half of the submitted models by scorers were correct, compared to only 5% for the docking predictions.

The seemingly superior performance of scorers over dockers has been observed in previous CAPRI assessments^16^, ^19^ where it was attributed in part to the generally poor ranking of models by predictors. Their highest‐ranking models are often not the highest‐quality models, and acceptable or better models can often be found lower down the list and amongst the 100 uploaded models. Another reason is the fact that the search space that scorers have to deal with is orders of magnitude smaller (a few thousands of models), than the search space dockers commonly sample (tens of millions of models). This significantly increases the odds of singling correct solutions in the scoring experiment.

Clearly however, there is more to the scorers' performance than chance alone, particularly in this CAPRI Round, where the main challenge was to model homo‐oligomers. Some groups that have also implemented docking servers had their server perform the docking predictions completely automatically, but carried out the scoring predictions in a manual mode, which still tends to be more robust. In addition, a meta‐analysis of the uploaded models, such as clustering similar docking solutions and selecting and refining solutions from the most populate clusters can also lead to improved performance.

This notwithstanding, the actual scoring functions used by scorer groups must play a crucial role. But this role is currently difficult to quantify in the context of this assessment.

#### Performance across CAPRI and CASP predictors, scorers and servers

The ranking of CAPRI‐CASP11 participants by their prediction performance on the 25 targets of Round 30 is summarized in Table 4. The per‐target ranking and performance of participants can be found in the Supporting Information Tables S2 and S3.

**Table 4:** Participant ranking by Target performanceParticipant

	Participated targets	Performance		
**CAPRI Predictor Ranking**				
Seok	25	15/14**		
Huang	25	16/13**		
Guerois	25	16/12**		
Zou	25	14/11**		
Shen	25	13/11**		
Grudinin	24	11/10**		
Weng	25	13/9**		
Vakser	25	11/9**		
Vajda/Kozakov	24	15/8**		
Fernandez‐Recio	25	11/8**		
Lee	20	10/7**		
Tomii	20	8/6**		
Sali	12	6/4**		
Negi	25	7/3**		
Eisenstein	6	3**		
Bates	25	7/2**		
Kihara	23	7/2**		
Zhou	25	4/2**		
Tovchigrechko	12	3/1**		
Ritchie	8	2/1**		
Fernandez‐Fuentes	14	**1**		
Xiao	11	**1**		
Gong	8	**0**		
Del Carpio	3	**0**		
Wade	2	**0**		
Haliloglu	1	**0**		
**CAPRI SERVER Ranking**				
HADDOCK	25	16/9**		
CLUSPRO	25	16/8**		
SWARMDOCK	25	11/4**		
GRAMM‐X	22	6/1**		
LZERD	25	**3**		
DOCK/PIERR	2	**1**		
**CAPRI Scorer Ranking**				
Bonvin	25	18/14**		
Bates	24	17/13**		
Huang, Seok	25	16/13**		
Zou, Kihara	25	15/12**		
Fernandez‐Recio	25	14/12**		
Weng	25	16/11**		
Oliva	22	14/11**		
Grudinin	25	13/10**		
Gray	17	10/7**		
LZERD	25	6**		
Lee	5	3/2**		
Sali	1	**0**		
**CASP Predictor and Server Ranking**				
Umeyama	19	13/8**		
ROSETTASERVER	13	9/8**		
Dunbrack	12	11/6**		
SEOK_SERVER	22	7/5**		
Luethy	8	5/4**		
Nakamura	12	7/3**		
Baker	8	3**		
Wallner	2	1**		
Skwark, Lee, RAPTOR‐X_Wang, NNS_Lee	1–4	**1**		
*39 participants not listed*	1–5	**0**		

For each target only the best quality solution is counted; in total 25 targets were assessed. Column 2 indicates the number of targets for which predictions were submitted. In Column 3, the numbers without stars indicate models of acceptable quality or better, and the numbers with “**” indicate the number of those models that were of medium quality.

The ranking in Table 4 considers only the best quality model submitted by each group for every target. The ranking in the Supporting Information Table S2 takes into account both the total number of acceptable models, and the number of higher quality models (medium quality ones for this Round, as detailed in the section on assessment criteria). When two groups submitted the same number of acceptable models, the one with more high quality models is ranked higher, and when two groups submitted the same number of high quality models, the group with more acceptable models is ranked higher.

Overall, a total of 11 CAPRI predictor groups submitted correct models for at least 10 targets, and medium quality models for at least seven targets. These groups submitted models for at least 20 of the targets. Among those, the highest‐ranking groups in this Round are Seok, Huang, and Guerois, with correct models for 15 or 16 targets, and medium quality models for 12–14 of these targets. These are followed by Zou, Shen and Grudinin (correct models for 11–14 targets, and medium quality models for 10 or 11 of those). The remaining five highest ranking groups, Weng, Vakser, Vajda/Kozakov, Fernandez‐Recio and Lee, achieve correct predictions for 10–15 targets and medium quality predictions for 7–9 of those. It is noteworthy that two of the three top ranking predictor groups (Seok and Guerois), and at least one other group (Vakser) made heavy use of template‐based modeling, an indication that this approach can be quite effective.

The remaining groups listed in Table 4 were ranked lower, as they corrected predicted between 1 and 8 targets only, and produced only a few medium quality models for these targets. However some of these groups submitted predictions for a smaller number of targets. Their performance can therefore not be fairly compared to that of other groups.

Of the 6 CAPRI automatic docking servers ranked in Table 4, HADDOCK and CLUSPRO rank highest, followed by SWARMDOCK, and GRAMM‐X.

It is interesting to note that two top ranking CAPRI servers submitted correct predictions for 16 targets, just as many as the top ranking predictor groups. But the latter groups still produce more medium accuracy models (>10) than the servers (no more than 9). Thus as already noted in previous CAPRI assessment, some CAPRI servers perform nearly on par with more manual predictions.

Among the CASP predictor and server groups listed in Table 4, the groups of Umeyama and Dunbrack rank highest, and both would rank among the best CAPRI predictor groups as their success rate (fraction of correct over submitted models) was also high. Of the servers, ROSETTASERVER and SEOK_SERVER rank highest, with a performance level similar to SWARMDOCK. Thirty‐nine CASP groups submitting models for 1–5 targets, none of which were correct, are not explicitly listed in the Table.

Lastly, judging also by the best model submitted for each target, CAPRI scorers outperform CAPRI predictors, as already mentioned when analyzing the performance across targets. Highly ranking scorer groups submitted on average correct models for 1–2 more targets than CAPRI predictors, and the number of medium quality models that groups submit for these targets is also somewhat higher.

Of the 13 scorer groups that submitted an accurate model for at least one target, 11 have correctly predicted at least 10 targets and submitted medium quality models for seven of those.

The best performing groups are those of Bonvin, Bates, Huang, Seok, Zou and Kihara, followed closely by four other groups that correctly predicted at least 13 targets, and produced medium quality models for at least 10 of these (Table 4).

### Factors influencing the prediction performance

Unlike in previous CAPRI rounds, Round 30 comprised solely targets where both the 3D structure of the protein subunits and their association modes had to be modeled. Deriving the atomic coordinates of the predicted homo‐oligomers therefore involved a number of steps each requiring the use of specialized software and making strategic choices as to how it should be applied.

As mentioned in Synopsis of the Prediction Methods, the approaches for modeling the subunit structures and generating the oligomer models vary widely amongst predictor groups, and across targets. It is therefore difficult to reliably pinpoint specific factors that contributed or hampered successful predictions. Nonetheless some general trends can be outlined. Even though Round 30 comprised only targets whose subunits could be readily modeled using templates from the PDB, the subunit modeling strategy had an important influence on the final oligomer models. Groups that used several different subunit models for the same target increased their chance of deriving at least an acceptable oligomer model. Such different models were obtained either by using different templates (some groups used as many as five templates for the same target), or by starting from the same template and modifying it by optimizing loop conformations and subjecting it to energy refinements. These optimizations seemed particularly effective when carried out in the context of the oligomers representing the highest‐ranking template‐based or docking models.

As already mentioned, information on oligomeric templates in the PDB was another important element contributing to improve the prediction performance. This information was the main ingredient for two of the best performing groups that heavily relied on template‐based docking. Other groups that performed well used mainly *ab‐initio* docking methods of various origins, but either guided the calculations or filtered the results based on structural information from homologous oligomers.

Other important elements, such as selecting representative members of clusters of docking solutions, and the final scoring functions used to rank models and select those to be submitted, also played a role as already mentioned here and in previous CAPRI reports.^19^

In the following we examine in more detail the impact of two important elements of this joint CASP‐CAPRI experiment. We evaluate the influence of the accuracy of individual subunits models on the oligomer prediction performance, and estimate the extent to which procedures that rely on docking methodology and those that employ specialized template‐based modeling confer an advantage over straightforward homology modeling.

#### Influence of subunit model accuracy

The subunit models used to derive the models of the oligomers were generated either by CAPRI groups, those with more homology modeling expertise, or borrowed from amongst the models submitted by CASP servers, which were made available to CAPRI groups in time for each docking experiment. The subunit structures in models submitted by CAPRI and CASP groups for all 25 targets of Round 30 were assessed using the standard CASP GDT_TS and LGA_S scores, as well as the backbone rmsd of the submitted model versus the target structures. The values of these measures obtained for models submitted by all participants in Round 30 for each target can be found at the CAPRI website together with the assessment results for this Round.

**Figure 7:**
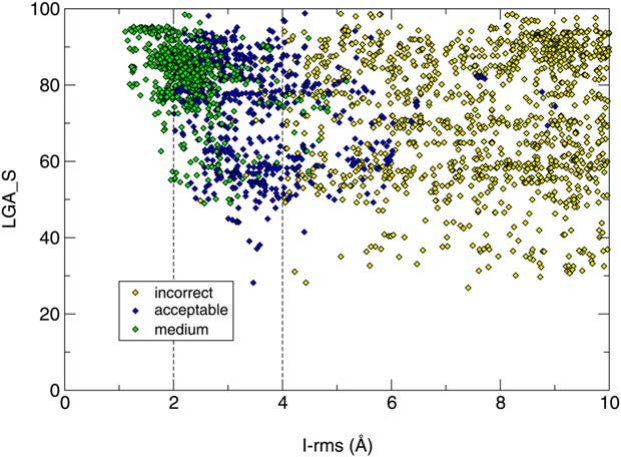
Subunit model accuracy and the quality of predicted complexes in CAPRI Round 30. The CASP LGA_S scores of subunit models in the predicted complexes for the 25 targets in this Round (vertical axis) are plotted as a function of the *I‐rms* values (horizontal axis). Each point in this Figure represents one submitted model, and points are colored according to the quality of the predicted complex, respectively, incorrect (yellow), acceptable (blue) and medium (green) quality (see Table 1 and the text for details).

To gauge the relation between the accuracy of subunit models and the docking prediction performance, the LGA‐S scores of subunit models in the predicted complexes for the 25 targets in this Round are plotted in Figure 7 as a function of the *I‐rms* value. The LGA_S measure was used because it does not depend on the residue numbering along the chain, which may vary at least in a fraction of the models submitted by CAPRI participants. The *I‐rms* measure was used as it represents best the accuracy level of the predicted interface.

Each point in Figure 7 represents one submitted model, and points are colored according to the quality of the predicted complex (incorrect, acceptable and medium quality). The plot clearly shows that medium quality predicted complexes (*I‐rms* values between 1 and 3 Å) tend to be associated with high accuracy subunit models (LGA_S values >80). We also see that predicted complexes of acceptable quality (*I‐rms* values of 2–4 Å) are associated with subunit models that span a wide range in accuracy levels (LGA_S between 30 and 90). This range is comparable to the subunit accuracy range associated with incorrect models of complexes (*I‐rms* >4 Å; see Table 3 for details on how *I‐rms* contributes to rank CAPRI models). Identical trends are observed when plotting the GDT‐TS scores as a function of the *I‐rms* values for the fraction of the models with correct residues numbering (Supporting Information Fig. S2).

That both accurate and inaccurate subunit models are associated with incorrectly modeled complexes is expected. Inaccurate subunit models may indeed prevent the identification of the correct binding mode, and docking calculations may fail to identify the correct binding mode even when the subunit models are sufficiently accurate. It is however noteworthy that complexes classified as incorrect by the CAPRI criteria do not necessarily represent prediction noise, as a recent analysis has shown that residues that contribute to the interaction interfaces are correctly predicted in a significant fraction of these complexes.^48^

Somewhat less expected is the observation (Fig. 7) that in a significant number of cases, acceptable and to a smaller extent also medium quality docking solutions can be identified even with lower accuracy models of the individual subunits. This is an encouraging observation, as it suggests that docking calculations can lead to useful solutions with protein models built by homology, and that these models need not always be of the highest accuracy. What probably matters more for the success of docking predictions is the accuracy with which the binding regions of the individual components of the complex are modeled, rather than the accuracy of the 3D model considered in its entirety.

#### Round 30 predictions versus standard homology modeling

To estimate the extent to which docking methods or template‐based modeling procedures confer an advantage over straightforward homology modeling, the accuracy of the submitted oligomer models for each target was compared to the accuracy of the models build using the best oligomer templates for that target available in the PDB at the time of the prediction. Only dimer targets (and templates) were considered, given the uncertainty of the oligomeric state assignments for some of the tetrameric targets.

Three categories of the best dimeric templates were considered (see Assessment Procedures and Criteria): templates identified on the basis of sequence alignments alone, templates identified by structurally aligning the target and template monomers, and templates identified by structurally aligning the target and template oligomers. Only the sequence‐based template selection reconstitutes the task performed by predictors, to whom only the target sequence was disclosed at the time of the prediction. The resulting templates thus represent the best templates available to predictors during the prediction Round. Obviously, the structurally most similar templates could not be identified by predictors, but are considered here in order to evaluate the advantage, if any, conferred by such templates over those identified on the basis of sequence alignments.

Table 5 lists the best templates from each category identified for all dimeric targets of Round 30 and the corresponding template‐target TM‐scores. These templates represent those with the highest TM‐score among the best 10 templates from each category detected for a given targets. Not too surprisingly targets with more similar templates, those featuring high TM‐scores (≥0.7), are the easy targets, whereas difficult targets are those with poorer templates (lower TM‐scores). Many of the best templates from all three categories were also detected and used by predictor groups (see Supporting Information Table S5), even though these groups only had sequence information to identify them during the prediction round.

**Table 5:** Best available templates detected based on sequence (“Sequence”), experimental monomer structure (“Monomer”), and experimental oligomer structure (“Oligomer”)Target

	Target released	Database released	Best template TM‐score (detected template)		
			Sequence	Monomer	Oligomer
T68	May 01, 2014	April 24, 2014	0.348 (3njd)	0.370 (3fse)	0.370 (3fse)
T69	May 05, 2014	April 24, 2014	0.852 (1qlw)	0.852 (1qlw)	0.852 (1qlw)
T70	May 06, 2014	April 24, 2014	0.639 (2f06)	0.644 (3c1m)	0.652 (3tvi)
T71	May 07, 2014	April 24, 2014	0.509 (2id5)	0.618 (3jur)	0.618 (3jur)
T72	May 08, 2014	April 24, 2014	0.510 (3otn)	0.510 (3otn)	0.510 (3otn)
T73	May 09, 2014	April 24, 2014	a	0.554 (1hql)	0.554 (1hql)
T74	May 12, 2014	April 24, 2014	0.340 (4jrf)	0.340 (4jrf)	0.340 (4jrf)
T75	May 13, 2014	April 24, 2014	0.880 (3rjt)	0.880 (3rjt)	0.880 (3rjt)
T77	May 15, 2014	April 24, 2014	0.393 (2xwx)	0.375 (4iib)	0.375 (4iib)
T78	May 20, 2014	May 17, 2014	0.315 (3c6c)	0.370 (1o0s)	0.403 (2f3o)
T79	May 23, 2014	May 17, 2014	0.440 (2bnl)	0.469 (2xig)	0.471 (2w57)
T80	June 02, 2014	May 17, 2014	0.938 (1mdo)	0.943 (2fnu)	0.943 (2fnu)
T82	June 04, 2014	May 17, 2014	0.846 (4dn2)	0.846 (4dn2)	0.846 (4dn2)
T84	June 09, 2014	May 17, 2014	0.939 (2btm)	0.941 (1b9b)	0.941 (1b9b)
T85	June 10, 2014	May 17, 2014	0.889 (3ggo)	0.889 (3ggo)	0.889 (3ggo)
T86	June 11, 2014	May 17, 2014	0.459 (4h3u)	0.467 (3gzr)	0.470 (3hk4)
T87	June 13, 2014	May 17, 2014	0.922 (3get)	0.922 (3get)	0.922 (3get)
T90	July 03, 2014	June 06, 2014	0.921 (4qgr)	0.927 (2oga)	0.927 (2oga)
T91	July 08, 2014	June 06, 2014	0.750 (4gel)	0.750 (4gel)	0.808 (3hsi)
T92	July 09, 2014	June 06, 2014	0.785 (1tu7)	0.837 (3h1n)	0.837 (3h1n)
T93	July 10, 2014	June 06, 2014	0.896 (4a7p)	0.896 (4a7p)	0.896 (4a7p)
T94	July 11, 2014	June 06, 2014	0.655 (3gff)	0.655 (3gff)	0.655 (3gff)

TM‐score of the templates that have the highest TM‐score among top 10 selected templates for each target and the PDB IDs of the templates are listed.

a No protein with the desired oligomer state was found among the top 100 HHsearch entries.

**Figure 8:**
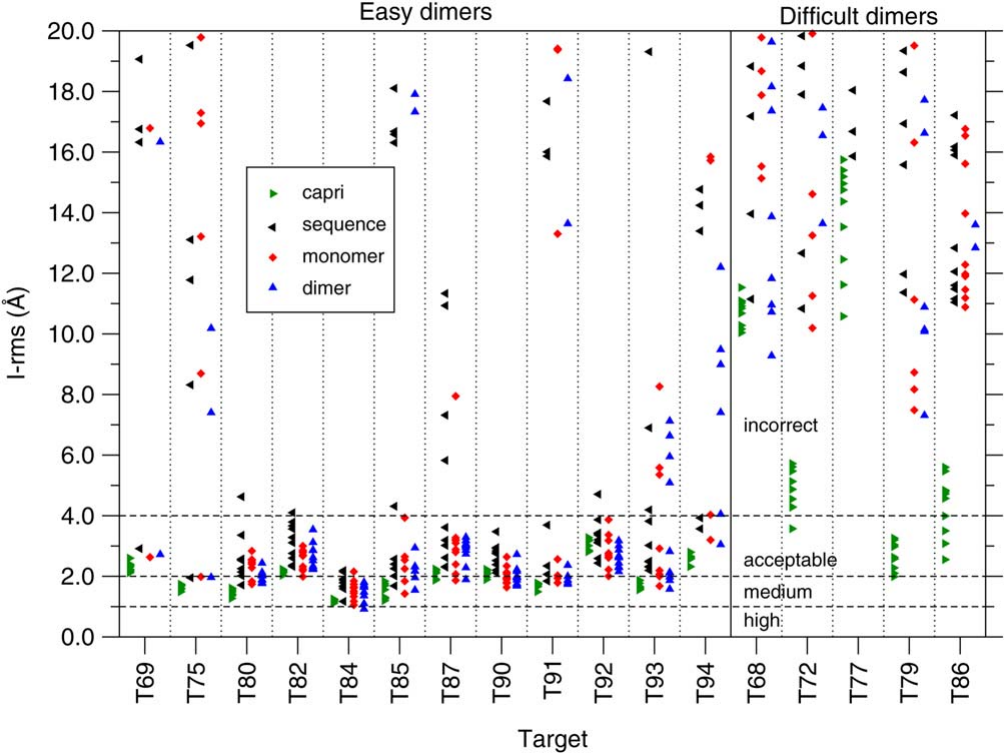
Accuracy of Round 30 homodimer models predicted by protein docking methods and template‐based modeling versus models derived by standard homology modeling. The *I‐rms* values, representing the accuracy level of the predicted interface, are plotted (vertical axis) for different models for each target (listed on the horizontal axis using the CAPRI target identification). Each point represents one model. The best models submitted by individual CAPRI predictor groups are represented by green triangles. The remaining models are those built in this study by standard homology modeling techniques^42^ on the basis of homodimer templates from the PDB. Up to 10 best models are shown per target and template category (see text). Models based on templates identified using sequence information (black triangles), models based structural alignments of individual monomers (red lozenges), and those based on structural alignments of the entire dimers (blue triangles). The targets (only dimers) are subdivided into easy and difficult targets (see text). Dashed horizontal lines represent *I‐rms* values delimiting models of high, medium, acceptable and lower (incorrect) quality by CAPRI criteria.

The accuracy levels of the models built using the three categories of best templates for each target and the best models from each of the participating CAPRI predictor groups submitted for the same target are plotted in Figure 8. The model accuracy is measured by the *I‐rms* value, representing the accuracy level of the predicted interface in the complex. Each entry in the Figure represents one model, and for each template category (based on sequence alignments, on structural alignment of the monomers and dimers, respectively), up to 10 best models are shown per target and colored according to the template category.

Inspection of Figure 8 indicates that models submitted by CAPRI predictor groups, a vast majority of which employed docking methods as part of their protocol, tend to be of higher accuracy. For most of the easy targets, the 10 models submitted by CAPRI groups more consistently display lower *I‐rms* values then the models built from the best templates. This is the case not only for models derived from the sequence‐based templates but also for the most structurally similar templates of the monomer or dimer categories. Considering only the best models for each targets the performance results are mode balanced. For seven out of the 12 easy targets the best models overall were submitted by CAPRI participants, whereas for the remaining five targets the most accurate models were those derived from the structurally most similar template. Overall however, acceptable or medium quality models were obtained with all the approaches and for nearly all the easy targets.

On the other hand it is remarkable that for three of the difficult targets (T72, T79, and T86), the docking procedures were able to produce acceptable models, with one medium quality model for T79, whereas all the template‐based models were incorrect.

Overall these results do confirm that protein docking procedures represent an added value over straightforward template‐based modeling. One must recall however, that docking was often combined with template‐based restraints and hence, can in general not be qualified as *ab‐initio* docking in the context of this experiment. It is also important to note that for two targets, T82 and T85, the highest accuracy models were predicted by the group of Seok, who employed specialized template‐based modeling techniques augmented by loop modeling and refinement. But the accuracy of these models was not vastly superior to that of the best docking models.

Lastly, not too surprisingly, oligomer models build using the sequence‐based best templates were generally of inferior accuracy than models built from templates of the two other categories. Interestingly, models derived from the most structurally similar dimer templates were not generally more accurate than those derived from the structurally most similar monomers. This may stem from differences in the structural alignments that were used to detect the templates, which in turn could have affected the performance of the homology modeling procedure (MODELLER).

## CONCLUDING REMARKS

CAPRI Round 30, for which results were assessed here, was the first CASP‐CAPRI experiment, which brought together the community of groups developing methods for protein structure prediction and model refinement, with groups developing methods for predicting the 3D structure of protein assemblies. The 25 targets of this round represented a subset of the targets submitted for the CASP11 prediction season of the summer of 2014. In line with the main focus of CASP, the majority of these targets were single protein chains, forming mostly homodimers, and a few homotetramers. Only two of the targets were heterodimers, similar to the staple targets in previous CAPRI rounds. Unlike in most previous CAPRI rounds both subunit structures and their association modes had to be modeled for all the targets. Since the docking or assembly modeling performance may crucially depend on the accuracy of the models of individual subunits, the targets chosen for this experiment were proteins deemed to be readily modeled using templates from the PDB. Interestingly, templates were used mainly to model the structures of individual subunits, to limit the sampling space of docking solution or to filter such these solutions. Only a few groups carried out template‐based docking for the majority of the targets, and two of those ranked amongst the top performers, indicating that this relatively recent modeling strategy has potential.

As part of our assessment we established that the accuracy of the models of the individual subunits was an important factor contributing to high accuracy predictions of the corresponding complexes. At the same time we observed that highly accurate models of the protein components are not necessarily required for identifying their association modes with acceptable accuracy.

Furthermore, we provide evidence that protein docking procedures and in some cases also specialized template‐based methods generally outperform off‐the‐shelf template‐based prediction of complexes. These findings apply to templates identified on the basis of sequence information alone, as well as to templates structurally more similar to the target. The added value of docking methods was particularly significant for the more difficult targets, where the structures of the identified best templates differed more significantly from the target structure

Thus, the assessment results presented here confirm that the prediction of homodimer assemblies by homology modeling techniques and docking calculations is feasible, especially for stable dimers that feature interface areas of 1000–1500 Å^2^, whose size is comparable or larger than the one associated with transient heterocomplexes. They also confirm that docking procedures can represent a competitive advantage over standard homology modeling techniques, when those are applied without further improvements to model the complex.

On the other hand, difficulties arise when the subunit interface in the target is similar in size to those associated with crystal contacts.^45^ Such cases were associated with a number of targets where the oligomeric state assignment was ambiguous or inaccurate. Such ambiguous or inaccurate oligomeric state assignments represented a confounding factor for the docking prediction in this round. The problem arose mainly from the fact that the authors' assignments, usually based on independent experiment evidence, were not available to predictors at the time of the prediction experiment. Instead, predictors were provided with tentative assignments, inferred on the basis of computational analysis of the crystal contacts. Quite encouragingly, for most targets with ambiguous assignment, or for which the tentative assignments were later overruled by the authors upon submission to the PDB, the docking predictions were shown to provide useful information, which often confirmed the final assignment or helped resolve ambiguous ones. This occurred for both homodimer and homotetramer targets.

Lastly, we find that the docking prediction performance for the genuine homodimer targets was superior to that obtained for heterocomplexes in previous CAPRI rounds, in line with the expectation that, owing to their higher binding affinity (and larger and more hydrophobic interfaces), homodimers are easier to predict than heterodimers. Much poorer prediction performance was however achieved for genuine tetrameric targets, where the inaccuracy of the homology‐built subunit models and the smaller pair‐wise interfaces limited the prediction performance. Accurately modeling of higher order assemblies from sequence information is thus an area where progress is needed.

## Supporting information

Supporting Information
