# Supplementary material for: Prediction of homoprotein and heteroprotein complexes by protein docking and template‐based modeling: A CASP‐CAPRI experiment
Source: Proteins. 2016 Jun 1;84(Suppl Suppl 1):323–48. doi: 10.1002/prot.25007 (PMC5030136; doi:10.1002/prot.25007)
Supplement: Supplementary file 1 — Supporting Information [file PROT-84-323-s001.pdf]

Prediction of homo- and hetero-protein complexes by protein docking and template-based modeling: a CASP-CAPRI experiment

Marc F. Lensink, Sameer Velankar, Andriy Kryshtafovych,
all CASP-CAPRI participants and Shoshana J. Wodak

Supplementary Material

– Table S1 – List of assessed interfaces **2**

– Table S2 – Prediction results per target **3**

The tables list for every target the performance of CAPRI Predictor and Scorer groups, and of CASP Predictor groups. The results of automatic servers (listed in all capital letters) are included in their respective predictor group. Results are listed as the number of submitted models of acceptable quality or better, with the number of higher than acceptable quality models listed after a slash, *e.g.* '9/8**' indicates that 9 models of acceptable quality or better were submitted, of which 8 are of medium quality. CAPRI groups were allowed to submit 10 models, CASP groups 5. Incorrect models are not listed.

– Table S3 – Target submission results **28**

– Table S4 – Best quality models **30**

– Table S5 – Target template data **31**

– Table S6 – Supplementary methods **37**

– Figure S1 – Evaluation protocol for higher-order oligomers **66**

Interface	Symmetry operation	Area (Å^2)
T68.1	X,Y,Z	1150
T68.2	X,Y,Z	860
T69.1	Y,X,-Z+1	2410
T69.2	X-1/2,-Y+1/2,-Z+3/4	620
T70.1	-X+1/2,Y-1/2,-Z-1	555
T70.2	X,Y,Z	460
T71.1	X,Y,Z	720
T71.2	X-1/2,-Y+3/2,-Z+1	540
T71.3	X,Y,Z	470
T72.1	X,Y,Z	1120
T72.2	Y,X-1,-Z	650
T72.3	-Y,X-Y-1,Z+1/3	610
T73.1	X,Y,Z	2430
T73.2	X,Y,Z	520
T74.1	-X+1,-Y+1,Z	520
T74.2	X-1/2,-Y+1/2,-Z+1/2	490
T75.1	X,Y,Z	1040
T77.1	-X+1,-Y,Z	1600
T77.2	-Y+1,-X+1,-Z+1/3	640
T77.3	X,X-Y-1,-Z+2/3	510
T77.4	X-Y,X,Z-1/3	470
T78.1	X-Y,-Y,-Z	1460
T78.2	-X+Y+1,Y,-Z	620
T79.1	X,Y,Z	680
T79.2	-Y,X-Y-1,Z-1/3	440
T79.3	X-Y,X,Z-1/6	400
T80.1	X,Y,Z	1960
T81.1	X,Y,Z	1070
T82.1	X,X-Y,-Z+1/6	3250
T84.1	X,Y,Z	1740
T85.1	X,Y,Z	4620
T86.1	X,Y-1,Z	470
T86.2	-X+1,Y-1/2,-Z+2	420
T87.1	-X,Y,-Z+2	3430
T88.1	X,Y,Z	1350
T89.1	X,Y,Z	870
T90.1	X,Y,Z	2360
T91.1	X,Y,Z	1320
T92.1	X,Y,Z	1900
T93.1	X,Y,Z	2680
T94.1	X,Y,Z	1190

Table S1 – List of assessed interfaces. The interface is listed by the CAPRI target number with the digit after the dot representing the rank of the interface, determined on the interface area. The symmetry operations and interface areas are provided by the PISA tool. Interface areas are rounded to the nearest 10 Å^2.

Target T68

Target 68 Interface 1
no acceptable models

Target 68 Interface 2
no acceptable models

Table S2.68 – Prediction results for Target 68.

Target T69

Target 69 Interface 1

**CAPRI Predictors**	**87/57****
Seok	10/10**
Lee	10/10**
Guerois	10/10**
Zou	10/9**
Shen	9/8**
Eisenstein	10/5**
Huang	4/2**
Vajda/Kozakov	2/1**
CLUSPRO	2/1**
Weng	1/1**
HADDOCK	10
Sali	6
Negi	1
Fernandez-Recio	1
DOCK_PIERR	1
**CAPRI Scorers**	**69/45****
Kihara	10/10**
Zou	9/8**
Grudinin	7/5**
Huang	5/3**
Gray	5/3**
Bonvin	4/3**
Seok	3/3**
Oliva	10/2**
Bates	9/2**
Fernandez-Recio	3/2**
Weng	2/2**
Lee	2/2**
**CASP Predictors**	**2/1****
Baker	1/1**
Umeyama	1

Target 69 Interface 2

no acceptable models

Table S2.69 – Prediction results for Target 69.

Target T70

Target 70 Interface 1	
no acceptable models	

Target 70 Interface 2	
**CAPRI Predictors**	**37/27****
Vajda/Kozakov	10/10**
CLUSPRO	10/10**
HADDOCK	8/7**
Lee	6
Vakser	1
Negi	1
Huang	1
**CAPRI Scorers**	**27/21****
Bates	10/10**
Bonvin	8/6**
Lee	9/5**
**CASP Predictors**	**0**

Target 70 Interface 3	
no acceptable models	

Table S2.70 – Prediction results for Target 70.

Target T71

Target 71 Interface 1

**CAPRI Predictors**	**1**
GRAMM-X	1
**CAPRI Scorers**	**0**
**CASP Predictors**	**0**

Target 71 Interface 2

no acceptable models

Target 71 Interface 3

**CAPRI Predictors**	**11**
CLUSPRO	5
HADDOCK	4
Sali	1
Huang	1
**CAPRI Scorers**	**6**
Bonvin	2
Bates	2
Zou	1
Lee	1
**CASP Predictors**	**0**

Table S2.71 – Prediction results for Target 71.

Target T72

Target 72 Interface 1

**CAPRI Predictors**	**3**
SWARMDOCK	1
HADDOCK	1
Guerois	1
**CAPRI Scorers**	**7**
Bonvin	2
Weng	1
Huang	1
Grudinin	1
Gray	1
Fernandez-Recio	1
**CASP Predictors**	**0**

Target 72 Interface 2

no acceptable models

Target 72 Interface 3

no acceptable models

Table S2.72 – Prediction results for Target 72.

Target T73

Target 73 Interface 1

**CAPRI Predictors**	**9**
LZERD	5
Zou	2
Kihara	2
**CAPRI Scorers**	**0**
**CASP Predictors**	**0**

Target 73 Interface 2

**CAPRI Predictors**	**2**
Lee	2
**CAPRI Scorers**	**0**
**CASP Predictors**	**0**

Table S2.73 – Prediction results for Target 73.

Target T74

Target 74 Interface 1
no acceptable models

Target 74 Interface 2
no acceptable models

Table S2.74 – Prediction results for Target 74.

Target T75

Target 75 Interface 1	
**CAPRI Predictors**	**65/47****
Shen	10/10**
Sali	10/10**
Lee	9/9**
Zou	10/4**
Guerois	6/4**
Eisenstein	6/3**
Huang	3/1**
Weng	2/1**
Seok	2/1**
Vajda/Kozakov	1/1**
Tovchigrechko	1/1**
GRAMM-X	1/1**
CLUSPRO	1/1**
HADDOCK	2
Kihara	1
**CAPRI Scorers**	**35/26****
Oliva	10/10**
Kihara	7/5**
LZERD	4/4**
Fernandez-Recio	3/3**
Bonvin	4/2**
Huang	3/1**
Weng	2/1**
Seok	1
Bates	1
**CASP Predictors**	**5/5****
Baker	3/3**
Umeyama	1/1**
Dunbrack	1/1**

Table S2.75 – Prediction results for Target 75.

Target T77

Target 77 Interface 1
no acceptable models

Target 77 Interface 2
no acceptable models

Target 77 Interface 3
no acceptable models

Target 77 Interface 4
no acceptable models

Table S2.77 – Prediction results for Target 77.

Target T78

Target 78 Interface 1
no acceptable models

Target 78 Interface 2
no acceptable models

Table S2.78 – Prediction results for Target 78.

Target T79

Target 79 Interface 1

**CAPRI Predictors**	**16/5****
Seok	6/4**
Sali	2/1**
Guerois	2
Bates	2
Zhou	1
Tovchigrechko	1
Negi	1
GRAMM-X	1
**CAPRI Scorers**	**10/4****
Kihara	5/1**
Bonvin	2/1**
Seok	1/1**
Gray	1/1**
Zou	1
**CASP Predictors**	**1**
Dunbrack	1

Target 79 Interface 2

**CAPRI Predictors**	**12/1****
Bates	4/1**
Kihara	3
Xiao	1
LZERD	1
Guerois	1
Grudinin	1
Fernandez-Recio	1
**CAPRI Scorers**	**13/3****
Zou	2/1**
Fernandez-Recio	2/1**
Bonvin	2/1**
Bates	3
Grudinin	2
Seok	1
Huang	1
**CASP Predictors**	**0**

Target 79 Interface 3

**CAPRI Predictors**	**0**
**CAPRI Scorers**	**2**
Weng	1
Huang	1
**CASP Predictors**	**0**

Table S2.79 – Prediction results for Target 79.

Target T80

Target 80 Interface 1	
**CAPRI Predictors**	**105/71****
Sali	10/10**
Guerois	10/10**
Eisenstein	10/10**
Seok	10/9**
Zou	10/6**
Shen	7/6**
Lee	10/5**
Vakser	5/3**
Weng	3/3**
Huang	4/2**
Tomii	3/2**
Grudinin	3/2**
Ritchie	3/1**
Negi	1/1**
Fernandez-Recio	1/1**
HADDOCK	10
Vajda/Kozakov	1
Tovchigrechko	1
GRAMM-X	1
CLUSPRO	1
Bates	1
**CAPRI Scorers**	**65/51****
Zou	10/10**
Huang	10/10**
Gray	8/8**
Kihara	6/5**
Weng	5/5**
Fernandez-Recio	6/4**
Bates	5/4**
Grudinin	2/2**
Bonvin	2/2**
Seok	1/1**
Oliva	10
**CASP Predictors**	**13/11****
ROSETTASERVER	5/5**
Umeyama	4/3**
Luethy	2/2**
SEOK_SERVER	1/1**
Nakamura	1

Table S2.80 – Prediction results for Target 80.

Target T81

Target 81 Interface 1	
**CAPRI Predictors**	**12/5****
Guerois	3/3**
Seok	4/1**
Huang	1/1**
Vajda/Kozakov	1
SWARMDOCK	1
CLUSPRO	1
Bates	1
**CAPRI Scorers**	**54/16****
Bates	10/7**
LZERD	8/3**
Oliva	5/3**
Huang	6/1**
Fernandez-Recio	6/1**
Seok	1/1**
Kihara	9
Grudinin	4
Zou	2
Bonvin	2
Weng	1
**CASP Predictors**	**0**

Table S2.81 – Prediction results for Target 81.

Target T82

Target 82 Interface 1	
**CAPRI Predictors**	**71/54****
HADDOCK	10/10**
Guerois	10/10**
Seok	10/8**
Shen	7/7**
Fernandez-Recio	8/5**
Huang	5/3**
Weng	4/3**
Grudinin	4/3**
Zou	7/1**
Vajda/Kozakov	2/1**
CLUSPRO	2/1**
Negi	1/1**
Lee	1/1**
**CAPRI Scorers**	**99/77****
Bates	10/10**
LZERD	10/9**
Huang	10/9**
Gray	10/9**
Fernandez-Recio	10/9**
Kihara	10/8**
Zou	10/7**
Bonvin	7/5**
Oliva	10/4**
Seok	4/3**
Grudinin	5/2**
Weng	3/2**
**CASP Predictors**	**11/9****
ROSETTASERVER	5/5**
Umeyama	5/3**
Dunbrack	1/1**

Table S2.82 – Prediction results for Target 82.

Target T84

Target 84 Interface 1	
**CAPRI Predictors**	**84/61****
Zou	10/10**
HADDOCK	10/10**
Seok	10/9**
Shen	6/5**
Vakser	5/5**
Weng	4/4**
Grudinin	4/4**
Huang	8/3**
Guerois	9/2**
Fernandez-Recio	3/2**
Lee	5/1**
Kihara	4/1**
Vajda/Kozakov	1/1**
Tomii	1/1**
SWARMDOCK	1/1**
Negi	1/1**
CLUSPRO	1/1**
Zhou	1
**CAPRI Scorers**	**81/72****
Zou	10/10**
Weng	10/10**
Oliva	10/10**
Bates	10/10**
Huang	9/8**
Gray	10/7**
Kihara	7/7**
Bonvin	7/4**
Grudinin	3/3**
Fernandez-Recio	4/2**
Seok	1/1**
**CASP Predictors**	**18/15****
Umeyama	5/5**
ROSETTASERVER	5/5**
Baker	4/2**
SEOK_SERVER	1/1**
Luethy	1/1**
Dunbrack	1/1**
Nakamura	1

Table S2.84 – Prediction results for Target 84.

Target T85

Target 85 Interface 1	
**CAPRI Predictors**	**83/55****
HADDOCK	10/10**
Shen	8/7**
Seok	10/6**
Guerois	10/5**
Zou	10/4**
Fernandez-Recio	10/4**
Weng	7/4**
Vakser	5/4**
Huang	5/4**
Grudinin	4/3**
SWARMDOCK	2/2**
Vajda/Kozakov	1/1**
CLUSPRO	1/1**
**CAPRI Scorers**	**103/75****
LZERD	10/10**
Oliva	10/9**
Kihara	10/8**
Gray	10/8**
Zou	10/7**
Huang	10/7**
Grudinin	10/7**
Bonvin	10/6**
Bates	10/6**
Weng	10/4**
Seok	3/3**
**CASP Predictors**	**11/8****
ROSETTASERVER	5/3**
Umeyama	4/3**
Nakamura	1/1**
Dunbrack	1/1**

Table S2.85 – Prediction results for Target 85.

Target T86

Target 86 Interface 1

**CAPRI Predictors**	**3**
Ritchie	2
Negi	1
**CAPRI Scorers**	**5**
Fernandez-Recio	4
Gray	1
**CASP Predictors**	**0**

Target 86 Interface 2

**CAPRI Predictors**	**0**
**CAPRI Scorers**	**5/1****
Seok	1/1**
Kihara	4
**CASP Predictors**	**0**

Table S2.86 – Prediction results for Target 86.

Target T87

Target 87 Interface 1	
**CAPRI Predictors**	**83/51****
Zou	10/10**
HADDOCK	10/10**
Guerois	10/9**
Huang	10/5**
Seok	10/4**
Vakser	10/3**
Shen	6/3**
Weng	2/2**
Zhou	3/1**
Fernandez-Recio	3/1**
Vajda/Kozakov	1/1**
Grudinin	1/1**
CLUSPRO	1/1**
SWARMDOCK	5
Tomii	1
**CAPRI Scorers**	**85/66****
Oliva	10/10**
Kihara	10/10**
Zou	10/9**
Fernandez-Recio	10/6**
Bates	10/6**
Weng	9/6**
Bonvin	7/5**
Gray	5/5**
Huang	7/4**
Grudinin	6/4**
Seok	1/1**
**CASP Predictors**	**12/10****
ROSETTASERVER	5/5**
Umeyama	4/2**
SEOK_SERVER	1/1**
Luethy	1/1**
Dunbrack	1/1**

Table S2.87 – Prediction results for Target 87.

Target T88

Target 88 Interface 1
no acceptable models

Table S2.88 – Prediction results for Target 88.

Target T89

Target 89 Interface 1	
**CAPRI Predictors**	**87/26****
HADDOCK	10/8**
Vakser	10/4**
Seok	9/4**
Guerois	4/4**
Grudinin	10/3**
Lee	10/1**
Huang	5/1**
Tomii	1/1**
Zou	10
Weng	8
Shen	5
Bates	2
Vajda/Kozakov	1
SWARMDOCK	1
CLUSPRO	1
**CAPRI Scorers**	**46/14****
Oliva	10/4**
Kihara	9/4**
Zou	9/3**
Bates	8/1**
Huang	4/1**
Bonvin	4/1**
Weng	1
Seok	1
**CASP Predictors**	**23/6****
SEOK_SERVER	5/3**
Wallner_refine	3/2**
Wallner	2/1**
Umeyama	5
NNS_Lee	5
Skwark	1
RAPTORX_Wang	1
Dunbrack	1

Table S2.89 – Prediction results for Target 89.

Target T90

Target 90 Interface 1	
**CAPRI Predictors**	**104/47****
HADDOCK	10/10**
Seok	10/8**
Huang	10/6**
Vakser	8/6**
Guerois	10/4**
Zou	10/2**
Shen	10/2**
Lee	10/2**
Zhou	3/1**
Grudinin	3/1**
Vajda/Kozakov	2/1**
Fernandez-Recio	2/1**
CLUSPRO	2/1**
Tomii	1/1**
SWARMDOCK	1/1**
Bates	7
Weng	2
GRAMM-X	2
Kihara	1
**CAPRI Scorers**	**57/38****
Oliva	10/9**
Zou	10/8**
Weng	5/5**
Bates	9/4**
Huang	6/4**
Bonvin	5/4**
Fernandez-Recio	5/2**
Grudinin	3/2**
Kihara	3
Seok	1
**CASP Predictors**	**13/7****
Umeyama	5/5**
Nakamura	1/1**
Dunbrack	1/1**
ROSETTASERVER	5
SEOK_SERVER	1

Table S2.90 – Prediction results for Target 90.

Target T91

Target 91 Interface 1	
**CAPRI Predictors**	**109/40****
Seok	10/10**
HADDOCK	10/10**
Zou	8/4**
Vakser	7/4**
Grudinin	7/3**
Huang	3/3**
Shen	10/2**
SWARMDOCK	10/1**
Guerois	10/1**
Sali	4/1**
Weng	2/1**
Kihara	8
Fernandez-Recio	6
Vajda/Kozakov	5
CLUSPRO	5
LZERD	2
Bates	2
**CAPRI Scorers**	**91/64****
Oliva	10/9**
Grudinin	10/8**
Kihara	10/7**
Zou	10/6**
Bates	10/6**
Fernandez-Recio	9/6**
Bonvin	8/6**
Weng	7/6**
Huang	9/4**
LZERD	5/4**
Seok	3/2**
**CASP Predictors**	**10/6****
ROSETTASERVER	5/5**
SEOK_SERVER	1/1**
Nakamura	2
Umeyama	1
Dunbrack	1

Table S2.91 – Prediction results for Target 91.

Target T92

Target 92 Interface 1	
**CAPRI Predictors**	**98/12****
Huang	7/3**
Shen	10/2**
Seok	10/2**
Fernandez-Recio	2/2**
Vakser	9/1**
Tomii	5/1**
Grudinin	5/1**
SWARMDOCK	10
Guerois	10
Zou	9
Lee	6
HADDOCK	6
Weng	5
Vajda/Kozakov	1
GRAMM-X	1
Fernandez-Fuentes	1
CLUSPRO	1
**CAPRI Scorers**	**68/32****
Bates	10/9**
Zou	10/6**
Bonvin	6/5**
Huang	8/2**
Fernandez-Recio	8/2**
Grudinin	7/2**
Weng	6/2**
Kihara	5/2**
Seok	2/2**
Oliva	6
**CASP Predictors**	**17/3****
ROSETTASERVER	5/3**
Umeyama	5
Nakamura	3
Luethy	2
SEOK_SERVER	1
Dunbrack	1

Table S2.92 – Prediction results for Target 92.

Target T93

Target 93 Interface 1	
**CAPRI Predictors**	**102/70****
Shen	10/10**
HADDOCK	10/10**
Guerois	10/7**
Fernandez-Recio	10/7**
Zou	10/6**
Vakser	6/6**
Bates	6/6**
Seok	7/5**
Grudinin	5/4**
Huang	4/3**
Kihara	9/2**
Weng	2/2**
Tomii	2/2**
SWARMDOCK	9
Vajda/Kozakov	1
CLUSPRO	1
**CAPRI Scorers**	**77/62****
Fernandez-Recio	10/9**
Oliva	10/8**
Bates	9/8**
Zou	9/7**
Kihara	10/6**
Weng	8/6**
Grudinin	7/6**
Huang	6/5**
LZERD	4/4**
Bonvin	3/2**
Seok	1/1**
**CASP Predictors**	**13/12****
ROSETTASERVER	5/5**
Umeyama	4/4**
Nakamura	3/2**
Luethy	1/1**

Table S2.93 – Prediction results for Target 93.

Target T94

Target 94 Interface 1	
**CAPRI Predictors**	**58/1****
Zou	6/1**
HADDOCK	10
SWARMDOCK	8
Guerois	6
Tomii	5
Shen	5
Seok	5
Weng	3
Vakser	3
Huang	3
Vajda/Kozakov	2
CLUSPRO	2
**CAPRI Scorers**	**37/1****
Seok	1/1**
Oliva	10
Bates	10
Zou	7
Huang	4
Bonvin	3
Weng	1
Gray	1
**CASP Predictors**	**2**
Umeyama	1
Dunbrack	1

Table S2.94 – Prediction results for Target 94.

	Target																								
**CAPRI Predictors**	68	69	70	71	72	73	74	75	77	78	79	80	81	82	84	85	86	87	88	89	90	91	92	93	94	Performance
Seok	0	**	0	0	0	0	0	**	0	0	**	**	**	**	**	**	0	**	0	**	**	**	**	**	*	15/14**
Huang	0	**	*	*	0	0	0	**	0	0	0	**	**	**	**	**	0	**	0	**	**	**	**	**	*	16/13**
Guerois	0	**	0	0	*	0	0	**	0	0	*	**	**	**	**	**	0	**	0	**	**	**	*	**	*	16/12**
Zou	0	**	0	0	0	*	0	**	0	0	0	**	0	**	**	**	0	**	0	*	**	**	*	**	**	14/11**
Shen	0	**	0	0	0	0	0	**	0	0	0	**	0	**	**	**	0	**	0	*	**	**	**	**	*	13/11**
Grudinin	-	0	0	0	0	0	0	0	0	0	*	**	0	**	**	**	0	**	0	**	**	**	**	**	0	11/10**
Weng	0	**	0	0	0	0	0	**	0	0	0	**	0	**	**	**	0	**	0	*	*	**	*	**	*	13/9**
Vakser	0	0	*	0	0	0	0	0	0	0	0	**	0	0	**	**	0	**	0	**	**	**	**	**	*	11/9**
Vajda/Kozakov	0	**	**	-	0	0	0	**	0	0	0	*	*	**	**	**	0	**	0	*	**	*	*	*	*	15/8**
Fernandez-Recio	0	*	0	0	0	0	0	0	0	0	*	**	0	**	**	**	0	**	0	0	**	*	**	**	0	11/8**
Lee	-	**	*	0	0	*	0	**	-	0	0	**	0	**	**	-	0	-	0	**	**	-	*	0	0	10/7**
Tomii	-	-	0	0	0	0	0	0	0	0	0	**	0	-	**	-	0	*	0	**	**	-	**	**	*	8/6**
Sali	-	*	0	*	-	0	-	**	0	0	**	**	-	-	-	-	0	-	0	-	-	**	-	-	-	6/4**
Negi	0	*	*	0	0	0	0	0	0	0	*	**	0	**	**	0	*	0	0	0	0	0	0	0	0	7/3**
Eisenstein	-	**	-	-	0	-	-	**	-	-	0	**	-	-	-	-	-	-	0	-	-	-	-	-	-	3/3**
Kihara	0	0	0	0	0	*	-	*	0	0	*	0	-	0	**	0	0	0	0	0	*	*	0	**	0	7/2**
Bates	0	0	0	0	0	0	0	0	0	0	**	*	*	0	0	0	0	0	0	*	*	*	0	**	0	7/2**
Zhou	0	0	0	0	0	0	0	0	0	0	*	0	0	0	*	0	0	**	0	0	**	0	0	0	0	4/2**
Tovchigrechko	-	-	-	-	-	-	0	**	0	0	*	*	0	-	0	0	0	-	0	-	-	0	-	-	-	3/1**
Ritchie	-	-	-	-	-	-	-	-	-	-	-	**	0	0	0	0	*	0	0	-	-	-	-	-	-	2/1**
Xiao	0	0	0	0	0	0	0	0	0	0	*	-	-	-	-	-	-	-	-	-	-	-	-	-	-	1
Fernandez-Fuentes	0	-	-	-	0	-	-	0	-	-	-	0	-	0	0	-	0	0	-	0	0	0	*	0	0	1
Wade	0	0	-	-	-	-	-	-	-	-	-	-	-	-	-	-	-	-	-	-	-	-	-	-	-	0
Haliloglu	-	-	-	-	-	-	-	-	-	-	-	-	-	-	-	-	-	-	0	-	-	-	-	-	-	0
Gong	0	-	-	-	-	-	-	-	-	-	-	0	0	0	-	0	0	0	0	-	-	-	-	-	-	0
Del Carpio	-	-	-	-	-	-	-	-	-	-	-	-	-	-	-	-	-	-	-	-	0	0	0	-	-	0

Table S3 – Target submission results. For every target and participant group, the quality of the best submitted model is listed, with '***', '**', '*' and '0' representing 'high', 'medium', 'acceptable' and 'incorrect' models, resp. Results are separated by group, with CASP Predictor and Server groups taken together. Servers are listed in all capital letters. A dash ('-') indicates that no submission was done for that target. The final column indicates the overall performance in CAPRI Round 30. The table continues on the following page.

	Target																									
**CAPRI Servers**	68	69	70	71	72	73	74	75	77	78	79	80	81	82	84	85	86	87	88	89	90	91	92	93	94	Performance
HADDOCK	0	*	**	*	*	0	0	*	0	0	0	*	0	**	**	**	0	**	0	**	**	**	*	**	*	16/9**
CLUSPRO	0	**	**	*	0	0	0	**	0	0	0	*	*	**	**	**	0	**	0	*	**	*	*	*	*	16/8**
SWARMDOCK	0	0	0	0	*	0	0	0	0	0	0	0	*	0	**	**	0	*	0	*	**	**	*	*	*	11/4**
GRAMM-X	-	0	0	*	0	0	0	**	0	0	*	*	0	-	0	0	0	0	-	0	*	0	*	0	0	6/1**
LZERD	0	0	0	0	0	*	0	0	0	0	*	0	0	0	0	0	0	0	0	0	0	*	0	0	0	3
DOCK_PIERR	0	*	-	-	-	-	-	-	-	-	-	-	-	-	-	-	-	-	-	-	-	-	-	-	-	1
**CAPRI Scorers**	68	69	70	71	72	73	74	75	77	78	79	80	81	82	84	85	86	87	88	89	90	91	92	93	94	Performance
Bonvin	0	**	**	*	*	0	0	**	0	0	**	**	*	**	**	**	0	**	0	**	**	**	**	**	*	18/14**
Bates	0	**	**	*	0	-	0	*	0	0	*	**	**	**	**	**	0	**	0	**	**	**	**	**	*	17/13**
Seok	0	**	0	0	0	0	0	*	0	0	**	**	**	**	**	**	**	**	0	*	*	**	**	**	**	16/13**
Huang	0	**	0	0	*	0	0	**	0	0	*	**	**	**	**	**	0	**	0	**	**	**	**	**	*	16/13**
Zou	0	**	0	*	0	0	0	0	0	0	**	**	*	**	**	**	0	**	0	**	**	**	**	**	*	15/12**
Kihara	0	**	0	0	0	0	0	**	0	0	**	**	*	**	**	**	*	**	0	**	*	**	**	**	0	15/12**
Fernandez-Recio	0	**	0	0	*	0	0	**	0	0	**	**	**	**	**	0	*	**	0	0	**	**	**	**	0	14/12**
Weng	0	**	0	0	*	0	0	**	0	0	*	**	*	**	**	**	0	**	0	*	**	**	**	**	*	16/11**
Oliva	0	**	0	0	0	-	-	**	0	-	0	*	**	**	**	**	0	**	0	**	**	**	*	**	*	14/11**
Grudinin	0	**	0	0	*	0	0	0	0	0	*	**	*	**	**	**	0	**	0	0	**	**	**	**	0	13/10**
Gray	0	**	0	0	*	-	-	0	0	0	**	**	-	**	**	**	*	**	0	-	-	-	-	-	*	10/7**
LZERD	0	0	0	0	0	0	0	**	0	0	0	0	**	**	0	**	0	0	0	0	0	**	0	**	0	6/6**
Lee	-	**	**	*	0	0	-	-	-	-	-	-	-	-	-	-	-	-	-	-	-	-	-	-	-	3/2**
Sali	-	-	-	-	-	-	-	-	-	-	-	-	-	-	-	-	-	-	0	-	-	-	-	-	-	0
**CASP Predictors**	68	69	70	71	72	73	74	75	77	78	79	80	81	82	84	85	86	87	88	89	90	91	92	93	94	Performance
Umeyama	-	*	-	-	0	0	-	**	-	0	0	**	-	**	**	**	0	**	0	*	**	*	*	**	*	13/8**
ROSETTASERVER	-	-	-	-	-	0	-	0	-	-	-	**	-	**	**	**	0	**	-	0	*	**	**	**	-	9/8**
Dunbrack	-	-	-	-	-	-	-	**	-	-	*	-	-	**	**	**	0	**	-	*	**	*	*	-	*	11/6**
SEOK_SERVER	0	0	0	0	0	0	0	0	0	-	0	**	0	-	**	-	0	**	0	**	*	**	*	0	0	7/5**
Luethy	-	-	-	-	-	-	-	-	-	-	0	**	-	-	**	-	0	**	0	-	-	-	*	**	-	5/4**
Nakamura	-	-	0	-	-	-	0	-	-	-	-	*	0	0	*	**	0	-	-	-	**	*	*	**	-	7/3**
Baker	-	**	0	-	-	-	0	**	0	0	-	-	0	-	**	-	-	-	-	-	-	-	-	-	-	3/3**
Wallner	-	-	-	-	-	-	-	-	-	-	-	-	-	-	-	-	-	-	-	**	-	-	-	-	-	1/1**
Skwark	-	-	-	-	-	-	-	-	-	-	-	-	-	-	-	-	-	-	-	*	-	-	-	-	-	1
RAPTORX_Wang	-	-	-	-	-	-	-	-	-	-	0	-	0	-	-	-	0	-	-	*	-	-	-	-	-	1
NNS_Lee	-	-	-	-	-	-	-	-	-	-	-	-	0	-	-	-	-	-	0	*	-	-	-	-	-	1
39 participants not listed																										

Target	Participant	Model	f(nat)	d_L	n_{clash}	L-rms	I-rms	S-rms	θ_L	classification
68.1	**P.**HADDOCK	5	0.000	21.6	7	27.656	10.038	12.786	175.08	incorrect
68.2	**P.**Gong	7	0.000	20.0	93	26.333	11.567	12.022	116.02	incorrect
69.1	**P.**Guerois	6	0.493	1.5	36	2.877	2.117	3.899	5.96	medium
69.2	**P.**DOCK_PIERR	6	0.000	60.4	8	64.997	12.707	13.556	113.70	incorrect
70.1	**P.**Sali	3	0.000	11.4	46	12.682	4.619	5.601	43.94	incorrect
70.2	**S.**Lee	6	0.611	4.0	3	5.582	1.195	2.697	22.88	medium
71.1	**P.**CLUSPRO	10	0.071	25.4	48	28.894	3.092	3.655	78.83	incorrect
71.2	**S.**Bates	4	0.000	11.8	11	20.509	8.650	9.541	130.40	incorrect
71.3	**P.**Sali	7	0.375	10.9	40	16.468	2.165	3.373	44.83	acceptable
72.1	**P.**Guerois	10	0.292	7.3	22	8.851	3.574	5.601	17.65	acceptable
72.2	**P.**Zou	1	0.000	36.6	22	40.074	10.606	11.116	65.39	incorrect
72.3	**S.**Lee	9	0.025	43.9	6	52.966	11.748	12.421	165.94	incorrect
73.1	**P.**Vakser	6	0.073	5.6	3	6.666	2.493	3.922	11.46	incorrect
73.2	**P.**Lee	1	0.167	2.5	11	4.380	3.750	6.479	13.68	acceptable
74.1	**P.**Seok	5	0.000	41.9	4	46.697	5.625	6.446	48.34	incorrect
74.2	**P.**SWARMDOCK	8	0.000	49.2	8	57.929	6.938	8.447	116.16	incorrect
75.1	**C.**Baker	2	0.589	2.4	8	3.772	1.377	2.751	8.00	medium
77.1	**C.**Baker	1	0.156	12.8	20	19.634	10.579	11.472	36.55	incorrect
77.2	**S.**Weng	1	0.000	52.5	7	61.808	5.337	7.252	129.59	incorrect
77.3	**P.**Vajda/Kozakov	2	0.000	39.6	10	55.347	10.593	11.508	178.46	incorrect
77.4	**S.**LZERD	7	0.080	46.9	8	58.213	8.162	9.752	163.27	incorrect
78.1	**P.**Grudinin	3	0.027	8.6	70	11.282	5.690	6.696	22.19	incorrect
78.2	**P.**Vakser	1	0.481	13.0	14	16.085	5.427	6.587	41.45	incorrect
79.1	**P.**Seok	7	0.365	2.0	4	3.250	1.989	2.830	10.31	medium
79.2	**S.**Fernandez-Recio	8	0.640	4.1	9	4.768	2.118	3.199	8.64	medium
79.3	**S.**Huang	2	0.259	4.7	20	8.243	4.665	6.549	38.91	acceptable
80.1	**S.**Huang	7	0.812	1.2	13	2.177	1.197	2.296	4.92	medium
81.1	**S.**Bates	2	0.750	2.1	9	2.832	1.602	3.039	12.46	medium
82.1	**P.**Seok	10	0.542	0.4	28	2.546	2.033	3.906	3.16	medium
84.1	**C.**Luethy	1	0.855	1.2	50	2.604	1.106	1.986	5.07	medium
85.1	**P.**Seok	5	0.665	0.3	19	1.508	1.198	2.697	1.44	medium
86.1	**P.**Ritchie	9	0.667	7.4	24	8.972	2.549	4.623	27.84	acceptable
86.2	**S.**Seok	4	1.000	3.7	4	4.706	1.036	2.352	14.59	medium
87.1	**C.**ROSETTASERVER	4	0.519	1.6	25	2.747	1.797	2.568	5.27	medium
88.1	**S.**Bonvin	5	0.188	8.4	12	16.578	5.604	6.555	100.79	incorrect
89.1	**S.**Kihara	10	0.736	5.0	4	5.742	1.282	2.018	11.51	medium
90.1	**P.**Huang	6	0.548	1.8	19	3.197	1.887	3.277	7.52	medium
91.1	**S.**Kihara	6	0.659	3.8	2	5.258	1.455	2.852	16.84	medium
92.1	**S.**Kihara	4	0.512	2.4	1	4.214	2.584	3.913	10.43	medium
93.1	**C.**ROSETTASERVER	2	0.582	0.4	10	1.909	1.473	3.390	2.27	medium
94.1	**P.**SWARMDOCK	9	0.603	2.3	9	5.021	2.318	3.605	12.87	acceptable

Table S4 – Best model according to I-rms, for each assessed interface, with the interface listed as target number dot interface rank. Lines are colored green, blue and gray for medium, acceptable and incorrect models, resp. The participant producing this model and the model rank in the set of submitted models follow in columns 2 and 3. CAPRI Predictors (including servers), CAPRI Scorers, and CASP Predictors preceded by "P.", "S." and "C.", resp. Values for d_L, and L-rms, I-rms and S-rms are given in Å, and θ_L in degrees.

Target	Template	Seok	Kihara	Tomii	Guerois	Zou	Vakser	Weng	Shen	Negi	Sali	Xiao	Eisenstein	Zhou	Ritchie	Elber
T68	1lm5	×	×	×								×				
	1lm7	×	×							×						
	Additional templates used by:															
	Vakser	1cz7 1zva 2fzf 2giu 2p1t 3clc 3e7l 3i1g 3lr4														
	Kihara	1f60														
	Huang	3gsz														
T69	1qlw	×	×	×		×		×	×		×	×				×
	Additional templates used by:															
	Vakser	2dst 2ocg 2qjw 3bjr 3d2a 3dqz 3f67 3fcx 3llc 3pfb														
	Xiao	1xt8 3zm5														
	Tomii	2wkw														
	Huang	3h3l														
	Eisenstein	1wkw														
T70	*Templates used by:*															
	Vakser	1oia 1vc1 1zik 2xmj 2xnq 3bs9 3ewg 3iwl 3p3d 3u2e														
	Xiao	2ca9 2wve 3aqf														
	Tomii	2w7v 3khn														
	Seok	3sy8														
	Kihara	3aqp														
	Huang	3sy6														
T71	4gt6	×	×									×				
	Additional templates used by:															
	Vakser	1ezg 1o6t 1wwl 2o6r 2wfh 3fy3 3g39 3pet 3n90														
	Xiao	4fdw 4fs7														
	Tomii	2o6s 2v70														
	Huang	3rjt														
T72	3mx3		×		×				×			×	×			
	3gzs	×	×	×												
	3fdh						×					×				
	3ejn						×					×				
	3cgh	×		×												
	Additional templates used by:															
	Vakser	2fbn 2fi7 3gz1 3ks2 3l22 3lew 2xcc														
	Tomii	3sgh														
	Seok	3qnk														
	Huang	3ssu														
T73	3u1x	×		×				×				×				
	4jqt		×			×										
	3hbk		×				×									
	3h3l		×									×				
	Additional templates used by:															
	Vakser	1gzc 2d6k 2d6o 2xg3 2zgr 3ajz 3ap9 3o5s 3oeb														
	Xiao	3hbr														
	Tomii	3osd														
	Huang	3kb1														
T74	4k4k		×	×												
	3t2l	×			×											
	3sy6	×	×													
	Additional templates used by:															
	Vakser	1bmz 1eta 1gko 1tfp 2j71 3gf8														
	Tomii	3dgu 3ufi 4eps														
	Xiao	3gr0 4k4r														

Target	Template	Seok	Kihara	Tomii	Guerois	Zou	Vakser	Weng	Shen	Negi	Sali	Xiao	Eisenstein	Zhou	Ritchie	Elber
	Kihara	4gdu 4h40														
	Huang	1z84														
T75	4jhl		×			×		×			×	×				
	3rjt			×	×			×			×		×			
	3w7v							×			×	×				
	4jj4										×	×				
	2kxy		×	×												
	2kq1		×	×												
	Additional templates used by:															
	Vakser	1nxp 1t6f 2b4a 2gkg 2p5s 2qv0 2qxy 3a10 3dt8 3lte 3lua 3lyw														
	Xiao	2dum 2m5h 3a7f 3uf1														
	Tomii	2hsj 4fvj														
	Seok	2kql 3rtj														
	Sali	4jj6 4jko														
	Huang	3bn1 3rco														
	Negi	3t2l														
	Kihara	1z65														
T77	2kxy		×	×												
	2kq1		×	×												
	Additional templates used by:															
	Xiao	2dum 2m5h 3a7f 3uf1														
	Vakser	1t6f 3lyw														
	Tomii	4fvj														
	Seok	2kql														
	Kihara	1z65														
	Huang	3bn1														
T78	1gup		×		×			×								
	1z84	×	×													
	Additional templates used by:															
	Xiao	2p9c 3kb1 3rfu														
	Vakser	2q4h 2zw0 3bpu														
	Tomii	1hxq														
	Seok	3gup														
	Negi	4i5t														
	Kihara	2h39														
	Huang	2w83														
T79	3rco	×	×	×	×	×		×					×			
	Additional templates used by:															
	Vakser	1t6s 2h09 2od5 2p5k 2p5l 3i1g 3lpx 3a4c														
	Xiao	2gfq 4i5t 4i5v														
	Tomii	3s93														
	Huang	2b67														
T80	3dr4	×								×	×		×			
	3frk	×				×					×					
	3bn1				×	×					×					
	2fn6			×			×	×								
	4lc3	×									×					
	4k2m	×									×					
	3dr7								×		×					
	3bcx			×			×									
	3bb8	×		×												
	2oga	×													×	

Target	Template	Seok	Kihara	Tomii	Guerois	Zou	Vakser	Weng	Shen	Negi	Sali	Xiao	Eisenstein	Zhou	Ritchie	Elber
	2fnu	×			×											
	1mdx					×					×					
	Additional templates used by:															
	Vakser	1ecx 1j04 1o62 3ftb 3h14 3m0d 3ri6														
	Seok	1b9h 1o69 2c81 2po3 3b8x 3ju7 3nyt														
	Sali	1mdo 1mdz 4k2b 4k2i														
	Zou	4oca														
	Tomii	3lc3														
	Ritchie	3nyu														
	Kihara	3a7o														
	Huang	1m7o														
T81	3bbp			×	×									×		
	3bc1		×											×		
	2w83			×										×		
	2hv8	×												×		
	2gzh	×				×										
	2f9l		×	×												
	2d7c	×		×												
	1oiv	×						×								
	Additional templates used by:															
	Seok	1oiw 1yzk 2f9m 2gzd 3nmd 4c4p 4lx0														
	Vakser	1d5w 1nxp 1q21 2qxy 3e5h														
	Ritchie	3nyu 2g0n														
	Kihara	4lhw 4m8n														
	Zhou	2zet														
	Tomii	2bcg														
	Huang	2g5c														
T82	4dn2	×	×	×	×	×				×						
	4g1k	×	×	×	×					×						
	1yya	×	×		×											
	3th6					×		×								
	3k6h	×	×													
	3gvg		×				×									
	3ge6	×				×										
	2vxn	×			×											
	2i7h	×	×													
	1b9b		×	×												
	Additional templates used by:															
	Vakser	1k3e 1kly 1mss 1oia 1qqh 1x9f 2e1f 2o7g 2qff 2qt7 2wsq 2wsr 3kxq 3mhv 3pj9														
	Seok	1aw2 1mo0 2b5m 2isk 2j27 2wzv 2yc6 3bml 3gr3 3kwk 3m5k 3m9y 3ta6														
	Kihara	1btm 2oga 3bb8 3dr4 3eo8 3n2s														
	Tomii	3g14 3gfa 4g8s														
	Ritchie	4bi5 3e08														
	Huang	3ff2 3ffh														
	Zou	2b67														
	Weng	1isj														
	Shen	3ge5														
	Guerois	4bi6														
T84	4g1k	×	×	×	×					×						
	1yya	×	×		×											

Target	Template	Seok	Kihara	Tomii	Guerois	Zou	Vakser	Weng	Shen	Negi	Sali	Xiao	Eisenstein	Zhou	Ritchie	Elber
	3th6					×		×								
	3gvg		×				×									
	2vxn	×			×											
	1b9b		×	×												
	Additional templates used by:															
	Seok	1aw2 1mo0 2b5m 2j27 2yc6 3m9y 3ta6														
	Vakser	1kly 1mss 2wsq 2wsr 3kxq														
	Shen	2btm														
	Ritchie	4bi5														
	Kihara	1btm														
	Huang	3ffh														
	Guerois	4bi6														
T85	3b1f	×			×	×		×		×						
	3ggo	×	×			×	×									
	2g5c	×			×	×		×								
	3ggp		×				×								×	
	3ggg		×	×					×							
	2f1k	×				×	×									
	3ktd	×				×										
	3dzb			×		×										
	Additional templates used by:															
	Vakser	1rb4 1zik 2qdq 2vns 3llv														
	Seok	1yqg 2pv7 3gt0 3tri 4j0e														
	Ritchie	2fik														
	Huang	2ymu														
T86	3fh1		×			×		×	×							
	3ff2	×	×		×				×							
	4h3u	×	×		×											
	3i0y			×			×								×	
	2gey	×					×									
	1ohp				×		×									
	Additional templates used by:															
	Seok	2gex 3dxo 3f9s 3g0k 3g8z 3k0z 3kkg														
	Zou	1ocv 2gec 3ff3 3gzb 4mjd														
	Vakser	1gy6 1zo2 3dm8 3ov4 2z77														
	Ritchie	3hk4 3er7 43hu														
	Kihara	2bng 2k54														
	Guerois	3f7x 4hjd														
	Tomii	3fgy														
	Negi	3mjd														
	Huang	3oky														
T87	3get	×	×		×	×		×	×							
	3ffh	×	×		×					×						
	3hdo	×	×													
	3ftb	×					×									
	3euc	×	×													
	1lc5	×					×									
	Additional templates used by:															
	Seok	2gb3 3cq5 3lyl 3p1t														
	Vakser	1uu0 3ei7														
	Tomii	1b5p 3dyd														
	Ritchie	2o0r														

Target	Template	Seok	Kihara	Tomii	Guerois	Zou	Vakser	Weng	Shen	Negi	Sali	Xiao	Eisenstein	Zhou	Ritchie	Elber
	Kihara	1j32														
	Huang	2fn6														
T88	2ymu	×	×	×							×		×			
	Additional templates used by:															
	Vakser	1hzu 1sqj 2hes 2j04 2qe8 2z2n 3jrp 3rfh														
	Tomii	1r5m 2ovp 4lg9														
	Kihara	1vyh 3mkq 3ow8														
	Huang	1byr														
T89	1shy	×		×	×	×	×	×								
	4k3j	×		×			×		×							
	4fww		×	×						×						
	2uzx		×							×						
	2asu		×	×												
	Additional templates used by:															
	Kihara	3al9 3oky 3ol2														
	Tomii	4o3t 4o3u														
	Negi	1t80														
	Huang	1glp														
T90	3dr4	×	×	×	×											
	2fnu	×		×				×								
	4oca					×			×							
	4lc3	×			×											
	3bb8	×	×													
	3b8x	×	×													
	2oga				×					×						
	Additional templates used by:															
	Vakser	1ecx 1lc5 1o62 1uu0 2dou 2fn6 3bcx 3ei7 3ftb 3h14														
	Seok	1b9h 1o69 2po3 3frk 3nyt														
	Kihara	1mdo 3oga														
	Huang	2y0c														
T91	1byr	×	×	×	×		×	×	×	×						
	4ggj	×				×										
	4gel	×	×													
	1v0w		×	×												
	Additional templates used by:															
	Vakser	1v0r 2y0q 3hs2														
	Kihara	2cil 3hsi														
	Huang	2qm0														
T92	1lbk				×	×		×	×							
	2on7	×	×				×									
	2ws2	×							×							
	2on5	×	×													
	1zl9	×	×													
	1tw9	×					×									
	Additional templates used by:															
	Vakser	1gtu 1guk 1gul 1jlw 1xwg 2hsm 3w8s														
	Seok	2c3n 2c4j 2cvd 2pvq 2ycd														
	Kihara	1b48 1m0u 1yq1 2hnl														
	Tomii	1dug 3h1n 3vxw														
	Negi	2imi														
	Huang	3gsz														
T93	3g79	×	×	×	×	×	×	×	×							

Target	Template	Seok	Kihara	Tomii	Guerois	Zou	Vakser	Weng	Shen	Negi	Sali	Xiao	Eisenstein	Zhou	Ritchie	Elber
	2y0c	×	×	×	×				×							
	3gg2		×				×			×						
	4a7p	×	×													
	3vtf	×					×									
	3ojo	×		×												
	Additional templates used by:															
	Seok	1dlj 1mv8 3pid														
	Vakser	2y0d 2y0e														
	Zou	3ojl														
	Tomii	3tf5														
	Kihara	2gq4														
	Huang	3h3l														
T94	3gff	×	×	×	×	×	×	×		×						
	3q49		×							×						
	2qm0		×		×											
	Additional templates used by:															
	Vakser	2qjw 2xcc 3bjr 3ffl 3ls2														
	Kihara	1elw 3sz7 4cgw														
	Tomii	4lct														
	Shen	2ecf														
	Huang	3sy6														
	Guerois	2uz0														

Table S5 – PDB Templates used by CAPRI participants. For every target, templates used by multiple participants are listed first, followed by a list of additional templates that were only used by a single participant.

Methods used by individual participants

1	Huang	38
2	Sali	39
3	Fernandez-Fuentes	40
4	DOCK/PIERR	41
5	Grudinin	42
6	Seok	43
7	Zhou	44
8	Ritchie	45
9	Bates & SWARMDOCK	46
10	Eisenstein	47
11	Negi	48
12	Weng	49
13	Guerois	50
14	HADDOCK	51
15	Zou	53
16	Shen	54
17	Kihara & LZERD	55
18	Wade	56
19	Vakser	57
20	Tomii	58
21	Fernández-Recio	59
22	Lee	60
23	CLUSPRO	61
24	Del Carpio	62
25	Gray	63
26	Oliva	64
27	Tovchigrechko & GRAMM-X	65

Table S6 – Supplementary methods.

1 Huang

Shen-You Huang*

Research Support Computing, University of Missouri Bioinformatics Consortium, and Department of Computer Science, University of Missouri, Columbia, MO 65211, USA

* E-mail: huangshe@missouri.edu

We have predicted the oligomeric structures from individual subunits for all the CASP-CAPRI targets of Round 30 by considering the binding information from available homologs in both the individual subunit structures and the predicted complex models. Specifically, the structures of individual subunits for docking were mainly selected from the available CASP 3D structure predictions by a variety of servers including Zhang-Server [1], QUARK [2], Distill [3], 3D-Jigsaw-V5_1 [4], BAKER-ROSETTASERVER [5], RBO_Aleph [6], TASSER-VMT [7], nns [8], Atome2_CBS [9], MULTICOM-REFINE, MULTICOM-CLUSTER [10], HHPredX [11], RaptorX [12], and eThread [13], in which no severe segment clashes should be present when superimposing the subunits onto their corresponding homologous oligomers. The models for some subunits like T82 and T84 were also constructed using MODELLER [14] based on the experimentally determined homologous oligomers in the Protein Data Bank [15] if available. Then, the structures for the subunit from both the CASP predictions and the homology modeling for a target were used to construct their oligomeric structures by our in-house symmetric multimer docking program and an improved knowledge-based scoring function, respectively [16, 17]. The constructed symmetric oligomers (i.e. C_2, C_4, or D_2) from different docking runs were merged together and clustered to remove the redundancy. Last, ten models were selected for each target from the ranked binding modes by considering the binding information in the available homologous complexes. Similar method was also used to select the final models for the scoring experiments from the binding modes kindly uploaded by the CAPRI participants according to the calculated binding energy scores with our scoring function.

References

[1] J. Yang et al. "The I-TASSER Suite: protein structure and function prediction". In: *Nat. Methods* 12.1 (2015), pp. 7–8.

[2] H. Zhou et al. "Analysis of TASSER-based CASP7 protein structure prediction results". In: *Proteins* 69 Suppl 8 (2007), pp. 90–97.

[3] D. Bau et al. "Distill: a suite of web servers for the prediction of one-, two- and three-dimensional structural features of proteins". In: *BMC Bioinformatics* 7 (2006), p. 402.

[4] P. A. Bates et al. "Enhancement of protein modeling by human intervention in applying the automatic programs 3D-JIGSAW and 3D-PSSM". In: *Proteins* Suppl 5 (2001), pp. 39–46.

[5] D. E. Kim, D. Chivian, and D. Baker. "Protein structure prediction and analysis using the Robetta server". In: *Nucleic Acids Res.* 32.Web Server issue (2004), W526–531.

[6] M. Mabrouk et al. "RBO Aleph: leveraging novel information sources for protein structure prediction". In: *Nucleic Acids Res.* (2015).

[7] Y. Zhang. "Template-based modeling and free modeling by I-TASSER in CASP7". In: *Proteins* 69 Suppl 8 (2007), pp. 108–117.

[8] K. Joo et al. "Protein structure modeling for CASP10 by multiple layers of global optimization". In: *Proteins* 82 Suppl 2 (2014), pp. 188–195.

[9] J. L. Pons and G. Labesse. "@TOME-2: a new pipeline for comparative modeling of protein-ligand complexes". In: *Nucleic Acids Res.* 37.Web Server issue (2009), W485–491.

[10] J. Cheng et al. "The MULTICOM toolbox for protein structure prediction". In: *BMC Bioinformatics* 13 (2012), p. 65.

[11] J. Soding, A. Biegert, and A. N. Lupas. "The HHpred interactive server for protein homology detection and structure prediction". In: *Nucleic Acids Res.* 33.Web Server issue (2005), W244–248.

[12] M. Kallberg et al. "Template-based protein structure modeling using the RaptorX web server". In: *Nat Protoc* 7.8 (2012), pp. 1511–1522.

[13] M. Brylinski and D. Lingam. "eThread: a highly optimized machine learning-based approach to meta-threading and the modeling of protein tertiary structures". In: *PLoS ONE* 7.11 (2012), e50200.

[14] B. Webb and A. Sali. "Comparative Protein Structure Modeling Using MODELLER". In: *Curr Protoc Bioinformatics* 47 (2014), pp. 1–5.

[15] H. M. Berman et al. "The Protein Data Bank". In: *Nucleic Acids Res.* 28.1 (2000), pp. 235–242.

[16] S. Y. Huang and X. Zou. "MDockPP: A hierarchical approach for protein-protein docking and its application to CAPRI rounds 15-19". In: *Proteins* 78.15 (2010), pp. 3096–3103.

[17] S. Y. Huang and X. Zou. "An iterative knowledge-based scoring function for protein-protein recognition". In: *Proteins* 72.2 (2008), pp. 557–579.

2 Sali

Dina Schneidman-Duhovny[1,2,*] and Andrej Sali[1,2,3,◇]

[1] Department of Bioengineering and Therapeutic Sciences,
[2] Department of Pharmaceutical Chemistry,
[3] California Institute for Quantitative Biosciences (QB3),
University of California San Francisco, San Francisco, CA 94158, USA

* E-mail: dina@salilab.org
◇ E-mail: sali@salilab.org

The models of individual subunits were taken from the 150 best server models released by CASP. The 150 models were scored with the statistical potential optimized for ranking protein comparative models (soap_protein) [1] and the top 10 scoring models were selected for docking. If there was a significant variance among the top 10 models, indicating low model confidence, we did not continue to docking. The docking was performed using PatchDock[1] or SymmDock[2] for symmetric cases (C_n and D_n symmetry) [2]. The docking models were ranked using the statistical potential optimized for ranking protein-protein docking decoys (soap_pp) [1] as implemented in the IMP package[3] [3]. The docking models were clustered and the top10 cluster representatives were refined to eliminate steric clashes using FireDock[4] [4] or SymmRef [5] for symmetric cases prior to submission.

References

[1] G. Q. Dong et al. “Optimized atomic statistical potentials: assessment of protein interfaces and loops”. In: *Bioinformatics* 29.24 (2013), pp. 3158–3166.

[2] D. Schneidman-Duhovny et al. “PatchDock and SymmDock: servers for rigid and symmetric docking”. In: *Nucleic Acids Res.* 33.Web Server issue (2005), W363–367.

[3] D. Russel et al. “Putting the pieces together: integrative modeling platform software for structure determination of macromolecular assemblies”. In: *PLoS Biol.* 10.1 (2012), e1001244.

[4] N. Andrusier, R. Nussinov, and H. J. Wolfson. “FireDock: fast interaction refinement in molecular docking”. In: *Proteins* 69.1 (2007), pp. 139–159.

[5] E. Mashiach-Farkash, R. Nussinov, and H. J. Wolfson. “SymmRef: a flexible refinement method for symmetric multimers”. In: *Proteins* 79.9 (2011), pp. 2607–2623.

[1] http://bioinfo3d.cs.tau.ac.il/PatchDock/
[2] http://bioinfo3d.cs.tau.ac.il/SymmDock/
[3] http://salilab.org/imp/
[4] http://bioinfo3d.cs.tau.ac.il/FireDock/

3 Fernandez-Fuentes

Joan Segura[1] and Narcis Fernandez-Fuentes[2]

[1] GN7 of the National Institute for Bioinformatics (INB) and Biocomputing Unit, National Center of Biotechnology (CSIC), 28049 Madrid, Spain
[2] Institute of Biological, Environmental and Rural Sciences (IBERS), Aberystwyth University, SY233FG Aberystwyth, United Kingdom

* E-mail: naf4@aber.ac.uk

The strategy to derive the structural model of protein complexes build upon our original method called VORFFIP-driven dock (VD2OCK[5]) [1]. In a nutshell, VD2OCK is data-driven docking that include the prediction of protein binding sites using VORFFIP [2], the docking of monomers using PatchDock [3] and the clustering of the resulting docking space to reduce structural redundancy [4]. If the structures of individual proteins were not available, then they were predicted using our original method M4T [5] if a suitable template was available (i.e. sequence identity $>$ 40%) or Phyre [6] (otherwise). Besides the use of VORFFIP's predictions [2] to guide the docking neither further restraints nor information on homologs structures/protein complexes were included in the modeling. The selection of the top 10 representatives was done using a scoring function that combines: (i) the size of the cluster; (ii) decrease of the solvent accessibility area; (iii) the EC3DC score [7]; and (iv) PatchDock native score [3].

References

[1] J. Segura et al. "VORFFIP-driven dock: V-D2OCK, a fast and accurate protein docking strategy". In: *PLoS ONE* 10.3 (2015), e0118107.

[2] J. Segura, P. F. Jones, and N. Fernandez-Fuentes. "Improving the prediction of protein binding sites by combining heterogeneous data and Voronoi diagrams". In: *BMC Bioinformatics* 12 (2011), p. 352.

[3] D. Schneidman-Duhovny et al. "PatchDock and SymmDock: servers for rigid and symmetric docking". In: *Nucleic Acids Res.* 33.Web Server issue (2005), W363–367.

[4] D. Van Der Spoel et al. "GROMACS: fast, flexible, and free". In: *J Comput Chem* 26.16 (2005), pp. 1701–1718.

[5] N. Fernandez-Fuentes et al. "M4T: a comparative protein structure modeling server". In: *Nucleic Acids Res.* 35.Web Server issue (2007), W363–368.

[6] L. A. Kelley and M. J. Sternberg. "Protein structure prediction on the Web: a case study using the Phyre server". In: *Nat Protoc* 4.3 (2009), pp. 363–371.

[7] E. Feliu, P. Aloy, and B. Oliva. "On the analysis of protein-protein interactions via knowledge-based potentials for the prediction of protein-protein docking". In: *Protein Sci.* 20.3 (2011), pp. 529–541.

[5] http://www.bioinsilico.org/VD2OCK

4 DOCK/PIERR

Shruthi Viswanath[1,2,*] and Ron Elber[2,3,⋄]

[1] Department of Computer Science,
[2] Institute for Computational Engineering and Sciences,
[3] Department of Chemistry,
University of Texas at Austin, Austin, TX 78712, USA

* shruthi@salilab.org
⋄ ron@ices.utexas.edu

DOCK/PIERR participated in the server rounds of T68 and T69.

T68

Both monomers were modeled using the protein structure prediction server, LOOPP[6] [1]. LOOPP performs remote homology detection using a combination of sequence alignment, threading, secondary structure and exposed surface area signals, and combines all the scores using a Mathematical Programming approach. The best scoring templates are then aligned and atomic coordinates are generated in LOOPP using Modeller software [2].

The template detected by LOOPP, 1LM5_A was used for both monomers. Docking was performed by straightforward docking using DOCK/PIERR server[7] [3, 4, 5]. The search for conformations is performed using FFT with a Van der Waals score and a residue potential, PIE [4, 6]. This is followed by side-chain remodeling, energy minimization and reranking using a combination of residue (PIE) and atomic (PISA) potentials [5]. The top 10 models from scoring were submitted as is.

T69

Monomers were modeled using LOOPP server as well as by separately selecting a template from PSI-BLAST [7] and generating monomers from Modeller, selecting the model with the best DOPE score in the top 5 models. In both cases, LOOPP and Modeller, the template 1QLW was chosen.

The monomers from LOOPP as well as those from Modeller were modeled in two independent docking runs. DOCK/PIERR server was used for docking, as in T68, above. The difference here is that for final reranking, a combination of residue (PIE), atomic (PISA) and hydrogen bond (HB) potentials were used. All these potentials are available here[8].

9 Of the top 10 submitted models were from the run of LOOPP-modeled monomers on DOCK/PIERR. The tenth model was selected to be similar to the homologous complex, 1QLW, from the run of Modeller-modeled monomers on DOCK/PIERR.

References

[1] B. K. Vallat et al. “Building and assessing atomic models of proteins from structural templates: learning and benchmarks”. In: *Proteins* 76.4 (2009), pp. 930–945.

[2] A. Sali and T. L. Blundell. “Comparative protein modelling by satisfaction of spatial restraints”. In: *J. Mol. Biol.* 234.3 (1993), pp. 779–815.

[3] S. Viswanath, D. V. Ravikant, and R. Elber. “DOCK/PIERR: web server for structure prediction of protein-protein complexes”. In: *Methods Mol. Biol.* 1137 (2014), pp. 199–207.

[4] D. V. Ravikant and R. Elber. “Energy design for protein-protein interactions”. In: *J Chem Phys* 135.6 (2011), p. 065102.

[5] S. Viswanath, D. V. Ravikant, and R. Elber. “Improving ranking of models for protein complexes with side chain modeling and atomic potentials”. In: *Proteins* 81.4 (2013), pp. 592–606.

[6] D. V. Ravikant and R. Elber. “PIE-efficient filters and coarse grained potentials for unbound protein-protein docking”. In: *Proteins* 78.2 (2010), pp. 400–419.

[7] S. F. Altschul et al. “Gapped BLAST and PSI-BLAST: a new generation of protein database search programs”. In: *Nucleic Acids Res.* 25.17 (1997), pp. 3389–3402.

[6] http://clsb.ices.utexas.edu/web/loopp.html
[7] http://clsb.ices.utexas.edu/web/dock.html
[8] http://clsb.ices.utexas.edu/web/dock_details.html

5 Grudinin

Sergei Grudinin[1,2,*], Petr Popov[1,2,3] and Emilie Neveu[1,2]

[1] LJK, Université Grenoble Alpes, CNRS, 38000 Grenoble, France
[2] INRIA, 38000 Grenoble, France
[3] Moscow Institute of Physics and Technology, Dolgoprudniy, Russia

* E-mail: sergei.grudinin@inria.fr

A multi-resolution algorithm for docking symmetric proteins and its performance in CAPRI Round 30.

Most of the targets in Round 30 of CAPRI were homodimers and homotetramers, thus it was a good opportunity to test our novel symmetry assembling docking method [1]. To do so, we imposed C_2 symmetry constraints for all the homodimers and we imposed C_4 and D_2 symmetry constraints for all the homotetramers from the target complexes. Below, we present the new fast multi-resolution method for docking both symmetric and non-symmetric protein complexes that was used in Round 30 of CAPRI.

First, the structures of the individual subunits were taken from the stage two predictions of the CASP10 assessment experiment. More precisely, starting from 150 available CASP 3D models of monomers, we predicted models of symmetric multimers using the novel symmetry docking method, which performs symmetry-induced protein docking using the shape-complementarity scoring function computed as spherical polar Fourier correlations [1]. Specifically, this method performs exhaustive search over the available (four in case of cyclic symmetries or six otherwise) degrees of freedom for the given point group symmetry type. For the targets of Round 30 of CAPRI we imposed three types of symmetry, C_2, C_4, and D_2. For the case of heterodimers, we used the standard Hex docking method [2].

For the input of the docking methods, we generated the scaffolds of initial models of monomers by “cutting-off” the side chains. More specifically, we mutated all side-chains except for the glycines to alanines. Compared to the standard all-atom rigid-body docking methods, we expect the scaffold docking approach to produce binding poses that are less sensitive to the flexibility of the side-chains. We clustered the solutions with the threshold ligand-RMSD value of 8 Å using the RigidRMSD library [3]. Finally, we ranked the clusters by the value of the best score and kept 50 best clusters for the refinement stage. In total, for each target we proceeded to the refinement with 7,500 modeled structures of protein complexes.

On the next step, we optimized each putative binding interface of the all-atom representation of a protein complex by means of a rigid-body first-order minimization scheme [4] as implemented in our software package SAMSON[9]. Specifically, after each rigid-body minimization step we proceeded with the optimization of side-chains described by the rotameric representation using the SCWRL4 package [5]. We computed the interactions between the subunits in a protein complex using the novel reference state-free knowledge-based scoring function KSENIA [6], which is smooth by construction and is thus very suitable for a gradient-based minimization protocol. Finally, we ranked the predictions by the value of the KSENIA potential of the optimized structure and selected ten best candidates for the submission.

References

[1] D. W. Ritchie and S. Grudinin. “Some Recent Progress on Generating Symmetric Protein Complexes Using Spherical Polar Fourier Docking Correlations”. In: *Proceedings of the 14th Open Days in Biology, Computer Science and Mathematics (JOBIM)*. Toulouse (France). 2013.

[2] D. W. Ritchie and G. J. Kemp. “Protein docking using spherical polar Fourier correlations”. In: *Proteins* 39.2 (2000), pp. 178–194.

[3] P. Popov and S. Grudinin. “Rapid determination of RMSDs corresponding to macromolecular rigid body motions”. In: *J Comput Chem* 35.12 (2014), pp. 950–956.

[4] P. Popov, S. Redon, and S. Grudinin. “Carbon: Controlled-avancement rigid-body optimization of nanosystems”. In: (). Unpublished.

[5] G. G. Krivov, M. V. Shapovalov, and R. L. Dunbrack. “Improved prediction of protein side-chain conformations with SCWRL4”. In: *Proteins* 77.4 (2009), pp. 778–795.

[6] P. Popov and S. Grudinin. “Knowledge of Native Protein-Protein Interfaces is Sufficient to Construct Predictive Models for the Selection of Binding Candidates”. In: (). Unpublished.

[9] https://team.inria.fr/nano-d/software/samson

6 Seok

Hasup Lee, Minkyung Baek, Sangwoo Park, Lim Heo, Gyu Rie Lee and Chaok Seok*

Department of Chemistry, Seoul National University, Seoul 151-747, Republic of Korea

* E-mail: chaok@snu.ac.kr

Prediction of Protein-Protein Interactions by GALAXY in CAPRI Round 30

Recently developed features of the GALAXY protein modeling package including template-based structure prediction, loop modeling, model refinement, and protein-protein docking were used to predict the structures of protein complexes from amino acid sequences in CAPRI Round 30. Template-based complex structure prediction was applied to 22 homomer and 2 heteromer targets, and ab initio docking was carried out for 1 target (T88). In template-based prediction, up to 10 structural templates were first selected by using GalaxyGemini [1] for homomer targets and by using HHsearch [2] for heteromer targets. 3D models for the whole complex were then constructed from the templates by the model-building components of GalaxyTBM [3, 4] after deleting predicted signaling peptide segments. Unreliably modeled regions were then detected and re-modeled by using GalaxyLoop [5, 6]. The resulting models were further refined using a new version of GalaxyRefine [7] modified for complex structure refinement. Symmetry restraints were applied to homomer targets during the loop modeling and refinement procedures. For ab initio docking of the two subunits of T88, the GalaxyPPDock [8] method that performs global optimization of a new hybrid protein interaction energy function that is composed of physics-based and knowledge-based energy components was employed. The final models obtained by docking were also refined by GalaxyRefine. When the CAPRI criterion was used to evaluate the models for the 16 targets with released PDB structures as of Oct 31, 2014, 5 targets were predicted with medium quality and 4 targets with acceptable quality. Contributions of the interface loops modelled by GalaxyLoop to the fraction of native contact f(nat) turned out to be significant [+0.15 (T69), +0.19 (T80), +0.28 (T85), and +0.17 (T87)]. Refinement by GalaxyRefine improved ligand RMSD (from 6.15 Å to 5.96 Å), interface RMSD (from 3.17 Å to 3.14 Å), f(nat) (from 0.50 to 0.54), and MolProbity score (from 3.29 to 2.65) when averaged over the 9 targets predicted with at least acceptable quality. In the scoring round of CAPRI 30, models were scored by GalaxyRefine energy function that consists of molecular mechanics energy terms, generalized born solvation free energy, distance- and orientation-dependent statistical potential, statistical hydrogen bonding energy, etc after local energy minimization. Medium and acceptable quality models were selected for 6 and 3 targets, respectively out of the 16 evaluated targets.

References

[1] H. Lee et al. “GalaxyGemini: a web server for protein homo-oligomer structure prediction based on similarity”. In: *Bioinformatics* 29.8 (2013), pp. 1078–1080.

[2] J. Soding. “Protein homology detection by HMM-HMM comparison”. In: *Bioinformatics* 21.7 (2005), pp. 951–960.

[3] J. Ko, H. Park, and C. Seok. “GalaxyTBM: template-based modeling by building a reliable core and refining unreliable local regions”. In: *BMC Bioinformatics* 13 (2012), p. 198.

[4] J. Ko et al. “GalaxyWEB server for protein structure prediction and refinement”. In: *Nucleic Acids Res.* 40.Web Server issue (2012), W294–297.

[5] H. Park et al. “Refinement of protein termini in template-based modeling using conformational space annealing”. In: *Proteins* 79.9 (2011), pp. 2725–2734.

[6] H. Park and C. Seok. “Refinement of unreliable local regions in template-based protein models”. In: *Proteins* 80.8 (2012), pp. 1974–1986.

[7] L. Heo, H. Park, and C. Seok. “GalaxyRefine: Protein structure refinement driven by side-chain repacking”. In: *Nucleic Acids Res.* 41.Web Server issue (2013), W384–388.

[8] H. Lee et al. “GalaxyPPDock: Protein-protein docking by cluster-guided conformational space annealing”. In: (). In preparation.

7 Zhou

Sanbo Qin and Huan-Xiang Zhou*

Department of Physics and Institute of Molecular Biophysics, Florida State University, Tallahassee, FL 32306, USA

* E-mail: hzhou4@fsu.edu

Oligomer model selection using predicted protein interfaces

For the monomers, we used CASP 3D structure predictions stage2 server models (mostly from Zhang-Server or BAKER-ROSETTASEVER), except in a few targets where we also used models from the HADDOCK team (Txx_P20). For each target, usually two monomer models were included, but occasionally one or three models were included instead. We then used M-ZDOCK [1] to build oligomers with symmetry constraints for nearly all targets, and ZDOCK [2] and homology modeling for two cases without symmetry. The oligomer models were clustered by a clustering program from HADDOCK [3], with Cα RMSD cutoff at 5 Å (or at 10 Å if too few clusters with at least 10 members were produced). The clusters starting from different monomer models were compared and combined if overlap occurred. The latter combined clusters were given preference in final selection. From each cluster, two models were retained; one was the cluster center and the other had the best ZDOCK score. All retained models were ranked according to agreement with protein interface predictions by our meta-PPISP server [4]. The final 10 oligomer models were energy minimized by Chiron [5] or AMBER [6] before submission.

References

[1] B. Pierce, W. Tong, and Z. Weng. "M-ZDOCK: a grid-based approach for Cn symmetric multimer docking". In: *Bioinformatics* 21.8 (2005), pp. 1472–1478.

[2] R. Chen, L. Li, and Z. Weng. "ZDOCK: an initial-stage protein-docking algorithm". In: *Proteins* 52.1 (2003), pp. 80–87.

[3] C. Dominguez, R. Boelens, and A. M. Bonvin. "HADDOCK: a protein-protein docking approach based on biochemical or biophysical information". In: *J. Am. Chem. Soc.* 125.7 (2003), pp. 1731–1737.

[4] S. Qin and H. X. Zhou. "meta-PPISP: a meta web server for protein-protein interaction site prediction". In: *Bioinformatics* 23.24 (2007), pp. 3386–3387.

[5] S. Ramachandran et al. "Automated minimization of steric clashes in protein structures". In: *Proteins* 79.1 (2011), pp. 261–270.

[6] D. A. Case et al. "The Amber biomolecular simulation programs". In: *J Comput Chem* 26.16 (2005), pp. 1668–1688.

8 Ritchie

David W. Ritchie[1,*], Bernard Maigret[2], Marie-Dominique Devignes[2] and Anisah Ghoorah[3]

[1] INRIA Nancy – Grand Est, 54600 Villers-les-Nancy, France
[2] CNRS, LORIA, Campus Scientifique, BP 239, 54506 Vandoeuvre-les-Nancy, France
[3] Department of Computer Science and Engineering, University of Mauritius, Reduit, Mauritius

[*] E-mail: dave.ritchie@inria.fr

Docking the Round 30 CASP/CAPRI Targets Using CASP Consensus Structures and Symmetry-Constrained Polar Fourier Docking Correlations

Many of the targets in Round 30 of CAPRI were predicted by the CASP organisers to be C_2 symmetric homo-dimers, and were initially presented to the CASP community as fold prediction targets. Because many good models were produced by several CASP predictor groups (i.e. were reported as being within 3 Å RMS of the crystal structure by the CASP assessors), we decided to base our docking predictions on only those models. In other words, for each target that we attempted to dock, we downloaded and examined the best "stage2" monomer models from the CASP prediction web site[10]. However, due to time constraints, we attempted to dock only targets T79, T80, T81, T82, T84, T85, T86, T88, T89, and T90.

For each target attempted, we used our "Kpax" protein structure alignment program [1] to select a representative, or "consensus", structure from the provided "stage2" models. We first calculated all-versus-all structure alignments of the CASP models to produce a square matrix of normalised Kpax similarity scores. We then selected the structure from this matrix which had the greatest structural similarity (i.e. row-wise total similarity score) to all other structures, and we treated that structure as a single consensus structure for docking.

In order to check for possible structural homologues, we then used our KBDOCK web server [2] to search for Pfam families which involved the given target domain(s) and which had examples of structural inter- or intra- chain homo-dimers in the PDB. This procedure found candidate templates for T80, T82, T84, T85, T86, and T88, which were then used to orient the target monomers by least-squares superposition. We also used our novel "SAM" (Symmetry Assembler) docking program (manuscript in preparation) to perform four-dimensional FFT docking searches with built-in C_2 symmetry constraints. More specifically, SAM performs a brute-force search correlation search over the available degrees of freedom for the given point group symmetry type. For example, for C_2 symmetry, SAM searches over one translational and three rotational degrees of freedom. SAM uses essentially the same polar FFT code as Hex [3], but with knowledge of the target symmetry embedded directly into the correlation equations. Thus, every candidate docking orientation produced by SAM is guaranteed to have precisely the desired symmetry. The list of solutions produced by SAM were then clustered using a similarity threshold of 6 Å RMSD in order to remove near duplicate solutions, and the top-scoring members of each cluster were assessed by eye to eliminate solutions which seemed improbable. If one or more possible homology templates were found by KBDOCK these were taken as our first predictions, and the remaining (from 7 to 9) predictions were taken from the SAM clusters. For targets T79, T87, T89 and T90, all 10 predictions were calculated by SAM.

References

[1] D. W. Ritchie et al. "Fast protein structure alignment using Gaussian overlap scoring of backbone peptide fragment similarity". In: *Bioinformatics* 28.24 (2012), pp. 3274–3281.

[2] A. W. Ghoorah et al. "KBDOCK 2013: a spatial classification of 3D protein domain family interactions". In: *Nucleic Acids Res.* 42.Database issue (2014), pp. D389–395.

[3] D. W. Ritchie and G. J. Kemp. "Protein docking using spherical polar Fourier correlations". In: *Proteins* 39.2 (2000), pp. 178–194.

[10] http://predictioncenter.org/download_area/CASP11/server_predictions/

9 Bates & SWARMDOCK

Mieczyslaw Torchala, Raphaël A. G. Chaleil and Paul A. Bates*

Biomolecular Modelling Laboratory, The Francis Crick Institute, Lincoln's Inn Fields Laboratory, London WC2A 3LY, United Kingdom

* E-mail: paul.bates@crick.ac.uk

A protocol for Docking Homo-Multimers and Filtering Solutions According to Symmetry Operators

Monomer units in a homo-multimer complex are usually related to each other by one or more axis of symmetry, a homodimer for example by a two fold rotation axis. There are two ways in which symmetry can be accounted for in protein docking, either to restrict the initial search space, and therefore symmetry operators are embedded within the docking algorithm itself, or as a post-docking filter. Here we use the latter methodology.

Methods

For all docking runs, for both the automatic and manual submissions, we used our publicly available[11] flexible docking server, SwarmDock [1, 2]. This server does not take account of any potential symmetry between the protein structures to be docked and can only consider two proteins at a time, one receptor and one ligand pair. For the automatic predictions the protein monomers were built with our fully automatic, publicly available[12] protein structure prediction server, 3D-JIGSAW, which employs a genetic algorithm to recombine models built from numerous templates [3]. For our manual submissions, we used the best-input structure from the CASP 150 set, ranked according to our automatic CASP11 quality assessment server, OccuScore; this automatic server calculates DDFIRE [4] and TM scores [5] for each structure, then structures are ordered by DDFIRE values and hierarchical clustered using TM scores (TM threshold of 0.5).

Where close homologs to the complex to be modeled exist, the modeled monomer could be superimposed onto the monomer units of the homologs, thereby potential residue contacts across the symmetry axis of each interface could be obtained. For such cases, the restrained docking mode of the SwarmDock server was used. However, such constraints may be considered to be weak and only partially restrict the full search space. In all other cases full blind docking was performed. In the case of modeling tetramers a two-step docking protocol was performed; the highest ranked dimer was chosen from the first docking run and this solution was subsequently docked against itself to obtain a tetramer. For a trimer, first a receptor ligand pair was docked, solutions scored according to the existence of two monomers being related by a three-fold axis, then the top ranked solution was docked against another copy of the monomer.

The solutions were ranked and clustered as described in [1, 2]. In addition, a new filter was created based on the expected symmetry operators relating monomers (C_2 for dimers, C_3 for trimers and C_4 or D_2 for tetramers). Additional information used to choose the best 10 structures were binding energies, size of clusters and distribution of contacts. Interestingly, for manual submissions, inspection of the final ranked list of solutions sometimes indicated that a particularly favorable binding energy or cluster size could override the lack of symmetry between monomer units. For such cases, non-symmetric homo-multimer complexes were submitted.

Availability

Protein structure modeling server: http://bmm.crick.ac.uk/~populus
Protein-Protein docking server: http://bmm.crick.ac.uk/~SwarmDock

References

[1] M. Torchala et al. "SwarmDock: a server for flexible protein-protein docking". In: *Bioinformatics* 29.6 (2013), pp. 807–809.

[2] M. Torchala et al. "A Markov-chain model description of binding funnels to enhance the ranking of docked solutions". In: *Proteins* 81.12 (2013), pp. 2143–2149.

[3] M. N. Offman, A. L. Tournier, and P. A. Bates. "Alternating evolutionary pressure in a genetic algorithm facilitates protein model selection". In: *BMC Struct. Biol.* 8 (2008), p. 34.

[4] Y. Yang and Y. Zhou. "Specific interactions for ab initio folding of protein terminal regions with secondary structures". In: *Proteins* 72.2 (2008), pp. 793–803.

[5] Y. Zhang and J. Skolnick. "Scoring function for automated assessment of protein structure template quality". In: *Proteins* 57.4 (2004), pp. 702–710.

[11] http://bmm.crick.ac.uk/~SwarmDock
[12] http://bmm.crick.ac.uk/~populus/

10 Eisenstein

Efrat Ben-Zeev[1] and Miriam Eisenstein[2,*]

[1] G-INCPM,
[2] Department of Chemical Research Support,
Weizmann Institute of Science, Rehovot, 7610001, Israel

* E-mail: Miriam.Eisenstein@weizmann.ac.il

Prediction of structures in the CAPRI-30/CASP-11 experiment

Molecular modeling of the monomers

Modeling templates were identified by sequence alignment using BLAST [1]. The alignments were manually adjusted by shifting inserts/deletions in secondary structure elements to the ends of such elements or to predicted loop regions. Conservation and similarity of residues in expected dimerization interfaces was taken into consideration in the alignment.

Model building

Homology models were constructed using Modeller [2]. We did not use models from the CASP 3D structure predictions.

Oligomer construction

Modeling of homodimers was based on the dimerization mode of the template provided significant conservation and similarity of interface residues was detected. In some cases several dimerization modes were considered. Models were constructed by superposing the modeled monomers onto the template and energy minimized using Discovery Studio (Dassault Systèmes BIOVIA, Discovery Studio Modeling Environment, San Diego: Dassault Systèmes, 2015).

Docking

In cases when the modeling template was not a dimer we docked the molecules using the geometric-electrostatic-hydrophobic version of MolFit [3, 4, 5] followed by a propensity and solvation based post-scan filter [6].

References

[1] S. F. Altschul et al. “Basic local alignment search tool”. In: *J. Mol. Biol.* 215.3 (1990), pp. 403–410.

[2] A. Sali and T. L. Blundell. “Comparative protein modelling by satisfaction of spatial restraints”. In: *J. Mol. Biol.* 234.3 (1993), pp. 779–815.

[3] E. Katchalski-Katzir et al. “Molecular surface recognition: determination of geometric fit between proteins and their ligands by correlation techniques”. In: *Proc. Natl. Acad. Sci. U.S.A.* 89.6 (1992), pp. 2195–2199.

[4] A. Heifetz, E. Katchalski-Katzir, and M. Eisenstein. “Electrostatics in protein-protein docking”. In: *Protein Sci.* 11.3 (2002), pp. 571–587.

[5] A. Berchanski, B. Shapira, and M. Eisenstein. “Hydrophobic complementarity in protein-protein docking”. In: *Proteins* 56.1 (2004), pp. 130–142.

[6] N. Kowalsman and M. Eisenstein. “Combining interface core and whole interface descriptors in postscan processing of protein-protein docking models”. In: *Proteins* 77.2 (2009), pp. 297–318.

11 Negi

Surendra S. Negi*

Sealy Center for Structural Biology and Molecular Biophysics, University of Texas Medical Branch, 301 University Boulevard, Galveston, TX 77555-0857, USA

* E-mail: ssnegi@utmb.edu

Since the structural information of the given target proteins in CAPRI round 30 were not available in the protein data bank [1], the accuracy of the modeled protein complexes were dependent on: a) selection of the template structures, and b) model structure of the target protein. In our approach, all the target sequences were submitted to HHPRED web server [2] to find the best homologous template structures. The sequence alignment between the target sequence and the template structure was manually checked to adjust the gap positions. ROBETTA web server [3] and modeller program [4] were used to generate the preliminary model structures for the target proteins T68-T72 and T73-T94, respectively. Further, molecular docking programs HEX [5] and S-DOCK were used to generate an initial set of docked protein complexes. Using default parameters, a set of 100 docked structures were generated and the RMSD values between docked structures were calculated using profit program [6]. The docked structures with RMSD values less than 5 Å were clustered using Cytoscape program [7]. Finally, a representative structure from each of the top ten clusters were deposited to the CAPRI website.

References

[1] H. Berman, K. Henrick, and H. Nakamura. “Announcing the worldwide Protein Data Bank”. In: *Nat. Struct. Biol.* 10.12 (2003), p. 980.

[2] J. Soding, A. Biegert, and A. N. Lupas. “The HHpred interactive server for protein homology detection and structure prediction”. In: *Nucleic Acids Res.* 33.Web Server issue (2005), W244–248.

[3] D. E. Kim, D. Chivian, and D. Baker. “Protein structure prediction and analysis using the Robetta server”. In: *Nucleic Acids Res.* 32.Web Server issue (2004), W526–531.

[4] A. Sali and T. L. Blundell. “Comparative protein modelling by satisfaction of spatial restraints”. In: *J. Mol. Biol.* 234.3 (1993), pp. 779–815.

[5] D. W. Ritchie and G. J. L. Kemp. “Fast computation, rotation, and comparison of low resolution spherical harmonic molecular surfaces”. In: *J. Comput. Chem.* 20.4 (1999), pp. 383–395.

[6] A. D. McLachlan. “Rapid comparison of protein structures”. In: *Acta Cryst.* A38 (1982), pp. 871–873.

[7] M. E. Smoot et al. “Cytoscape 2.8: new features for data integration and network visualization”. In: *Bioinformatics* 27.3 (2011), pp. 431–432.

12 Weng

Zhiping Weng*, Thom Vreven, Brian G. Pierce and Tyler M. Borrman†

Program in Bioinformatics and Integrative Biology, University of Massachusetts Medical School, Worcester, MA 01605, USA

† Current address: Institute for Bioscience and Biotechnology Research, University of Maryland, Rockville, MD 20850, USA

* E-mail: Zhiping.Weng@umassmed.edu

For the structures of the component proteins, we used the 3D models provided by CASP, specifically those from the Zhang server and the Baker-Rosetta server. We visually inspected the two sets of five structures from these servers, identifying consensus folds, and selected structures with minimally exposed flexible termini that could obscure potential binding sites. Docking was performed using one or two of the structures from these sets.

Our complex structure prediction was started with literature searches for biological information that could be used to restrict the docking search space. We generally found less information versus previous CAPRI rounds. For a few cases we found homologous proteins in the Protein Data Bank that were either listed as a homodimer, or had two copies in the asymmetric unit that could possibly represent a homodimer. In such cases we superimposed the component protein onto the PDB template and included it as one of our ten predictions.

We used our ZDOCK series of programs to generate the complex structures [1, 2, 3, 4, 5, 6, 7]. Homodimers and trimers were docked using our algorithm M-ZDOCK that was designed for symmetric multimers [6] imposing C_2 or C_3 symmetry as appropriate for the given target. Homotetramers were docked using M-ZDOCK, both in a single step with C_4 symmetry and in a two-step procedure as a dimer of dimers for D_2 symmetry. Heterodimers were docked using ZDOCK version 3.0.2 [5, 7]. The docking predictions were then pruned using our distance-based clustering algorithm [8, 9] and final predictions were selected based on a combination of ZDOCK score, prediction density, and manual inspection. For the scoring rounds we applied the IRAD reranking function [10], and used a combination of IRAD score and manual inspection for selecting the final ten predictions.

References

[1] R. Chen, L. Li, and Z. Weng. “ZDOCK: an initial-stage protein-docking algorithm”. In: *Proteins* 52.1 (2003), pp. 80–87.

[2] T. Vreven et al. “Performance of ZDOCK in CAPRI rounds 20-26”. In: *Proteins* (2013).

[3] R. Chen and Z. Weng. “A novel shape complementarity scoring function for protein-protein docking”. In: *Proteins* 51.3 (2003), pp. 397–408.

[4] B. G. Pierce et al. “ZDOCK server: interactive docking prediction of protein-protein complexes and symmetric multimers”. In: *Bioinformatics* 30.12 (2014), pp. 1771–1773.

[5] J. Mintseris et al. “Integrating statistical pair potentials into protein complex prediction”. In: *Proteins* 69.3 (2007), pp. 511–520.

[6] B. Pierce, W. Tong, and Z. Weng. “M-ZDOCK: a grid-based approach for Cn symmetric multimer docking”. In: *Bioinformatics* 21.8 (2005), pp. 1472–1478.

[7] B. G. Pierce, Y. Hourai, and Z. Weng. “Accelerating protein docking in ZDOCK using an advanced 3D convolution library”. In: *PLoS ONE* 6.9 (2011), e24657.

[8] T. Vreven, H. Hwang, and Z. Weng. “Exploring angular distance in protein-protein docking algorithms”. In: *PLoS ONE* 8.2 (2013), e56645.

[9] H. Hwang et al. “Performance of ZDOCK and ZRANK in CAPRI rounds 13-19”. In: *Proteins* 78.15 (2010), pp. 3104–3110.

[10] T. Vreven, H. Hwang, and Z. Weng. “Integrating atom-based and residue-based scoring functions for protein-protein docking”. In: *Protein Sci.* 20.9 (2011), pp. 1576–1586.

13 Guerois

Jinchao Yu, Françoise Ochsenbein and Raphäel Guerois*

Institute for Integrative Biology of the Cell (I2BC), Commissariat à l'Energie Atomique et aux Energies Alternatives (CEA), Centre National de la Recherche Scientifique (CNRS), Université Paris-Saclay, CEA-Saclay, F-91191 Gif-sur-Yvette, France

* E-mail: Raphael.Guerois@cea.fr

The structures of the individual subunits were obtained from template based modeling using the hits obtained by HHsearch [1] as reference structures. When several templates were available, up to five models were generated from every single template but not from multiple templates. Overall, for 80% of the cases, we used directly BAKER-Rosetta [2] and/or Zhang-server [3] models. When we didn't use the models provided by the CASP servers, swiss-model server [4] was used for sequence identities above 30% and Rosetta3.5 [2] for lower sequence identities. Models generated with Rosetta were generally constrained during the relax step so they did not diverge much from the initial Calpha coordinates. Loops were sampled using the Rosetta kic module [5] with specific constraints to sample conformations of interest. When possible, we used comparative modelling to generate the models of the assemblies. The different orientations observed in the multimeric assembly of different members of a superfamily were used as initial seeds. In 13 out of 25 cases, we also used template free docking using Zdock [6] and Mzdock (for symmetry constraints) [7] to generate rigid-body initial models. Convergence between template-based and template-free approaches strengthened the confidence for a specific orientation which was further sampled. Downstream template-free rigid-body docking, the most likely decoys were identified using a composite score built from InterEvScore [8], Soap_pp [9] and Zrank [10]. Rosetta3.5 was used to relax the selected models under symmetry constraints [11]. The fcc program was used for clustering [12].

References

[1] J. Soding. "Protein homology detection by HMM-HMM comparison". In: *Bioinformatics* 21.7 (2005), pp. 951–960.

[2] R. Das and D. Baker. "Macromolecular modeling with rosetta". In: *Annu. Rev. Biochem.* 77 (2008), pp. 363–382.

[3] Y. Zhang. "I-TASSER: fully automated protein structure prediction in CASP8". In: *Proteins* 77 Suppl 9 (2009), pp. 100–113.

[4] M. Biasini et al. "SWISS-MODEL: modelling protein tertiary and quaternary structure using evolutionary information". In: *Nucleic Acids Res.* 42.Web Server issue (2014), W252–258.

[5] D. J. Mandell, E. A. Coutsias, and T. Kortemme. "Subangstrom accuracy in protein loop reconstruction by robotics-inspired conformational sampling". In: *Nat. Methods* 6.8 (2009), pp. 551–552.

[6] R. Chen, L. Li, and Z. Weng. "ZDOCK: an initial-stage protein-docking algorithm". In: *Proteins* 52.1 (2003), pp. 80–87.

[7] B. Pierce, W. Tong, and Z. Weng. "M-ZDOCK: a grid-based approach for Cn symmetric multimer docking". In: *Bioinformatics* 21.8 (2005), pp. 1472–1478.

[8] J. Andreani, G. Faure, and R. Guerois. "InterEvScore: a novel coarse-grained interface scoring function using a multi-body statistical potential coupled to evolution". In: *Bioinformatics* 29.14 (2013), pp. 1742–1749.

[9] G. Q. Dong et al. "Optimized atomic statistical potentials: assessment of protein interfaces and loops". In: *Bioinformatics* 29.24 (2013), pp. 3158–3166.

[10] B. Pierce and Z. Weng. "ZRANK: reranking protein docking predictions with an optimized energy function". In: *Proteins* 67.4 (2007), pp. 1078–1086.

[11] I. Andre et al. "Emergence of symmetry in homooligomeric biological assemblies". In: *Proc. Natl. Acad. Sci. U.S.A.* 105.42 (2008), pp. 16148–16152.

[12] J. P. Rodrigues et al. "Clustering biomolecular complexes by residue contacts similarity". In: *Proteins* 80.7 (2012), pp. 1810–1817.

14 HADDOCK

Anna Vangone, João P. G. L. M. Rodrigues, Gydo van Zundert, Mehdi Nellen, Li Xue, Ezgi Karaca, Adrien S. J. Melquiond, Koen Visscher, Panagiotis L. Kastritis and Alexandre M. J. J. Bonvin*

Bijvoet Center for Biomolecular Research, Faculty of Science – Chemistry, Utrecht University, Padualaan 8, 3584CH, Utrecht, The Netherlands

* E-mail: a.m.j.j.bonvin@uu.nl

HADDOCK in CASP-CAPRI ROUND 30

In this CAPRI round, the HADDOCK group generated models for each target using a combination of homology modeling/threading and docking/refinement with the HADDOCK web server [1] (submissions were only done for the server category of CAPRI). Most of our predictions started from a model built with MODELLER (v9.12) [2] using HHpred [3] for template search, or I-TASSER [4], or models generated using both methods. The target/template alignment was optimized, if necessary, either with NEEDLE [5] in case of high sequence identity, or using manually-curated multiple sequence alignments. In one particular case, T77, we created a model of the monomer using a flexible multibody docking approach [6] due to the disposition of the individual domains. Typically, an ensemble of the ten best monomer models was used as starting point for HADDOCK. Since all targets were homo-oligomeric structures, non-crystallography symmetry and regular symmetry restraints were used [6] in combination with center-of-mass restraints (ab initio mode of HADDOCK). In cases where homologous complexes were identified, Ca-Ca distance restraints were applied, derived either manually or determined by PS-HOMPPI [7]. A number of targets were also directly modeled as a multimer in MODELLER and only refined in explicit solvent in HADDOCK. The models for submission were distributed over the clusters ranked by HADDOCK. A summary of the modelling methodology and restraints used is provided in the following supplementary table.

References

[1] S. J. de Vries, M. van Dijk, and A. M. Bonvin. "The HADDOCK web server for data-driven biomolecular docking". In: *Nat Protoc* 5.5 (2010), pp. 883–897.

[2] A. Sali and T. L. Blundell. "Comparative protein modelling by satisfaction of spatial restraints". In: *J. Mol. Biol.* 234.3 (1993), pp. 779–815.

[3] A. Hildebrand et al. "Fast and accurate automatic structure prediction with HHpred". In: *Proteins* 77 Suppl 9 (2009), pp. 128–132.

[4] A. Roy, A. Kucukural, and Y. Zhang. "I-TASSER: a unified platform for automated protein structure and function prediction". In: *Nat Protoc* 5.4 (2010), pp. 725–738.

[5] H. McWilliam et al. "Analysis Tool Web Services from the EMBL-EBI". In: *Nucleic Acids Res.* 41.Web Server issue (2013), pp. 597–600.

[6] E. Karaca et al. "Building macromolecular assemblies by information-driven docking: introducing the HADDOCK multibody docking server". In: *Mol. Cell Proteomics* 9.8 (2010), pp. 1784–1794.

[7] L. C. Xue, D. Dobbs, and V. Honavar. "HomPPI: a class of sequence homology based protein-protein interface prediction methods". In: *BMC Bioinformatics* 12 (2011), p. 244.

Target ID						
CAPRI	CASP	Alignment	Template	Modeling	Docking	Restraints
T68	T0759	HHpred	1LM5, 1LM7	Modeller	HADDOCK	Ca-Ca + Symmetry
T69	T0764	HHpred + Needle	1QLW	Modeller	HADDOCK	Ca-Ca + Symmetry
T70	T0765	-	-	I-Tasser	HADDOCK	Symmetry + CM
T71	T0768	HHpred	4GT6, 2Z80[a]	Modeller	HADDOCK	Symmetry + CM
T72	T0770	MSAProbs	3EJN, 3FDH, 3MX3	Modeller	HADDOCK	Symmetry + CM
T73	T0772	HHpred	3HBK	Modeller	HADDOCK	Symmetry + CM
T74	T0774	HHpred	4K4K	Modeller	HADDOCK	Symmetry + CM
T75	T0776	HHpred + Needle	4JHL	Modeller	HADDOCK	Symmetry + AIRs (Homology)
T77	T0780	HHpred	2KQ1	Modeller + HADDOCK[b]	HADDOCK	Symmetry + CM
T78	T0781	HHpred	1GUP	Modeller	HADDOCK	Symmetry + CM
T79	T0782	HHpred	3RCO	Modeller	HADDOCK	Symmetry + CM
T80	T0786	HHpred + Needle	1MDO	Modeller	HADDOCK (Refinement)	Symmetry + Ca-Ca (PS-HOMPPI)
T81	T0797					
	T0798	HHpred + Needle	2GZD	Modeller (Multimer)	HADDOCK (Refinement)	Symmetry
T82	T0801	HHpred	3EO8/4DN2	Modeller (Multimer)	HADDOCK (Refinement)	Symmetry
T84	T0811	HHpred	1B9B	Modeller	HADDOCK (Refinement)	Symmetry
T85	T0813	HHpred	3GGG	Modeller (Multimer)	HADDOCK (Refinement)	Symmetry
T86	T0815	HHpred	3MJD	Modeller	HADDOCK	Symmetry + CM
T87	T0819	HHpred	3GET	Modeller (Multimer)	HADDOCK (Refinement)	Symmetry + Ca-Ca (PS-HOMPPI)
T88	T0825	HHpred + Needle	2YMU	As above. Mutated[c]	HADDOCK	Symmetry + CM
T89	T0840					
	T0841	HHpred + Needle	1SHY/4FWW	Modeller	HADDOCK	Symmetry + CM
T90	T0843	HHpred	4CL3	Modeller (Multimer)	HADDOCK (Refinement)	Symmetry
T91	T0847	HHpred	1BRY	Modeller/I-Tasser	HADDOCK	Symmetry + Ca-Ca (PS-HOMPPI)
T92	T0849	Delta-Blast	1LBK	Modeller (Multimer)	HADDOCK (Refinement)	Symmetry
T93	T0851	HHpred	2Y0C	Modeller (Multimer)	HADDOCK (Refinement)	Symmetry
T94	T0852	HHpred	3GFF	Modeller (Multimer)	HADDOCK (Refinement)	Symmetry

[a] 2Z80 (res. 27-38) helical restraint (res. 1-16)
[b] Flexible Multibody Docking
[c] in HADDOCK.

15 Zou

Xianjin Xu[1], Liming Qiu[1], Chengfei Yan[1,2], Jilong Li[3], Zhiwei Ma[1,2], Jianlin Cheng[3,4] and Xiaoqin Zou[1,2,4,5,*]

[1] Dalton Cardiovascular Research Center,
[2] Department of Physics and Astronomy,
[3] Department of Computer Science,
[4] Informatics Institute,
[5] Department of Biochemistry,
University of Missouri, Columbia, MO 65211, USA

[*] E-mail: zoux@missouri.edu

The following hierarchical strategy is used for the prediction of all the targets in the CASP-CAPRI round: Starting from a given amino acid sequence, the BLAST program [1] is used to search for homologous proteins with solved 3D structures. If the structures of homologous proteins are available, the Modeller program [2] is used to construct the monomeric structure of the target. Otherwise, a template-free modeling approach employed by the CASP11 MULTICOM-CLUSTER server [3, 4, 5] is used to generate monomeric models. In the meantime, the CASP groups provide a total of 150 best monomeric structures for each target as references for CAPRI groups. Then, these candidates together with our own models are ranked by two different scoring functions. The first scoring function is ITScorePro [6, 7], which we have developed for protein structure selection using an iterative approach to circumvent the reference-state problem in statistical potentials. The other scoring function is APOLLO [8], which is a consensus method based on the average structural similarity between each model and all other models. Having sieved candidate structures by the consensus of these two scoring functions, we further utilize available biological information to control the quality of monomer structure. Finally, about 10 monomeric structures are selected for the subsequent quaternary structure prediction.

Different from previous CAPRI rounds, the number of partners for some targets in Round 30 may reach up to four and most targets have different levels of symmetry. For targets without any symmetry (referred to as heterodimers, similar to the targets in the previous rounds), the FFT-based docking algorithm is used to sample the complex structures. However, for the targets with symmetry, the conventional FFT-based docking is not directly applicable, because of the additional symmetry constraints imposed on the quaternary structure of the targets. Instead, we use geometrical operations [9, 10] to construct decoys consistent with potential symmetries. The resulting decoys are ranked by ITScorePP [11, 12], our atomic pairwise distance-dependent statistical potential-based scoring function for protein-protein structure prediction. The ranked decoys are clustered starting from the decoy with the highest score. Finally, we search for homologous proteins or similar-fold proteins with known quaternary structures by informatics methods and perform molecular dynamics optimization to predict potential native-like protein quaternary structures.

References

[1] S. F. Altschul et al. “Gapped BLAST and PSI-BLAST: a new generation of protein database search programs”. In: *Nucleic Acids Res.* 25.17 (1997), pp. 3389–3402.

[2] M. A. Marti-Renom et al. “Comparative protein structure modeling of genes and genomes”. In: *Annu Rev Biophys Biomol Struct* 29 (2000), pp. 291–325.

[3] Z. Wang, J. Eickholt, and J. Cheng. “MULTICOM: a multi-level combination approach to protein structure prediction and its assessments in CASP8”. In: *Bioinformatics* 26.7 (2010), pp. 882–888.

[4] J. Cheng et al. “The MULTICOM toolbox for protein structure prediction”. In: *BMC Bioinformatics* 13 (2012), p. 65.

[5] J. Li et al. “Designing and benchmarking the MULTICOM protein structure prediction system”. In: *BMC Struct. Biol.* 13 (2013), p. 2.

[6] S. Y. Huang and X. Zou. “Statistical mechanics-based method to extract atomic distance-dependent potentials from protein structures”. In: *Proteins* 79.9 (2011), pp. 2648–2661.

[7] S. Y. Huang and X. Zou. “ITScorePro: an efficient scoring program for evaluating the energy scores of protein structures for structure prediction”. In: *Methods Mol. Biol.* 1137 (2014), pp. 71–81.

[8] Z. Wang, J. Eickholt, and J. Cheng. “APOLLO: a quality assessment service for single and multiple protein models”. In: *Bioinformatics* 27.12 (2011), pp. 1715–1716.

[9] B. Pierce, W. Tong, and Z. Weng. “M-ZDOCK: a grid-based approach for Cn symmetric multimer docking”. In: *Bioinformatics* 21.8 (2005), pp. 1472–1478.

[10] A. Berchanski and M. Eisenstein. “Construction of molecular assemblies via docking: modeling of tetramers with D2 symmetry”. In: *Proteins* 53.4 (2003), pp. 817–829.

[11] S. Y. Huang and X. Zou. “An iterative knowledge-based scoring function for protein-protein recognition”. In: *Proteins* 72.2 (2008), pp. 557–579.

[12] S. Y. Huang and X. Zou. “MDockPP: A hierarchical approach for protein-protein docking and its application to CAPRI rounds 15-19”. In: *Proteins* 78.15 (2010), pp. 3096–3103.

16 Shen

Yang Shen*

Toyota Technological Institute at Chicago, 6045 S Kenwood Avenue, Chicago, IL 60637, USA

Current address: Center for Bioinformatics and Genomic Systems Engineering, Department of Electrical and Computer Engineering, Texas A&M University, College Station, TX 77843, USA

* E-mail: yshen@tamu.edu

Homology Modeling

Homology models were based on CASP11 predictions made available by CASP organizers[13] (stage 2). Specifically, representative models from webservers Rosetta [1, 2] ("BAKER-ROSETTASERVER" from Baker group), I-Tasser [3] ("Zhang-Server" from Zhang group) and RaptorX [4] (from Xu group) were used for 3D structures of monomers.

Docking

Each monomer structure retained was initially docked to itself as a rigid body with webservers ClusPro [5] (symmetry mode, for dimers and trimers) and M-ZDOCK [6] (for quadrumers with C_4 symmetry). Quadrumers with D_2 symmetry were constructed by docking selected first-round C_2 dimers to themselves under C_2 constraints again. Symmetry was thus enforced at the initial stage of rigid-body docking with both servers.

All cluster centers retained from the initial stage were further refined using our own refinement approach [7] with MM-PBSA energy models and the consideration of both backbone and side-chain flexibility. In addition, symmetry was maintained in the refinement stage by sampling only the corresponding subspace of rigid-body motions. In other words, the 5D space of rigid-body motions (a Riemannian manifold of $S^2 \times SO(3)$) after removing the center-to-center distance was again approximated to a Euclidean space with tailored exponential coordinates [8, 9] but only a subset of variables based on these exponential coordinates was needed to respect an oligomer's symmetry during sampling. Sloppy modes to which binding energy was insensitive were again removed while optimizing the resulting conformational space [7].

Scoring

The energetically lowest structural sample was chosen as the final, refined model for each starting oligomer structure. All refined models were again ranked based on the increasing order of binding energy [7]. For few targets whose templates exist as oligomer, the corresponding oligomer templates were sometimes used to promote models in the ranked lists if not already top-ranked.

References

[1] Y. Song et al. "High-resolution comparative modeling with RosettaCM". In: *Structure* 21.10 (2013), pp. 1735–1742.

[2] D. E. Kim, D. Chivian, and D. Baker. "Protein structure prediction and analysis using the Robetta server". In: *Nucleic Acids Res.* 32.Web Server issue (2004), W526–531.

[3] Y. Zhang. "I-TASSER server for protein 3D structure prediction". In: *BMC Bioinformatics* 9 (2008), p. 40.

[4] M. Kallberg et al. "Template-based protein structure modeling using the RaptorX web server". In: *Nat Protoc* 7.8 (2012), pp. 1511–1522.

[5] S. R. Comeau et al. "ClusPro: performance in CAPRI rounds 6-11 and the new server". In: *Proteins* 69.4 (2007), pp. 781–785.

[6] B. Pierce, W. Tong, and Z. Weng. "M-ZDOCK: a grid-based approach for Cn symmetric multimer docking". In: *Bioinformatics* 21.8 (2005), pp. 1472–1478.

[7] Y. Shen. "Improved flexible refinement of protein docking in CAPRI rounds 22-27". In: *Proteins* 81.12 (2013), pp. 2129–2136.

[8] Y. Shen et al. "Optimizing noisy funnel-like functions on the euclidean group with applications to protein docking". In: *Proceedings of the 46th IEEE conference on decision and control.* 2007, pp. 4545–4550.

[9] Y. Shen et al. "Protein docking by the underestimation of free energy funnels in the space of encounter complexes". In: *PLoS Comput. Biol.* 4.10 (2008), e1000191.

[13] http://predictioncenter.org/download_area/CASP11/server_predictions

17 Kihara & LZERD

Lenna X. Peterson[1], Hyung-Rae Kim[1], Amit Roy[1,2], Xusi Han[1], Juan Esquivel-Rodriguez[3] and Daisuke Kihara[1,3,*]

[1] Department of Biological Sciences, Purdue University, West Lafayette, IN 47907, USA
[2] Bioinformatics and Computational Biosciences Branch, Rocky Mountain Laboratories, National Institutes of Health, Hamilton, MT 59840, USA
[3] Department of Computer Science, Purdue University, West Lafayette, IN 47907, USA

* E-mail: dkihara@purdue.edu

Group Kihara

Monomer models were created using our lab's modeling method [1] which is based on CABS [2]. This model was compared against the consensus of models chosen by widely available modeling software (HHPred, SPARKS-X, Phyre2, MULTICOM, TASSER, MUFOLD, TASSERlite, modeled with modeller 9.11). The final monomer model was selected using our scoring method PRESCO [3]. PRESCO is a knowledge-based method to select near native protein models. Within the model, the backbone and side-chain signatures of residues within a sphere are queried against a non-redundant set of native structures to find similar spheres. High amino acid sequence similarity between the model sphere and the retrieved native spheres yields a high score. For some models, the CASP 3D models by Baker and Zhang groups were also used.

Pairwise docking was performed using LZerD [4], our lab's shape-based rigid body docking software. Docking was performed with interface restriction and with no restriction. Prediction of interface residues was performed using BindML from our lab [5] and cons-PPISP [6]. In the case of tetramers, pairwise docking with LZerD was followed by symmetrical docking with M-ZDOCK [7] and Multi-LZerD [8].

Docked models were scored with ITScorePro [9], GOAP [10], and a modified version of PRESCO [3] optimized for selection of docked complexes. Model selection was aided by interface residues of homologs when literature evidence was available.

LZERD

Monomer models were created using our lab's modeling method [1] which is based on CABS [2]. This model uses template based models generated by available modeling software including HHPred, SPARKS-X and Modeller 9.11. Pairwise docking was performed using LZerD [4], our lab's shape-based rigid body docking software. Prediction of interface residues was performed using BindML from our lab [5] and cons-PPISP [6]. Docking was performed with interface restriction and with no restriction. In the case of tetramers, pairwise docking with LZerD was followed by symmetrical multiple docking with M-ZDOCK [7]. Docked models were scored with ITScorePro [9].

References

[1] H. Kim, A. Roy, and D. Kihara. "Structure prediction with CABS model and residue- and fragment-environment potentials". In: (). [14], pp. 98–99.

[2] A. Kolinski. "Protein modeling and structure prediction with a reduced representation". In: *Acta Biochim. Pol.* 51.2 (2004), pp. 349–371.

[3] H. Kim and D. Kihara. "Detecting local residue environment similarity for recognizing near-native structure models". In: *Proteins* 82.12 (2014), pp. 3255–3272.

[4] V. Venkatraman et al. "Protein-protein docking using region-based 3D Zernike descriptors". In: *BMC Bioinformatics* 10 (2009), p. 407.

[5] D. La and D. Kihara. "A novel method for protein-protein interaction site prediction using phylogenetic substitution models". In: *Proteins* 80.1 (2012), pp. 126–141.

[6] H. X. Zhou and Y. Shan. "Prediction of protein interaction sites from sequence profile and residue neighbor list". In: *Proteins* 44.3 (2001), pp. 336–343.

[7] B. Pierce, W. Tong, and Z. Weng. "M-ZDOCK: a grid-based approach for Cn symmetric multimer docking". In: *Bioinformatics* 21.8 (2005), pp. 1472–1478.

[8] J. Esquivel-Rodriguez, Y. D. Yang, and D. Kihara. "Multi-LZerD: multiple protein docking for asymmetric complexes". In: *Proteins* 80.7 (2012), pp. 1818–1833.

[9] S. Y. Huang and X. Zou. "Statistical mechanics-based method to extract atomic distance-dependent potentials from protein structures". In: *Proteins* 79.9 (2011), pp. 2648–2661.

[10] H. Zhou and J. Skolnick. "GOAP: a generalized orientation-dependent, all-atom statistical potential for protein structure prediction". In: *Biophys. J.* 101.8 (2011), pp. 2043–2052.

[14] http://predictioncenter.org/casp11/doc/CASP11_Abstracts.pdf

18 Wade

Xiaofeng Yu[1], Neil J. Bruce[1], Jonathan C. Fuller[1] and Rebecca C. Wade[1,2,3,*]

[1] Molecular and Cellular Modeling group, Heidelberg Institute for Theoretical Studies (HITS), Heidelberg, Germany
[2] Center for Molecular Biology (ZMBH), DKFZ-ZMBH Alliance, Heidelberg University, Heidelberg, Germany
[3] Interdisciplinary Center for Scientific Computing (IWR), Heidelberg University, Heidelberg, Germany

* E-mail: rebecca.wade@h-its.org

We submitted docked complex solutions for two targets of CAPRI round 30, targets 68 and 69.

How was the structure of the individual subunits modeled?

We used **CASP models** for unbound proteins.

Which modeling software was used?

The software package Simulation of Diffusional Association (**SDA**[15]) was used to perform protein-protein docking. The selected complexes were further refined using all-atom molecular dynamics in **Amber 12**.

Was any of the available CASP 3D structure predictions used?

For both targets, we used monomer structures that were released from CASP as starting points. All released models were assessed using the QMEAN server[16] and the three highest scoring were selected. For target 68 these were: QUARK_TS1, FALCON_EnvFold_TS4 and FALCON_TOPO_TS2. The loop regions of the two termini were cut (first 8 residues of the N-terminus and last 9 residues of the C-terminus). For target 69, they were: Zhang-server_TS1, QUARK_TS1 and QUARK_TS3, with the complete models.

Which docking and scoring methods were applied; were symmetry constraints imposed? Was information on homologs used?

SDA performs rigid-body Brownian dynamics simulations using precomputed interaction grids. For the docking simulations described here, we used grids describing the **electrostatic, electrostatic desolvation and hydrophobic desolvation interactions** between interacting proteins.

The docking method in SDA models bimolecular diffusional association and records predicted encounter complexes when a predefined reaction criterion is satisfied. In this case, the reaction criterion was defined such that the centers of geometry of the interacting proteins are closer than the sum of the distances from the centers of each protein to their furthest surface atoms, plus 10 Å. The 2000 highest scoring recorded complexes, as scored by the SDA forcefield, were then clustered into 10 clusters using an average-linkage clustering algorithm. These clusters were then ranked by the number of complexes in the cluster, and the cluster representatives from the top five clusters were chosen for further refinement.

The **Amber ff99SB forcefield** [1] was used and the solvent environment was described using the modified OBC Generalized-Born model [2]. The complexes were subjected to 1000 steps of minimization, followed by 500 ps of molecular dynamics.

The similarity to the homologous model was used to decide the rank of complexes for submission in the case of both targets.

References

[1] V. Hornak et al. “Comparison of multiple Amber force fields and development of improved protein backbone parameters”. In: *Proteins* 65.3 (2006), pp. 712–725.

[2] A. Onufriev, D. Bashford, and D. A. Case. “Exploring protein native states and large-scale conformational changes with a modified generalized born model”. In: *Proteins* 55.2 (2004), pp. 383–394.

[15] http://mcm.h-its.org/sda7/doc/doc_sda7/index.html
[16] http://swissmodel.expasy.org/qmean/cgi/index.cgi

19 Vakser

Ivan Anishchenko[1], Petras J. Kundrotas[1,◇] and Ilya A. Vakser[1,2,*]

[1] Center for Computational Biology,
[2] Department of Molecular Biosciences,
The University of Kansas, Lawrence, KS 66047, USA

◇ E-mail: pkundro@ku.edu
* E-mail: vakser@ku.edu

The number of experimentally determined protein structures and their complexes accounts only for a fraction of known protein “universe”. Thus, structural modeling of protein-protein interactions (docking) largely has to rely on modeled structures of the individual proteins [1]. This round of CASP was important for testing our ability to do that by utilizing docking methods within CAPRI assessment framework.

Methods

For the template-based docking we used the protocol similar to the one developed previously in our lab [2, 3]. The procedure performs spatial rearrangement of 3D structures of the two target proteins (treated as rigid bodies) to match either the entire monomers of the co-crystallized complexes (from the full-structure template library) or their interfaces only (from the interface template library). Structural alignment of the proteins was performed by TM-align [4]. The free docking was performed by our GRAMM program implementing the FFT approach [5, 6]. To accommodate the structural inaccuracies of the modeled monomers, the program was run at lower resolution.

Results

Monomer models were chosen from 150 server models provided by CASP organizers after visual inspection (to exclude poorly packed structures with long misfolded termini, loops, etc.) and clustering of the models based on their structural similarity. For targets 68–70, top five models generated by the stand-alone I-TASSER package (Yang Zhang lab) were selected. Dimeric complexes were built by full and partial structure alignment techniques using generic and/or specialized template libraries, previously generated in our lab. For targets 85, 89, 92, and 93 additional templates (previously filtered out from our libraries due to structural deficiencies) with sequence similarity to the target proteins (detected by BLAST) were extracted from the PDB. All full and partial structure alignment models were evaluated in terms of the TM-score, fraction of interface residue contacts shared between the target and the template F_cont, and relative volume of clashes V. Models satisfying condition (TM-score > 0.6 OR F_cont > 0.1) AND ($V < 0.05$) were clustered based on structural similarity and ten representative models from the most populated clusters were chosen for the final minimization. Tetrameric targets (70, 71, 73, 74, 78, and 81) were built from the dimers by free docking using GRAMM program in the low-resolution mode.

Availability

The docking procedures and libraries used in this round are available from our Dockground resource for protein recognition studies[17].

References

[1] I. A. Vakser. “Low-resolution structural modeling of protein interactome”. In: *Curr. Opin. Struct. Biol.* 23.2 (2013), pp. 198–205.

[2] R. Sinha, P. J. Kundrotas, and I. A. Vakser. “Docking by structural similarity at protein-protein interfaces”. In: *Proteins* 78.15 (2010), pp. 3235–3241.

[3] P. J. Kundrotas and I. A. Vakser. “Global and local structural similarity in protein-protein complexes: implications for template-based docking”. In: *Proteins* 81.12 (2013), pp. 2137–2142.

[4] Y. Zhang and J. Skolnick. “TM-align: a protein structure alignment algorithm based on the TM-score”. In: *Nucleic Acids Res.* 33.7 (2005), pp. 2302–2309.

[5] E. Katchalski-Katzir et al. “Molecular surface recognition: determination of geometric fit between proteins and their ligands by correlation techniques”. In: *Proc. Natl. Acad. Sci. U.S.A.* 89.6 (1992), pp. 2195–2199.

[6] I. A. Vakser. “Protein docking for low-resolution structures”. In: *Protein Eng.* 8.4 (1995), pp. 371–377.

[17] http://dockground.compbio.ku.edu/

20 Tomii

Kenichiro Imai[1,†], Kazunori Yamada[1,‡], Toshiyuki Oda[1,†], Tsukasa Nakamura[2] and Kentaro Tomii[1,2,†,*]

[1] Computational Biology Research Center (CBRC), National Institute of Advanced Industrial Science and Technology (AIST), Koto-ku, Japan
[2] Graduate School of Frontier Sciences, The University of Tokyo, Kashiwa, Japan

Current affiliations:
[†] Biotechnology Research Institute for Drug Discovery, National Institute of Advanced Industrial Science and Technology (AIST), Koto-ku, Japan
[‡] Group of Electrical Engineering, Communication Engineering, Electronic Engineering, and Information Engineering, Tohoku University, Sendai, Japan

[*] E-mail: k-tomii@aist.go.jp

Methodologies for constructing and selecting three-dimensional protein complex models for CAPRI round 30

We used the following four steps to predict protein complexes for the CAPRI R30 targets, as in CASP11. i) FORTE series [1], including DELTA-FORTE which is our new profile-profile alignment method empowered by NCBI's Conserved Domain Database (CDD) (unpublished), are performed with the PDB and SCOP libraries for each target to obtain target-template alignments. In addition, we have recently derived a novel amino acid substitution matrix, MIQS [2]. This matrix also uses auxiliaries in searching for templates and sampling alignments. ii) Based on those alignments of top 100 proteins, we built 3D models with MODELLER and MOE (Molecular Operating Environment). For building 3D-models, we employed multiple templates when we were able to use structural information of the same family in PDB. iii) We sorted our models in terms of the structural quality scores calculated by the Verify3D program. As a precise guidance of model selection, we utilized the scores, which were averaged over models based on an alignment, instead of a score for each model. The averaged score is effective to enhance prediction accuracy, especially for easy targets, according to our preliminary results. These procedures are mostly executed as an individual subunit basis. iv) Then we observed oligomeric states of top candidates sorted by their structural quality scores to predict three-dimensional protein complex models. In many cases we could see similar tendency of oligomeric states among top candidates for each target. Otherwise, we calculated structural quality scores of protein complex models to select proper one. We did not impose symmetry constraints to build/select protein complex models, with a few exceptions.

References

[1] K. Tomii, T. Hirokawa, and C. Motono. “Protein structure prediction using a variety of profile libraries and 3D verification”. In: *Proteins* 61 Suppl 7 (2005), pp. 114–121.

[2] K. Yamada and K. Tomii. “Revisiting amino acid substitution matrices for identifying distantly related proteins”. In: *Bioinformatics* 30.3 (2014), pp. 317–325.

21 Fernández-Recio

Chiara Pallara, Miguel Romero-Durana, Brian Jiménez-García, Iain H. Moal and Juan Fernández-Recio*

Joint BSC-CRG-IRB Research Program in Computational Biology, Barcelona Supercomputing Center, C/ Jordi Girona 29, 08034 Barcelona, Spain

* E-mail: juanf@bsc.es

The goal here was to use docking to model the quaternary structure of the proposed targets. Since the 3D structures of the individual subunits needed to be modelled, we used as input structures for docking a set of models formed by the top five CASP submissions (when available) from the ZHANG, ROSETTA and QUARK servers. For the heterodimer (T89) we used for each subunit only the best model from these servers according to Z-DOPE score. For docking, different protocols were applied to these 15 starting models depending on the oligomeric state of the target. For all dimeric targets, FTDock (with electrostatics and 0.7 Å grid resolution) and ZDOCK 2.1 were used to generate 10,000 and 2,000 rigid-body docking poses, respectively, forming homo- or hetero-dimers depending on the case. These docking solutions were merged and scored using the pyDock binding energy. No symmetry restraints were used in these targets. For the homotetrameric targets, Symmdock and CombDock were applied to generate symmetric and non-symmetric docking poses, respectively. The total binding energy of all possible interfaces was then calculated using pyDock, and the final models were selected so that the symmetric and non-symmetric solutions were evenly represented. For the dimer of heterodimers (T81), SymmDock was used to generate symmetric homodimeric docking poses for each of the two different subunits, while FTDock and ZDock were applied to obtain heterodimeric docking orientations between the different subunits. All the complexes were finally scored with pyDock. The top 1000 docking poses of each pool were combined together and filtered to remove solutions without spatial compatibility or lacking symmetry between heterodimers. The final docking solutions were scored according to the total complex binding energy calculated on all interfaces using pyDock. For all the targets, redundant predictions were eliminated, and the final 10 selected docking poses were minimized to reduce the number of interatomic clashes using AMBER10 with AMBER parm99 force field. No restraints on potential interface residues based on biological information were used for any of the targets.

22 Lee

Jong Young Joung[1], Jong Yun Kim[1], Keehyoung Joo[1,2], and Jooyoung Lee[1,3,*]

[1] Center for In-Silico Protein Science,
[2] Center for Advanced Computation,
[3] School of Computational Science,
Korea Institute for Advanced Study, 130-722, Korea

* E-mail: jlee@kias.re.kr

Template-based structure modeling of protein-protein interactions by global optimization

We have developed a protein-protein modeling system extended from our prediction platform of CASP protocols. In this CAPRI round, the modeling of monomer subunits is required prior to the construction of complex structures. The model1 structure of the nns server is used as the subunit to be assembled. The nns server applied the global optimization method of conformational space annealing (CSA) to three stages of optimization including multiple sequence -structure alignment, 3D chain building, and side-chain remodeling.

Templates for the complex under consideration were collected from the list of homo-multimeric templates used for the template-based modeling of the subunit. We performed structure alignment using TM-align to calculate the structural similarity between the nns model1 and each component in the complex template. Templates with TM-score $>$ 0.5 were selected and clustered based on the binding interface similarity. The binding interface is a set of residue-residue pairs that have at least one heavy atom pair within the distance of 5.0 Å. For each complex template, the interface residue pairs were identified and the set of interface residue pairs were used for constructing a network consisting of nodes of templates and edges among nodes properly weighted with the binding interface similarity. Clustering is performed by applying the modularity optimization software of Mod-CSA to the network. Using all complex templates in a cluster, appropriate inter- and intramolecular atom pair distance information is collected to generate template-derived restraint energy terms. MODELLER is used to generate initial model structures of protein-protein complexes.

To refine complex structures, we applied CSA to optimize the protein-protein interaction potential that consists of stereochemistry energy, DFIRE, torsion-torsion interaction energy, repulsion energy of van der Waals interaction, DCOMPLEX and template-derived restraint energy. For the template-derived restraint energy described above, we used the Lorentzian-function-based penalty term. The inter-molecular portion of the template-derived restraint term and the DCOMPLEX energy were used to adjust the binding mode among subunits in the complex structure, and the other energy components were used to settle the subunit structure during the optimization process to represent the induced fit. We did not use any information of symmetry between asymmetric units.

23 CLUSPRO

Dima Kozakov[1,*], Sandor Vajda[1,2,◇], Scott Mottarella[1], David R. Hall[1], Dmitri Beglov[1], Artem Mamonov[1], Bing Xia[1] and Tanggis Bohnuud[1]

Departments of [1]Biomedical Engineering and [2]Chemistry, Boston University

* E-mail: midas@bu.edu
◇ E-mail: vajda@bu.edu

Prediction of the probable biological units using the ClusPro server

Introduced in 2004, ClusPro was the first fully automated web based server for protein-protein docking. The server performs rigid body docking using the PIPER program and clusters the lowest energy structures. The models are ranked according to cluster size. In order to deliver results to the user within 24 hours of submission, the current implementation of ClusPro does not include refinement beyond minimizing the energy of structures to remove steric overlaps. In spite of this limitation, the server has almost 4800 registered users, and runs about 4000 jobs each month.

In the latest round of CASP-CAPRI experiment we have applied ClusPro for the structure prediction of the probable biological units for the "easy" CASP targets.

Methods

Model preparation

Since ClusPro requires three dimensional structure as an input, we have built a "consensus" model for each target using the 150 server models provided by the CASP management committee. For each "easy" target most models had the same fold, with variations in loops and tails. Removal of the uncertain regions resulted in reliable "consensus" models that were used for docking.

Docking

Our docking approach consists of two steps. The first step is running PIPER – docking program that performs systematic search of complex conformations on a grid using the fast Fourier transform (FFT) correlation approach. The scoring function includes van der Waals interaction energy, an electrostatic energy term, and desolvation contributions calculated by a pairwise potential.

The second step of the algorithm is clustering the top 1000 structures generated by PIPER using pairwise RMSD as the distance measure. The radius used in clustering is defined in terms of Cα interface RMSD. For each docked conformation we select the residues of the ligand that have any atom within 10 Å of any receptor atom, and calculate the Cα RMSD for these residues from the same residues in all other 999 ligands. Thus, clustering 1000 docked conformations involves computing a 1000×1000 matrix of pairwise Cα RMSD values. Based on the number of structures that a ligand has within a (default) cluster radius of 9 Å RMSD, we select the largest cluster and rank its cluster center as number one. The members of this cluster are removed from the matrix, and we select the next largest cluster and rank its center as number two, and so on. After clustering with this hierarchical approach, the ranked complexes are subjected to a straightforward (300 step and fixed backbone) van der Waals minimization using the CHARMM potential to remove potential side chain clashes. Unless requested otherwise by the user, ClusPro outputs the centers of the 10 largest clusters, which were submitted as predictions.

24 Del Carpio

Carlos A. Del Carpio[1,2,*] and Eichiro Ichiishi[3]

[1] Institute of Biological Diversity, International Pacific Institute of Indiana, P.O. Box 7304, Bloomington, IN 47401 USA
[2] Drosophila Genetic Resource Center, Kyoto Institute of Technology, Saga Ippongi-cho, Ukyo-ku 616-8354, Japan
[3] International University of Health and Welfare Hospital (IUHW Hospital), 537 Iguchi, Asushiobara-city, Tochigi Pref. 329-2763, Japan

* E-mail: carlos@aki.che.tohoku.ac.jp

Protein-Protein Docking Using MIAX in the joins CASP-11 – CAPRI Experiment

For prediction of the interaction targets in CASP-11 our group used mainly the system MIAX [1, 2, 3, 4, 5] for docking unbound structures. The structures used in the docking process were namely those that were predicted by our own group.

MIAX (Macromolecular Interaction Assessment Computer System) is a combination of several Methodologies to dock rigid protein structures but with flexible and softening techniques to mimic real induced fit processes during the docking operation. MIAX outputs several thousands of decoys that are selected based upon a priori recognition of potential active sites on the surface of the proteins. We have developed a systematic methodology to recognize putative binding sites on the protein surfaces based on quantification of the hydrophobic potential. The system has been benchmarked on hundreds of PDB protein complexes, and the reliability of the predictions is more than 70%.

Since the structures to dock in the case of CASP-11 are deemed to be of low resolution, the methodology to soften the surfaces in MIAX is very adequate to average positions that may not exactly be those of the crystal structure.

Since the number of decoys after the first docking process in MIAX exceeds the 4000 structures, these are clustered and those clusters holding close-to-predicted binding areas are ranked high in this initial step.

To refine the final complex structures we have used namd2 molecular dynamics program to equilibrate and minimize the energy of the structures. Our group submitted candidates for all the CASP-11 targets.

References

[1] C. A. Del Carpio-Munoz et al. “MIAX: a new paradigm for modeling biomacromolecular interactions and complex formation in condensed phases”. In: *Proteins* 48.4 (2002), pp. 696–732.

[2] C. A. Del Carpio et al. “A graph theoretical approach for assessing bio-macromolecular complex structural stability”. In: *J Mol Model* 15.11 (2009), pp. 1349–1370.

[3] C. A. Del Carpio Munoz et al. “Docking unbound proteins with MIAX: a novel algorithm for protein-protein soft docking”. In: *Genome Inform* 14 (2003), pp. 238–249.

[4] C. A. Del Carpio et al. “Robotic path planning and protein complex modeling considering low frequency intra-molecular loop and domain motions”. In: *Genome Inform* 17.2 (2006), pp. 270–278.

[5] C. A. Del Carpio M et al. “A graph theoretical approach for analysis of protein flexibility change at protein complex formation”. In: *Genome Inform* 16.2 (2005), pp. 148–160.

25 Gray

Nicholas Marze[1], Daisuke Kuroda[1,†,‡], Shourya S. Roy Burman[1,‡] and Jeffrey J. Gray[1,2,*]

[1] Department of Chemical and Biomolecular Engineering and [2] Program in Molecular Biophysics
Johns Hopkins University, Baltimore, MD 21218, USA

† The authors contributed equally.
‡ Current address: School of Pharmacy, Showa University, Shinagawa-ku, Tokyo 142-8555, Japan

* E-mail: jgray@jhu.edu

Preparation for Scoring

Since the scoring structures were created by a variety of protocols, a short relaxation under the Rosetta force field was necessary for fair comparison of structures. For each scoring target, we trimmed the candidate structures to one or two consensus sequences. We discarded structures shorter than the consensus sequence or mismatched to the consensus sequence. The remaining candidate structures were prepacked for Rosetta scoring by repacking the side chains with a fixed backbone. We optimized the large complexes (larger than ~600 residues) using three rounds of RosettaDock local refinement [1, 2] – iterations of small-magnitude rigid-body, side-chain repacking, and gradient-based minimization with a Monte-Carlo acceptance criterion. We followed the docking protocol with a single round of Rosetta FastRelax [3] – five iterations of all-atom side-chain repacking and minimization, where the Van der Waals repulsive forces are ramped up in each iteration from 2% to 100% of the final value. We optimized the small complexes (smaller than 600 residues) using 10–20 rounds (depending on time constraints) of Rosetta FastRelax.

The Rosetta protocols were used as follows:
For large complexes,

1. fixbb.static.linuxgccrelease -nstruct 1 -resfile <location> -out:file:fullatom -ex1 -ex2aro -use_input_sc *(Prepacking)*
2. docking_protocol.static.linuxgccrelease -nstruct 3 -docking_local_refine -out:file:fullatom -ex1 -ex2aro -use_input_sc *(RosettaDock)*
3. relax.static.linuxgccrelease -in:file:fullatom -out:file:fullatom -relax:fast -nstruct 1 -ex1 -ex2aro -use_input_sc -relax:jump_move *(FastRelax)*

For small complexes,

1. fixbb.static.linuxgccrelease -nstruct 1 -resfile <location> -out:file:fullatom -ex1 -ex2aro -use_input_sc *(Prepacking)*
2. relax.static.linuxgccrelease -in:file:fullatom -out:file:fullatom -relax:fast -nstruct 20 -ex1 -ex2aro -use_input_sc -relax:jump_move *(FastRelax)*

Scoring

We evaluated the prepared candidate structures using three different scores: total score, interface score, and elliptic score. Total score is analogous to the free energy of the complex; it was calculated by applying the RosettaDock score function [2] to the candidate complexes. Interface score is analogous to the binding energy of the complex; it was calculated by separating the partners in the complex, scoring the separated complex, and subtracting the result from the total score. Elliptic score is a combined metric of the total and interface scores, calculated by an elliptical transformation of the two [4].

Selection of Structures

For each consensus sequence, we identified the set of candidate structures with the ten lowest scores, ten lowest interface scores, and ten lowest elliptic scores. In some cases, we manually added more low-scoring structures distinct from the automated pool. We then manually filtered the resultant pool, generally comprising twenty to fifty unique structures. We removed any candidate complexes that had poorly folded secondary structure, very small interfaces, or asymmetry (for homomultimeric complexes) from the pool. We clustered by eye the remaining candidate structures, and for submission we selected ten structures from unique clusters, with the lowest-scoring structures by each scoring method represented by two to five structures each. If we did not find ten distinct clusters, we supplemented the submission set with low-scoring candidate structures from duplicate clusters.

References

[1] J. J. Gray et al. "Protein-protein docking with simultaneous optimization of rigid-body displacement and side-chain conformations". In: *J. Mol. Biol.* 331.1 (2003), pp. 281–299.

[2] S. Chaudhury et al. "Benchmarking and analysis of protein docking performance in Rosetta v3.2". In: *PLoS ONE* 6.8 (2011), e22477.

[3] M. D. Tyka et al. "Alternate states of proteins revealed by detailed energy landscape mapping". In: *J. Mol. Biol.* 405.2 (2011), pp. 607–618.

[4] K. P. Kilambi et al. "Extending RosettaDock with water, sugar, and pH for prediction of complex structures and affinities for CAPRI rounds 20-27". In: *Proteins* 81.12 (2013), pp. 2201–2209.

26 Oliva

Edrisse Chermak[1], Luigi Cavallo[1] and Romina Oliva[2,*]

[1] King Abdullah University of Science and Technology, Saudi Arabia
[2] University of Naples “Parthenope”, Italy

* E-mail: romina.oliva@uniparthenope.it

We submitted scoring predictions for 22 out of the 25 assessed targets. For each target, CONSRANK [1, 2] was run on the ensemble of uploaders' models, after they had been edited to assign to corresponding amino acids the same chain identifier and number. The automatic renumbering tool we developed to the aim first extracts the FASTA sequences from the PDB files, then uses BLASTclust [3] to distinguish the different chains and aligns sequences within each cluster with ClustalW [4]; finally, it rewrites the PDB files to have the sequences consistently renumbered. The ten models top ranked by CONSRANK and not showing a number of clashes above the CAPRI-defined threshold were selected for submission to the scoring session. CONSRANK is a consensus-based algorithm, which ranks models based on their ability to match the most conserved contacts in the ensemble they belong to. Specifically, given an ensemble of N models of the same complex, for each inter-residue contact the conservation rate, CR_{kl} is defined as:

$$CR_{kl} = nc_{kl}/N,$$

where nc_{kl} is the total number of models where residues k and l are in contact. The conservation rate thus ranges between $CR_{kl} = 0$, if the contact between residues k and l is never observed, and $CR_{kl} = 1$, if the contact is observed in all the models. Once the conservation rates have been calculated, for each model i a score is first calculated as:

$$S_i = \sum_{1}^{M_i} CR_{kl},$$

where M_i is the total number of contacts in model i. Then, a normalized score, $\acute{S}_i$, is calculated as:

$$\acute{S}_i = \frac{S_i}{M_i}.$$

Models are ranked according to their $\acute{S}_i$ value. Two residues are defined in contact if any pair of atoms belonging to the two residues is closer than a cut-off distance of 5 Å. The stability of the method to cut-off distances in the range 4 to 10 Å has been previously demonstrated [5].

Availability

CONSRANK and the renumbering tool are freely available as web services at:
https://www.molnac.unisa.it/BioTools/consrank/ [5].

References

[1] R. Oliva, A. Vangone, and L. Cavallo. “Ranking multiple docking solutions based on the conservation of inter-residue contacts”. In: *Proteins* 81.9 (2013), pp. 1571–1584.

[2] A. Vangone, L. Cavallo, and R. Oliva. “Using a consensus approach based on the conservation of inter-residue contacts to rank CAPRI models”. In: *Proteins* 81.12 (2013), pp. 2210–2220.

[3] S. F. Altschul et al. “Gapped BLAST and PSI-BLAST: a new generation of protein database search programs”. In: *Nucleic Acids Res.* 25.17 (1997), pp. 3389–3402.

[4] J. D. Thompson, D. G. Higgins, and T. J. Gibson. “CLUSTAL W: improving the sensitivity of progressive multiple sequence alignment through sequence weighting, position-specific gap penalties and weight matrix choice”. In: *Nucleic Acids Res.* 22.22 (1994), pp. 4673–4680.

[5] E. Chermak et al. “CONSRANK: a server for the analysis, comparison and ranking of docking models based on inter-residue contacts”. In: *Bioinformatics* 31.9 (2015), pp. 1481–1483.

27 Tovchigrechko & GRAMM-X

Andrey Tovchigrechko

J. Craig Venter Institute, 9704 Medical Center Drive, Rockville, MD 20850, USA

* E-mail: atovtchi@jcvi.org

The structures of individual subunits were modeled with I-TASSER modeling package v.2 [1], installed on a local compute cluster. The models were inspected in PyMOL v.1.7 together with aligned template structures and trimmed where deemed necessary. None of the available CASP 3D structure predictions were used.

GRAMM-X software [2] and Web server [3] were used for docking. Symmetry constraints as implemented in GRAMM-X were used for homo-polymer targets. Briefly, every low-energy candidate space transformation coming out of the DFFT grid search is checked that it is close to be symmetrical within a certain cutoff, and, having passed that check, the candidate model is subject to the GRAMM-X off-grid semi-local refinement step under symmetry constraints.

Information on available homologs of the target complexes was not used.

GRAMM-X

GRAMM-X is a collaboration between Andrey Tovchigrechko and Ilya Vakser. It is available at:
http://vakser.compbio.ku.edu/resources/gramm/grammx

References

[1] D. Xu et al. “Automated protein structure modeling in CASP9 by I-TASSER pipeline combined with QUARK-based ab initio folding and FG-MD-based structure refinement”. In: *Proteins* 79 Suppl 10 (2011), pp. 147–160.

[2] A. Tovchigrechko and I. A. Vakser. “Development and testing of an automated approach to protein docking”. In: *Proteins* 60.2 (2005), pp. 296–301.

[3] A. Tovchigrechko and I. A. Vakser. “GRAMM-X public web server for protein-protein docking”. In: *Nucleic Acids Res.* 34.Web Server issue (2006), W310–314.

Evaluation protocol for higher order oligomers

Submitted models

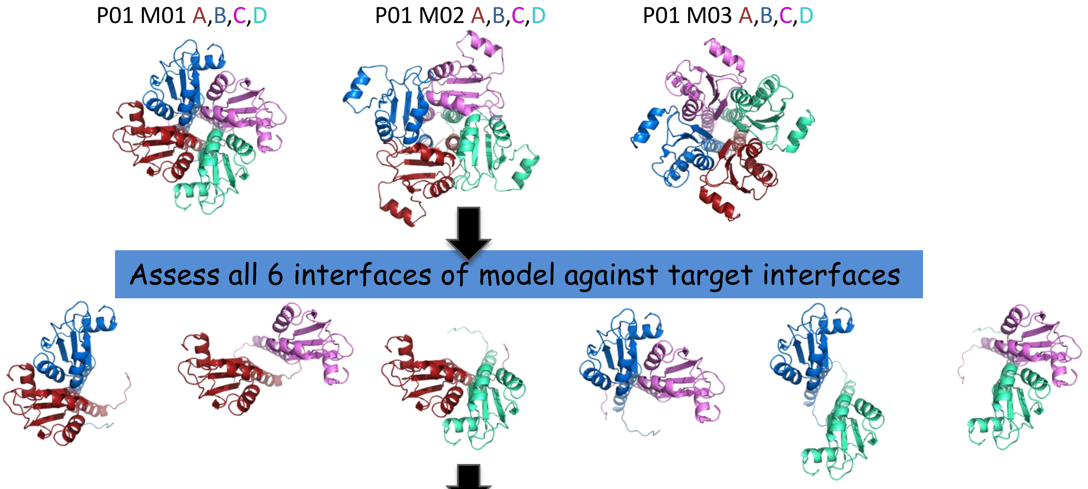

Select best predicted interface

Tetramer	Dimer	$f_{(nat)}$	L-rms	Selected model
A:B:C:D	A:B	0.000	58.4	
	A:C	0.000	53.9	
	A:D	**0.150**	**9.5**	**A:D**
	B:C	0.068	12.4	
	B:D	0.140	9.8	
	C:D	0.000	44.6	

Figure S1 – Evaluation protocol for higher-order oligomers. We evaluate the prediction quality of each interface of the higher-order oligomers individually. In order to do so, we extract all interfaces in the prediction and assess these against the target interfaces. For a tetrameric model as depicted here, consisting of chains A, B, C and D, we extract the dimeric pairs AB, AC, AD, BC, BD and CD. An N-model submission then effectively becomes a 6N-model submission, giving rise to 6N sets of assessment quantities f(nat), L-rms and I-rms. Of each 6 pairs corresponding to the original tetrameric submission, the best assessment quantities are selected and the original submission is annotated with these. First the highest f(nat) is selected; if these are equal, the lowest L-rms is taken, followed by lowest I-rms if necessary.

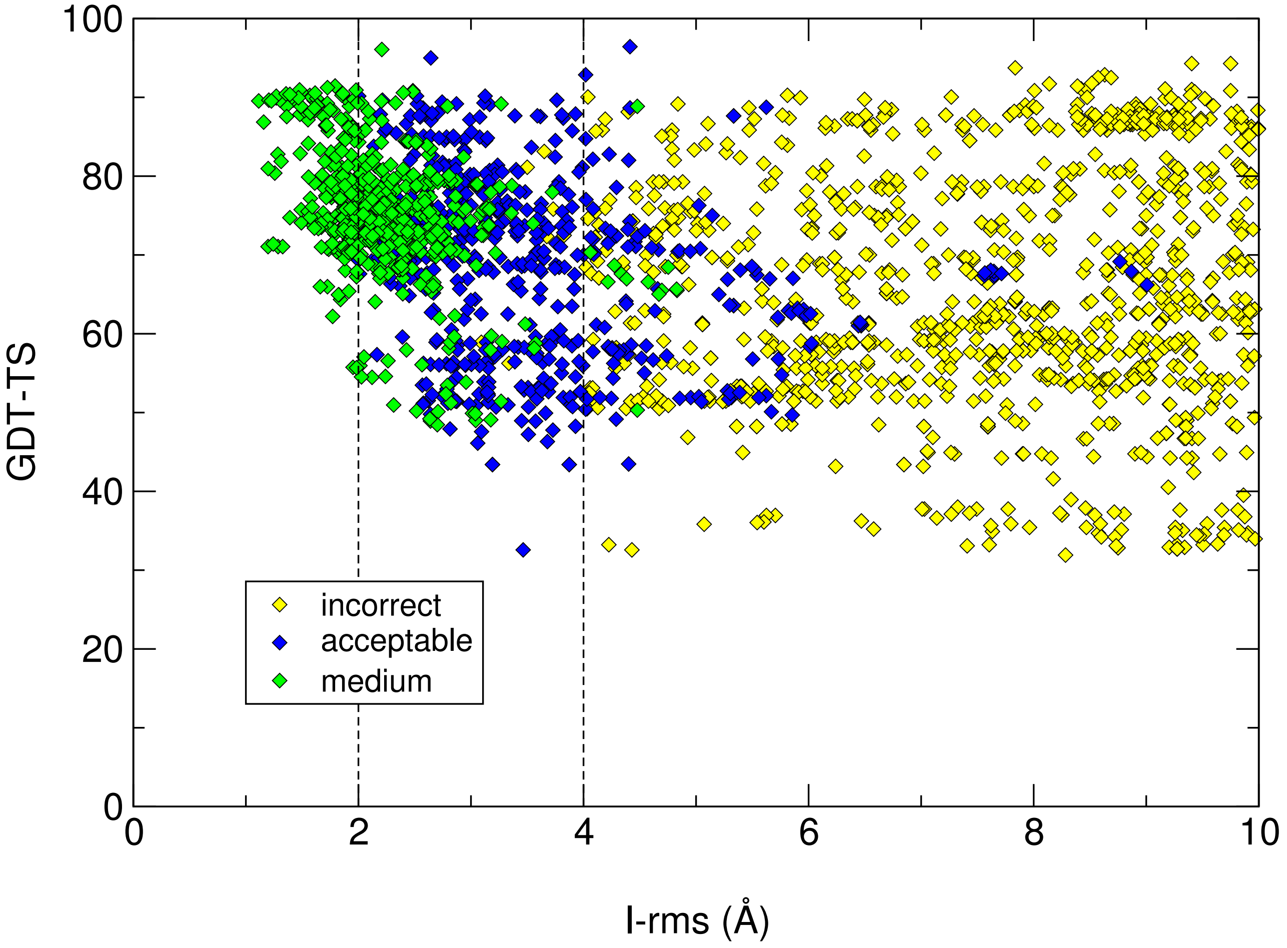

Figure S2 – Subunit model accuracy as measured using the GDT-TS score and the quality of predicted complexes in CAPRI Round 30. The CASP GDT-TS scores of subunit models in the predicted complexes for the 25 targets in this Round (vertical axis) are plotted as a function of the I-rms values (horizontal axis) of the complexes. Each point in this Figure represents one submitted model, and points are colored according to the quality of the predicted complex, respectively, incorrect (yellow), acceptable (blue) and medium (green) quality (see Table 3 and the text for details). The plotted GDT-TS values are for predicted complexes for which the LGA-S and GDT-TS scores differ by less than 15 units (about 80% of the submitted models), to ascertain that the corresponding subunits had the correct residues numbering. Given that by and large the Round 30 CAPRI targets represented proteins that could be easily modeled by homology, the chances that models with high values for both GDT-TS and LGA-S scores correspond to different structural alignments is extremely low.
